# Global prevalence trends and predictions of Hodgkin lymphoma from 1990 to 2021: age-period-cohort analysis and transnational inequality

**DOI:** 10.7189/jogh.16.04199

**Published:** 2026-09-11

**Authors:** Wei-Li Wang, Shen-Sui Li, Dan Ma, Shi-Si Huang, Xing-Yi Kuang, Zheng-Long Tang, Qin Fang, Ji-Shi Wang

**Affiliations:** 1 Department of Hematology, The Affiliated Hospital of Guizhou Medical University, Guiyang, Guizhou, China; 2 Hematopoietic Stem Cell Transplantation Center of Guizhou Province, Key Laboratory of Hematological Disease Diagnostic & Treat Centre of Guizhou Province, The Affiliated Hospital of Guizhou Medical University, Guiyang, Guizhou, China; 3 Department of Oral and Maxillofacial Surgery, Affiliated Stomatological Hospital of Guizhou Medical University, Guiyang, Guizhou, China; 4 Departmen of Pharmacy, The Affiliated Hospital of Guizhou Medical University, Guiyang, Guizhou, China; 5 Department of Hematology, Guiqian International Hospital, Guiyang, Guizhou, China

**Keywords:** Hodgkin lymphoma, GBD, mortality, DALYs, ASR, prevalence

## Abstract

**Background:**

Hodgkin lymphoma (HL) is a highly curable malignancy with distinctive aetiological subtypes. Given recent therapeutic advances and demographic changes, an up-to-date assessment of the global HL burden is needed to inform evidence-based resource allocation and health policy planning. This study described the global prevalence trend of HL and analysed its age cohort and transnational inequality.

**Methods:**

We used data from the Global Burden of Disease 2021 study (204 countries/territories). We analysed age-standardised rates (ASRs) for incidence, prevalence, mortality, and disability-adjusted life-years (DALYs) from 1990 to 2021. An autoregressive integrated moving average (ARIMA) model projected incidence trends to 2050. We used the age-period-cohort (APC) analysis to separate the effects of age, period, and cohort on HL burden. We assessed transnational inequality with the slope index of inequality and the concentration index. All estimates are reported with 95% uncertainty intervals (UI).

**Results:**

In 2021, an estimated 65,182 (95% UI = 53,167, 77,143) new HL cases occurred globally. Substantial geographic disparities were observed in ASRs per 100,000 population: prevalence 4.42 (95% UI = 3.75, 5.09), incidence 0.79 (95% UI = 0.64, 0.94), mortality 0.34 (95% UI = 0.25, 0.43), and DALYs 14.82 (95% UI = 10.43, 18.96). The global age-standardised incidence rate (ASIR) declined slightly (estimated annual percentage change = −1.10%; 95% confidence interval (CI) = −1.16, 1.05) from 1990 to 2021, yet absolute incident cases increased by 19.23% (95% CI = 14.14, 22.44), driven by population growth and ageing. We found that HL risk peaked among young (25–29 years) and older adults (65–69 years), with steady declines in relative risk across successive periods and birth cohorts. The burden consistently skewed towards males and high sociodemographic index (SDI) regions. Projections indicated a stable ASIR but a rising prevalence through 2050, alongside continued declines in mortality and DALY rates.

**Conclusions:**

We found that the global HL burden is characterised by stable ASIR but growing absolute case numbers, with a disproportionate impact on males and high-SDI countries. These trends, reflecting demographic shifts and therapeutic advances, underscore the necessity for robust diagnostic pathways and multidisciplinary, risk-adapted care models to manage the increasing population of HL survivors effectively.

Hodgkin lymphoma (HL) is a B-cell malignancy accounting for approximately 10% of all lymphomas worldwide [1,2]. It comprises two subtypes: classical HL (~90%) and nodular lymphocyte-predominant HL (~10%) [3,4]. Although five-year survival exceeds 85% in high-income countries [5,6], its pathogenesis remains incompletely understood [7]. Diagnosis relies on histopathological examination, with excisional lymph node biopsy as the gold standard [8,9].

Global annual incidence is approximately three per 100,000, with a bimodal age distribution affecting young (15–35 years) and older adults (>60 years) [10,11]. Risk factors include Epstein–Barr virus infection (20–40% in Western populations, up to 80% in developing countries) [5,12], HIV infection [6], and immune dysregulation [13]. While chemotherapy and radiotherapy achieved high cure rates, the past decade has seen brentuximab vedotin (an anti-CD30 antibody-drug conjugate) and PD-1 inhibitors (Nivolumab, Pembrolizumab) for relapsed/refractory disease [14], with ongoing integration into frontline therapy [15].

Recent Global Burden of Disease (GBD)-based studies have reported that epidemiological assessments of HL burden have limitations [16,17]. Many studies conflate HL with non-HL or lack granularity, potentially obscuring subtype-specific trends. Crucially, they often fail to adequately capture the influence of evolving demographic patterns (*e.g.* ageing populations, adolescent/young adult peaks), nuanced age-specific trends at national levels, and interactions with lifestyle or metabolic risk factors, all essential for developing evidence-based, targeted public health strategies.

Consequently, there is an urgent need for a contemporary, comprehensive re-evaluation of the GBD of HL that accounts for these dimensions. We aimed to employ age-period-cohort (APC) analysis and autoregressive integrated moving average (ARIMA) models to analyse incidence trends from 1990 to 2021, project prevalence to 2050, quantify transnational inequalities in the burden of HL, and utilise GBD 2021 data to inform public health strategies. By addressing these questions, we aim to provide evidence to guide diagnosis, treatment allocation, and long-term survivorship planning for HL.

## METHODS

We strictly followed the GRADROP and Gather guidelines to ensure research rigour (Table S1 in the **Online Supplementary Document**) [18].

### Data source

We extracted the HL burden data, including incidence, prevalence, mortality, and disability-adjusted life-years (DALYs) in complete form from the GBD Study 2021 results visualisation tools and databases [19]. The GBD 2021 provided comprehensive estimates for 204 countries and territories, across 20 age groups, for each year from 1990 to 2021. All estimates were presented with 95% uncertainty intervals (UIs). We did not perform any additional data cleaning, preprocessing, or imputation beyond the standardised GBD modelling framework, as the data set was complete and ready for analysis.

### Study period and sample size

As we used the complete GBD 2021 database, which includes estimates for all 204 countries and territories with no sampling, no formal sample size calculation was required. We selected the study period from 1990 to 2021 because it represents the full temporal scope of the latest GBD release, allowing for the longest possible trend analysis. This period captures three decades of epidemiological transition, including the introduction of novel immunotherapies in the last decade, enabling a comprehensive assessment of long-term trends. We chose the projection period to 2050 to provide a 30-year forward outlook, aligning with standard global health forecasting horizons.

### Statistical analysis

We calculated ASR per 100,000 persons using the GBD 2021 global standard population. To evaluate the burden of HL across regions and worldwide, we generated global maps and conducted regional comparative analyses. The estimated annual percentage change (EAPC) quantifies average ASR trends annually. The incidence, mortality, DALYs, and prevalence trends of HL were examined using the changes in HL cases from 1990 to 2021, the estimated EAPC, and corresponding 95% confidence intervals (CIs). We applied linear regression equations to evaluate the temporal trends of ASRs for HL burden at global, regional, and national levels [20]. A decomposition analysis disentangled contributions from three epidemiological drivers: population ageing, growth, and epidemiological changes. This approach allowed the independent assessment of each factor's contribution while keeping the other two constant, thereby determining the extent to which each specific factor impacted epidemiological trends.

We evaluated health disparities using the slope index of inequality (SII) for absolute gradients and the concentration index for relative inequalities. We derived the SII from weighted least squares regression, modelling the relationship between age-standardised mortality rates (ASMRs) and age-standardised DALY rates (ASDRs) and each country's socioeconomic rank (defined by the midpoint of its population in the cumulative sociodemographic index (SDI) distribution) [21,22]. The concentration index was calculated by fitting a Lorenz curve to the cumulative proportion of DALYs against the cumulative population ranked by SDI, with the index value equal to twice the area between this curve and the line of equality. We used the stochastic frontier regression to estimate the theoretical minimum achievable ASDR at each SDI level and identify countries deviating from expected burden levels given their development status (underperformers *vs.* outperformers).

To forecast the future burden of HL based on historical data, we employed an ARIMA model [23]. We also used an APC model to disentangle the effects of age, period, and cohort on health outcomes [24]. Specifically, the age effect reflects the risk of outcomes across different age groups; the period effect captures the influence of time-related changes on outcomes within various age groups; and the cohort effect represents the variation in outcomes among individuals born in the same period. All statistical analyses were performed using BioWinford [25,26], with a significance level set at *P* < 0.05. The platform is a specialised online tool for GBD data analysis, and we used validated modules built into the platform to ensure reproducibility.

## RESULTS

### Global prevalence trends

**Figure 1:**
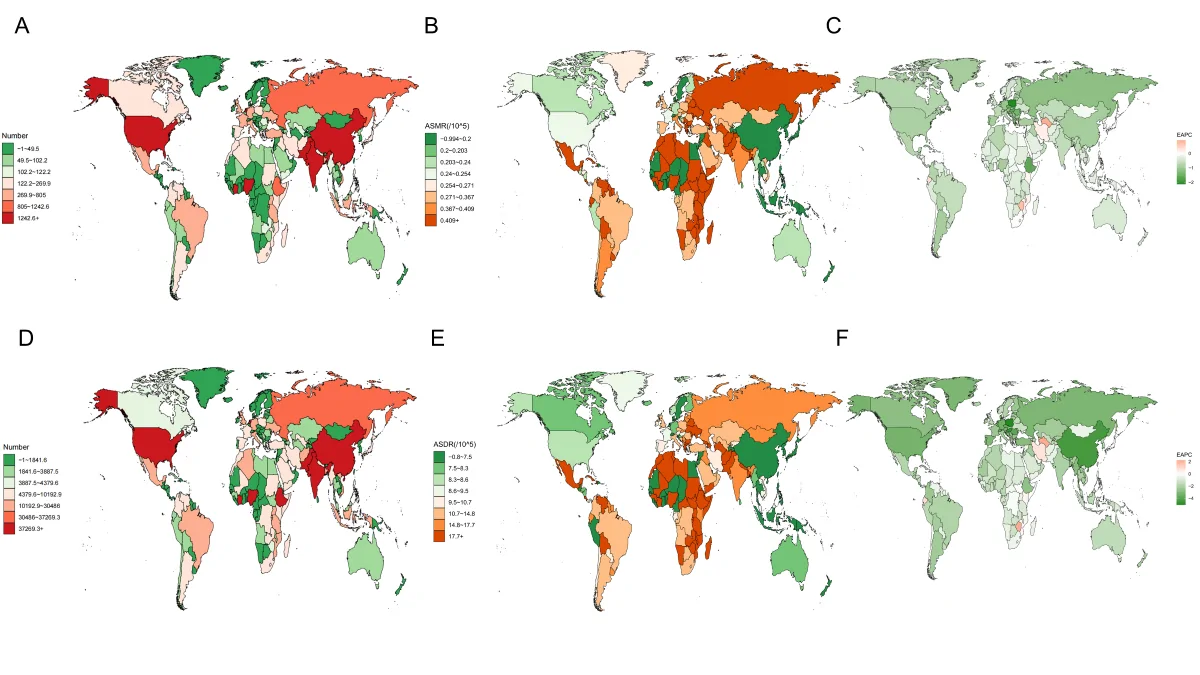
Global distribution of HL deaths and DALYs. **Panel A.** Death cases of HL. **Panel B.** ASMR of HL. **Panel C.** EAPC of ASMR. **Panel D.** DALYs of HL. **Panel E.** ASDR of HL. **Panel F.** EAPC of ASDR. ASDR – age-standardised disability-adjusted life year rate, ASMR – age-standardised mortality rate, DALY – disability-adjusted life-year, EAPC – estimated annual percentage change, HL – Hodgkin lymphoma.

Our analysis shows declining ASRs signify progress in HL control, yet rising absolute numbers pose healthcare system challenges due to demographic shifts. Globally, between 1990 and 2021, the prevalence, incidence, mortality, and DALYs of HL all declined to varying degrees. Although annual prevalence rose from 270,221 (95% UI = 241,909, 286,756) cases in 1990 to 361,579 (95% UI = 307,336, 414,718) in 2021, the average EAPC showed a yearly decrease of −0.48 (95% CI = −0.6, −0.36). Meanwhile, the global ASIR and ASMR steadily declined, and the ASPR showed a relatively modest decrease, from 5.24 (95% UI = 4.70, 5.55) in 1990 to 4.42 (95% UI = 3.75, 5.09) in 2021. Compared to females, males had higher case numbers and ASR for prevalence, incidence, DALYs, and mortality. Notably, the EAPC for male prevalence dropped by 0.38% yearly compared to the female decrease of 0.61%. Moreover, the EAPC for incidence, prevalence, DALYs, and mortality all gradually fell for both sexes (**Figure 1**, Panel A–F; Figure S1, Table S2–5 in the **Online Supplementary Document**).

In 2021, high SDI regions had the highest age-standardised prevalence rate (ASPR) of 10.67 per 100,000 (95% UI = 10.06, 11.31), followed by high-middle SDI (ASPR = 6.71; 95% UI = 6.02, 7.35). In contrast, low SDI regions had the lowest ASPR of 2.35 (95% UI = 1.38, 3.23). Notably, middle SDI regions experienced the largest relative increase in female HL prevalence of 59% from 1990 to 2021. High SDI regions demonstrated the most substantial declines in EAPC for incidence (EAPC = −1.18%; 95% CI = −1.41, −0.95) and mortality (EAPC = −3.37%; 95% CI = −3.46, −3.28), reflecting successful secondary prevention and treatment advances.

Age stratification revealed a characteristic bimodal distribution. In high SDI regions, males aged 20–29 years had the highest incidence, prevalence, mortality, and DALY rates (Figures S2 and S3 in the **Online Supplementary Document**). Among females, mortality peaks were observed at 25–29 and 70–74 years.

Significant variation existed in ASPR, ASMR, ASDR, and age-standardised incidence rate (ASIR) for HL across world regions (**Figure 1**, Panel A–F; Tables S1–4 in the **Online Supplementary Document**). Monaco had the highest ASPR of 72.94 per 100,000 (95% UI = 41.51, 123.08), followed by Hellenic Republic (ASPR = 40.79; 95% UI = 35.67, 47.10) and San Marino (ASPR = 38.93; 95% UI = 31.47, 47.82). In absolute terms, the USA had the largest prevalent population (n = 48,542; 95% UI = 46,304, 50,596), followed by India (n = 29,043; 95% UI = 19,547, 41,202) and the Russian Federation (n = 23,919; 95% UI = 21,886, 25,855). Notably, high ASPR in countries such as Monaco, Greece, and San Marino may reflect a combination of high diagnostic intensity, complete cancer registry coverage, and potential genuine variations in genetic or environmental risk factors, warranting further investigation.

In contrast, Nigeria had the highest ASMR of 1.56 per 100,000 (95% UI = 0.56, 2.44), followed by Pakistan (ASMR = 1.23; 95% UI = 0.84, 1.88) and Somalia (ASMR = 1.22; 95% UI = 0.73, 1.82), highlighting persistent mortality burden in low-resource settings. India recorded the highest absolute number of HL deaths (n = 4892; 95% UI = 3408, 7198), followed by China (n = 2443; 95% UI = 1,506, 3,231) and Nigeria (n = 2,382; 95% UI = 759, 3,870).

### The influential factor for EAPC

There were significant links between EAPC and ASPR, ASMR, ASIR, and ASDR (*P* < 0.05). Downward trends were observed for ASMR (*R*^2^ = 0.0059) and ASDR (*R*^2^ = 0.148). There was a nonlinear rise in EAPC (*P* < 0.00001, *R*^2^ = 1^−4^) when ASIR exceeded 2.5, and ASPR exceeded 40 (Figure S4 in the **Online Supplementary Document**).

### Age, period, and cohort effects on ASMR, ASDR, ASIR, ASPR

**Figure 2:**
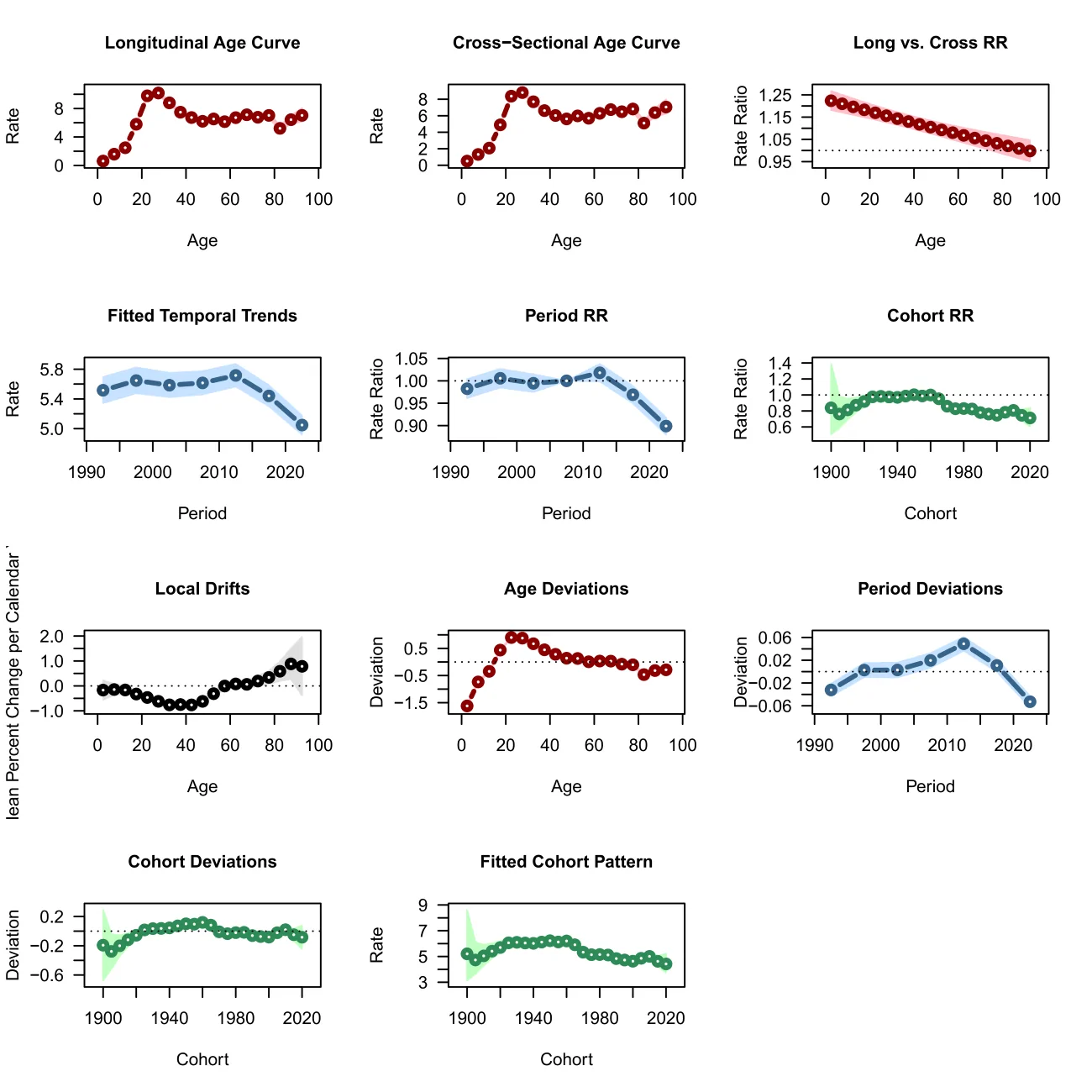
APC effect of ASR on the HL prevalence. ASR – age-standardised rate, APC – age-period-cohort, HL – Hodgkin lymphoma, RR – relative risk.

The highest mortality was in the 25–29 and 84–89 age groups (**Figure 2**; Figures S5–7 in **the** **Online Supplementary Document**). There was a steady decline in HL mortality across periods, with the relative risk falling from 3.52 in 1990 to 0.34 in 2022. For those aged 25–29, the relative risk for ASDR was 2.06, and 1.64 for ASIR and ASPR. Over time, ASDR, ASIR, and ASPR have all shown steady declines (Tables S6–9 in the **Online Supplementary Document**).

### Cross-country inequality analysis

**Figure 3:**
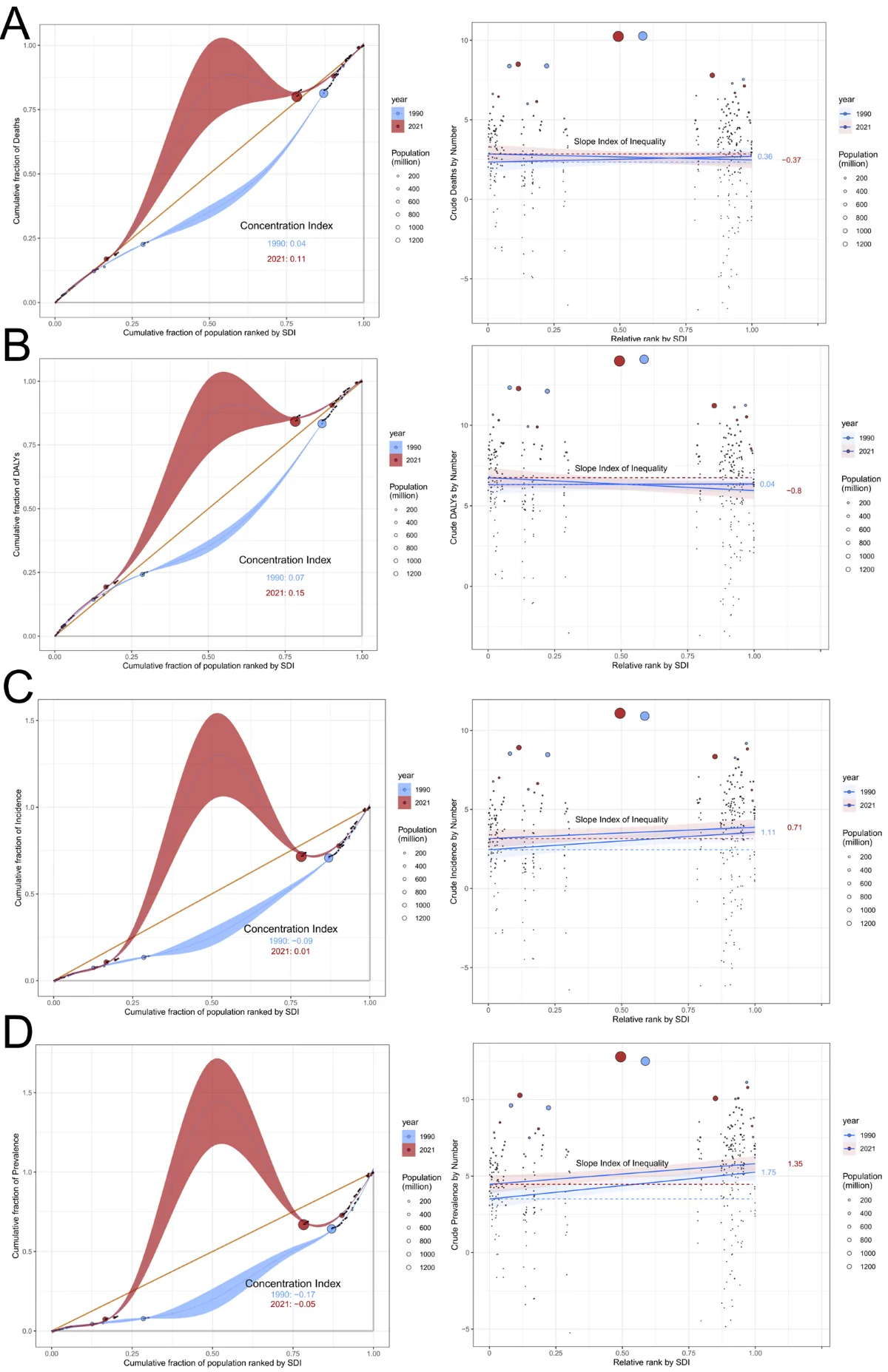
Cross-country social inequalities analysis based on SII and concentration index of HL. **Panel A.** ASMR. **Panel B.** ASDR. **Panel C.** ASIR. **Panel D.** ASPR. ASDR – age-standardised disability-adjusted life year rate, ASIR – age-standardised incidence rate, ASMR – age-standardised mortality rate, ASPR – age-standardised prevalence rate, DALY – disability-adjusted life-year, HL – Hodgkin lymphoma, SDI – sociodemographic index, SII – slope index of inequality.

From 1990 to 2021, HL incidence, prevalence, mortality, and DALYs generally decreased with increasing SDI across countries and regions. The 2021 SII was −0.37, significantly lower than the 1990 SII of 0.35 (**Figure 3**, Panel A–D; Figure S8, Table S9 in the **Online Supplementary Document**). For prevalence, the SII remained near zero throughout the study period, suggesting no systematic gradient by SDI. Overall, the declining SII suggested reducing inequality in the burden of HL DALYs, ASMR, and ASPR across countries and regions with varying SDI levels.

Concentration curves for both 1990 and 2021 deviated from the line of perfect equality (Figure S8 in the **Online Supplementary Document**). Critically, positive concentration indices of >0 in both observed years for mortality and DALYs indicated pro-rich inequalities-signifying (higher burden in affluent groups) disproportionate concentration of HL mortality and disability burden in higher socioeconomic groups (all *P* < 0.01 by Monte Carlo permutation test). Conversely, near-zero concentration indices for incidence and prevalence (range = −0.05, 0.01) demonstrated equitable distribution across socioeconomic strata. Notably, absolute burden metrics revealed incidence/prevalence rates persisting in middle and high-middle SDI regions despite distributional equity, highlighting residual disease clustering in developing economies undergoing epidemiological transition (Table S10 in the **Online Supplementary Document**).

### ARIMA model

As an exploratory analysis, we applied ARIMA models using the BioWinford platform (with default settings) to project HL burden to 2050 based on historical trends from 1990 to 2021. These projections assume the continuation of past patterns and do not account for potential future therapeutic breakthroughs. Under this assumption, the global ASPR rate is projected to increase by approximately 20% by 2050, from 2.92 in 2021 to 3.50 (95% UI = 2.98, 4.07) in 2050 (Figure S9 in the **Online Supplementary Document**), noting that this 2.92 baseline is derived from the BioWinford 'ALL' country grouping and differs from the 4.42 global ASPR reported in the main analysis due to differences in country coverage. The analysis projected ASMR and ASDR to continue their historical declines, while ASIR is projected to remain stable (Table S11 in the **Online Supplementary Document**).

Given the inherent uncertainty in long-term forecasting for a malignancy undergoing rapid therapeutic evolution, these projections should be viewed as scenario-based estimates rather than definitive predictions.

## DISCUSSION

We provided a comprehensive analysis of the global, regional, and national burden of HL from 1990 to 2021, with a unique emphasis on prevalence trends, their underlying APC effects, and associated transnational inequalities. Our key finding is that while global ASIR and ASMR have steadily declined, the ASPR of HL has remained remarkably stable (*e.g.* 5.24 (95% UI = 4.70, 5.55) in 1990; 4.42 (95% UI = 3.75, 5.09) in 2021). This highlights a growing population of HL survivors globally and increasing global adoption of novel immunotherapies after 2017 [24], with significant implications for long-term care. Furthermore, we found persistent and pronounced transnational inequalities, with low regions bearing a disproportionately higher mortality and DALY burden. The APC modelling elucidated distinct period effects driving overall declines and cohort effects influencing age-specific prevalence patterns, and ARIMA projections anticipate continued reductions in ASIR, ASMR, and DALYs but persistent disparities in ASPR to 2050.

We observed that the global rising ASPR, stable ASIR, and decline in ASDR and ASMR are key epidemiological changes, reflecting the success of modern HL therapy in improving survival rates [27]. This growing survivor population, also seen in other curable cancers, significantly alters public health priorities from acute management to long-term survivorship care, including managing late treatment effects and psychosocial support [28]. Our APC analysis further shows that period effects (reflecting advancements in diagnosis and treatment) largely drove declines in DALYs and mortality, while cohort effects (influenced by cumulative exposures or long-term health factors) notably shaped the stable incidence, particularly in older age groups.

Transnational inequity in HL burden remains a major public health concern. Our analysis, quantified by SII and concentration index values for ASPR, ASIR, ASMR and DALYs, demonstrates a disproportionate burden in lower SDI regions. For instance, low SDI regions continue to experience higher DALY rates (*e.g.* 30.28 per 100,000 in 2021), with Eastern Africa and the Federal Democratic Republic of Ethiopia showing a notable peak. In contrast, high SDI regions, despite achieving superior mortality reductions, often exhibit higher ASPR (*e.g.* 10.67 per 100,000). This suggests a complex interplay; advanced healthcare in high-SDI settings improves survival, while potential contributions to incidence from lifestyle factors, such as obesity [9,29–31] and environmental exposures [32,33], might play a role.

These profound inequalities in HL burden are likely mediated by several interconnected mechanisms. Diagnostic delays or misclassification remain common where access to complex diagnostic tools, such as excisional biopsy, immuno-histo-chemistry, or flow cytometry, is limited, often leading to late-stage presentation or incorrect subtyping. Even when a correct diagnosis is made, patients face substantial barriers to receiving basic multidrug chemotherapy, radiotherapy, or newer targeted agents, due to drug shortages, high out-of-pocket costs, and a lack of specialised treatment centres. Weak supportive care infrastructure, including inadequate infection control, inconsistent blood product availability, and limited intensive care capacity, further compromises treatment completion and survival. Compounding these health system gaps is a high burden of coinfections such as HIV, which not only increases HL incidence but also worsens prognosis through immune dysregulation and reduced tolerance to therapy. Addressing these vulnerabilities requires strategic investments in diagnostic infrastructure, ensuring equitable and affordable access to comprehensive treatment, building multidisciplinary oncology team capacity, and integrating essential supportive and survivorship care into existing healthcare frameworks.

Our cautious ARIMA projections to 2050 anticipate a continued decline in HL-related ASMRs and ASDRs, but with persistent regional and gender disparities in prevalence. These projections, as statistical extrapolations, are based on historical trends and current paradigms. They inherently carry significant uncertainty and do not fully account for unforeseen structural changes, such as future therapeutic breakthroughs (*e.g.* bispecific antibodies, CAR-T cells, checkpoint inhibitors) or major policy shifts, which could significantly alter these trajectories [6,11,15,34]. Therefore, these projections should serve as baseline scenarios, highlighting potential future demands rather than definitive predictions. The rising prevalence underscores the growing population of survivors, necessitating preparedness for long-term follow-up and management of chronic sequelae, including secondary malignancies and cardiovascular disease.

The implications for public health policy are clear and specific. The stable global prevalence demands a shift towards strengthening comprehensive survivorship programmes, especially in middle- and high-SDI regions. For low-SDI regions, the priority must be on fundamental health system strengthening to improve early diagnosis and ensure equitable access to comprehensive treatment and integrated supportive care. While targeted primary prevention efforts, such as risk assessment for obese adolescents or Epstein–Barr virus vaccination trials [9,31], are vital public health considerations, our GBD burden analysis primarily highlights the scale and distribution of the problem and the urgent need for broad improvements in access to care and survivorship support. Accurate diagnosis, requiring accessible pathology services and robust multidisciplinary team collaboration (including surgical input for biopsies and complication management), forms the foundation of HL management. By highlighting at-risk populations and regions, our data provides an evidence-based framework for optimising healthcare resources, improving training for diverse providers, and strengthening multidisciplinary team collaboration to enhance patient outcomes.

Our study has several limitations. First, GBD estimates depend on primary data quality, which varies across countries. In low-resource settings, incomplete cancer registries and limited diagnostic capacity (*e.g.* biopsy access) may lead to underdiagnosis or misclassification, and apparent ‘underperformance’ may partly reflect data sparsity. We did not perform any additional imputation; all estimates were obtained directly from GBD 2021, which uses DisMod-MR 2.1 and CODEm for sparse data. Second, our linear regression and EAPC analyses assume constant log-linear trends, which may not capture abrupt changes (*e.g.* post-2011 immunotherapy introduction). Third, findings are ecological; causal inferences at the individual level cannot be drawn. Fourth, individual-level data on risk factors (*e.g.* Epstein–Barr virus status, body mass index) were unavailable. Finally, projections to 2050 do not account for future therapeutic breakthroughs.

## CONCLUSIONS

Despite declining incidence and mortality, HL’s stable global prevalence reveals a growing survivor population, demanding enhanced survivorship care. Persistent transnational inequalities, particularly in low-SDI regions, highlight urgent needs for stronger health systems: improving diagnosis, equitable treatment access, and supportive care. Effective HL management requires global health equity, multidisciplinary collaboration, and robust survivorship programmes for all.

## Additional material


Online Supplementary Document

## Data Availability

**Data availability:** The data used can be found on the Global Health Data GBD 2021 website (https://ghdx.healthdata.org/gbd-2021).
