# Supplementary material for: Global prevalence trends and predictions of Hodgkin lymphoma from 1990 to 2021: age-period-cohort analysis and transnational inequality
Source: J Glob Health. 2026 Sep 11;16:04199. doi: 10.7189/jogh.16.04199 (PMC13564850; doi:10.7189/jogh.16.04199)
Supplement: Supplementary file 1 [file jogh-16-04199-s001.pdf]

Supplement to: Wang W, Li S, Ma D, Huang S, Kuang X, Tang Z, Fang Q, Wang J. Global prevalence trends and predictions of Hodgkin lymphoma from 1990 to 2021: age-period-cohort analysis and transnational inequality. J Glob Health. 2026;16:04199.

Supplements

Global prevalence trends and predictions of Hodgkin lymphoma from 1990 to 2021: age-period-cohort analysis and transnational inequality

Catalogue

Methods 2
Supplement Figure
Figure S1. EAPC in ASIR and ASPR of HL from 1990 to 2021. 3
Figure S2. The global incidence and prevalence rate, age and gender distribution of HL............................. 3
Figure S3. The global deaths and DALYs rate, age and gender distribution of HL............................. 4
Figure S4. The correlation between HL EAPC and ASMR, ASDR, ASIR, and ASPR. 4
Figure S5. Age–period–cohort effect of ASR of deaths for HL. 5
Figure S6. Age–period–cohort effect of ASR of DALYs for HL. 5
Figure S7. Age–period–cohort effect of ASR of incidence for HL. 6
Figure S8. The relationship between the unequal slope index of HL and years from 1990 to 2021. 6
Figure S9. ARIMA analysis predictions of HL by 2050. 7
Supplement Table
Table S1. Adherence to JoGH's GRABDROP guidelines items. 7
Table S2. The number of HL deaths and age standardized mortality of HL caused by different reasons from 1990 to 2021. 7
Table S3. The number of HL and age standardized DALYs rate of leukemia caused by different reasons from 1990 to 2021. 10
Table S4. The incident cases and age standardized incidence of HL caused by different reasons from 1990 to 2021. 14
Table S5. The prevalence cases and age standardized prevalence of HL caused by different reasons from 1990 to 2021. 16
Table S6. age, period, and cohort of deaths of HL. 19
Table S7. age, period, and cohort of DALYs of HL. 20
Table S8. age, period, and cohort of incidence of HL. 22
Table S9. age, period, and cohort of prevalence of HL. 23
Table S10. The trends in slope index of inequality and concentration index of HL crude incidence, prevalence, mortality and DALYs rates from 1990 to 2021. 24
Table S11. Predicted trends of HL deaths,incidence, prevalence, and DALYs in the next decade (2022-2050). 25

Additional materials Supplementary Methods

Section 1.1: Overview of GBD

The Institute for Health Metrics and Evaluation (IHME), an independent global health research center at the University of Washington, systematically quantifies and annually updates the global burden of diseases, injuries, and risk factors through the Global Burden of Disease (GBD) study. This initiative provides rigorously comparable measurements of the world's most critical health challenges. For GBD 2021, comprehensive datasets are publicly accessible via the Global Health Data Exchange (GHDx) query tool, enabling extraction and analysis of standardized epidemiological metrics.

Section 1.2: Data sources

The GBD study collected data from multiple relevant sources, including censuses, household surveys, vital registration and demographic statistics, disease registries, health service utilisation, air pollution monitors, satellite imaging, disease notifications, and other sources. In GBD 2021, all data were mapped to GBD disease categories based on the International Classification of Diseases (ICD) codes, 9th and 10th revisions. HL (HL) was defined according to ICD codes C81-C81.9.

Section 1.3: Estimates of fatal burden

Vital registration systems with medical certification of cause of death are crucial resources for estimating mortality burden. Data from vital registration and surveillance analyses were used to assess the fatal burden attributed to HL (HL) from the Cause of Death (COD) database. The Cause of Death Ensemble model (CODEm) is a highly systematic tool that employs a suite of modeling methods with different covariate selections to estimate mortality from HL, stratified by age, sex, location, and year. Disability-adjusted life-years (DALYs) represent the total number of years lost due to illness, disability, or premature death, calculated as the sum of years of life lost (YLLs) and years lived with disability (YLDs). The Disease Model Bayesian meta-regression (DisMod MR) 2.1 model was used to estimate the incidence and prevalence of HL by location, year, age, and sex, based on all available data.

Section 2: Statistical Analysis Plan of This Study

Section 2.1: Data Extraction

In this study, we extracted the incidence, mortality, disability-adjusted life-years (DALYs), and their age-standardised rates (per 100,000 population), as well as the attributable mortality and DALYs for risk factors of HL (HL), using the GHDx query tool and conducting a secondary analysis of the GBD 2021 data.

Section 2.2: Comparisons Across Groups

We analysed the global and regional incidence, prevalence, mortality, and DALYs for HL in 1990 and 2021 to obtain an overall impression of the HL burden. Subsequently, we used age-standardised incidence, prevalence, mortality, and DALYs to compare different groups by sex, age, year, and location. The GBD study utilised the Socio-demographic Index (SDI) as a composite indicator of the background social and economic conditions influencing health outcomes in each location. The SDI ranges from 0 (lowest) to 100 (highest) and is calculated based on the following indices: total fertility rate under the age of 25, mean years of education for individuals aged 15 years and older, and lagged per capita income distribution. A higher SDI indicates better socio-economic conditions. Countries and regions were further categorised into high, high-middle, middle, low-middle, or low SDI regions based on the quintiles of the 2021 SDI. Comparisons were made across 21 geographical regions and 204 countries and territories, stratified by different SDI regions. Differences were grouped by sex, age, year, and location and were visualised through tables and charts.

Section 2.3: Calculation of Estimated Annual Percentage Change

The percentage changes in the numbers and age-standardised rates of incidence, prevalence, mortality, and disability-adjusted life-years (DALYs) were calculated from 1990 to 2021. To further quantify the dynamic changes over time between 1990 and 2021, the estimated annual percentage change (EAPC) of the age-standardised rates (ASR) was calculated using the following methods (10, 11). The natural logarithm of the ASR was assumed to change linearly over time. Therefore, the EAPC was calculated based on the following formula:

$$Y=\alpha+\beta X+\epsilon$$

where Y represents ln(ASR), X represents the calendar year, α represents the intercept, β represents the annual change in ln(ASR), and ϵ represents the error term. The EAPC was then calculated using the formula:

$$EAPC=100\times(e\beta-1)$$

The 95% confidence intervals were obtained from the linear model. A positive EAPC indicates an increasing trend, while a negative value represents a decreasing trend. The associations between the Socio-demographic Index (SDI) and the age-standardised incidence, prevalence, mortality, and DALY rates were measured using Spearman's rank correlation coefficient. These relationships were visualised using locally weighted scatterplot smoothing (LOESS) curves. All statistical analyses were performed using the R software (version 4.4.2). A p-value of less than 0.05 was considered statistically significant.

Reference:

1. Salomon JA. Disability-Adjusted Life Years. In: Culyer AJ, editor. Encyclopedia of Health Economics. San Diego: Elsevier; 2014. p. 200-3.
2. Murray CJ. Quantifying the burden of disease: the technical basis for disability-adjusted life years. Bulletin of the World Health Organization. 1994;72(3):429-45.
3. Hankey BF, Ries LA, Kosary CL, Feuer EJ, Merrill RM, Clegg LX, et al. Partitioning linear trends in age-adjusted rates. Cancer causes & control : CCC. 2000;11(1):31-5.
4. Li N, Deng Y, Zhou L, Tian T, Yang S, Wu Y, et al. Global burden of breast cancer and attributable risk factors in 195 countries and territories, from 1990 to 2017: results from the Global Burden of Disease Study 2017. Journal of hematology & oncology. 2019;12(1):140.

Supplemnet Figure

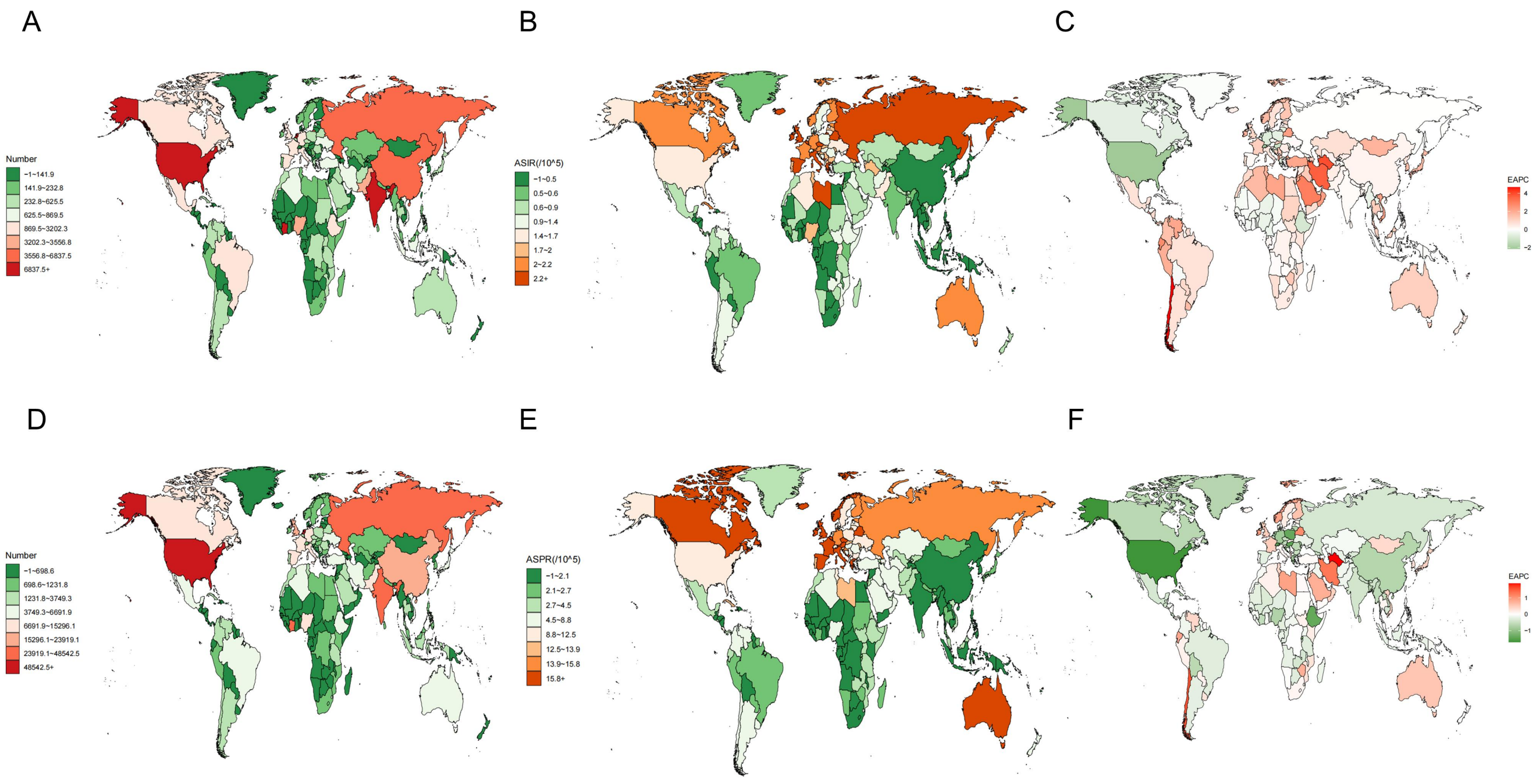

Figure S1. EAPC in ASIR and ASPR of HL from 1990 to 2021. **A-C.The cases, ASIR, and EAPC of HL; D-E: The cases, ASIR, and EAPC of HL. ASIR, age standardized incidence rate; ASPR, age standardized prevalence rate；EAPC, estimated annual percentage change.**

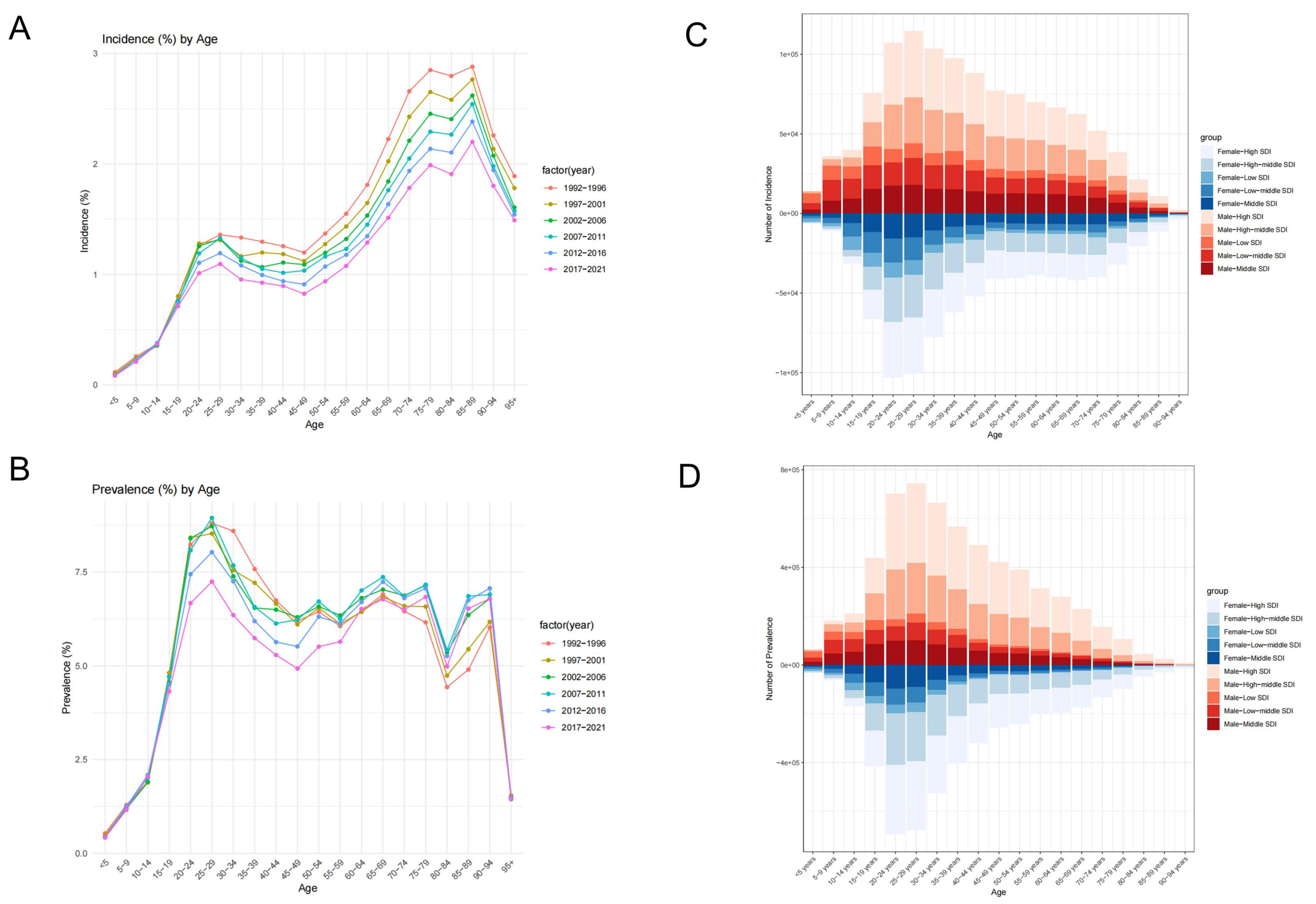

Figure S2. The global incidence and prevalence rate, age and gender distribution of Hodgkin's lymphoma. **A.C The incidence rate of HL. B.D The prevalence rate of HL.**

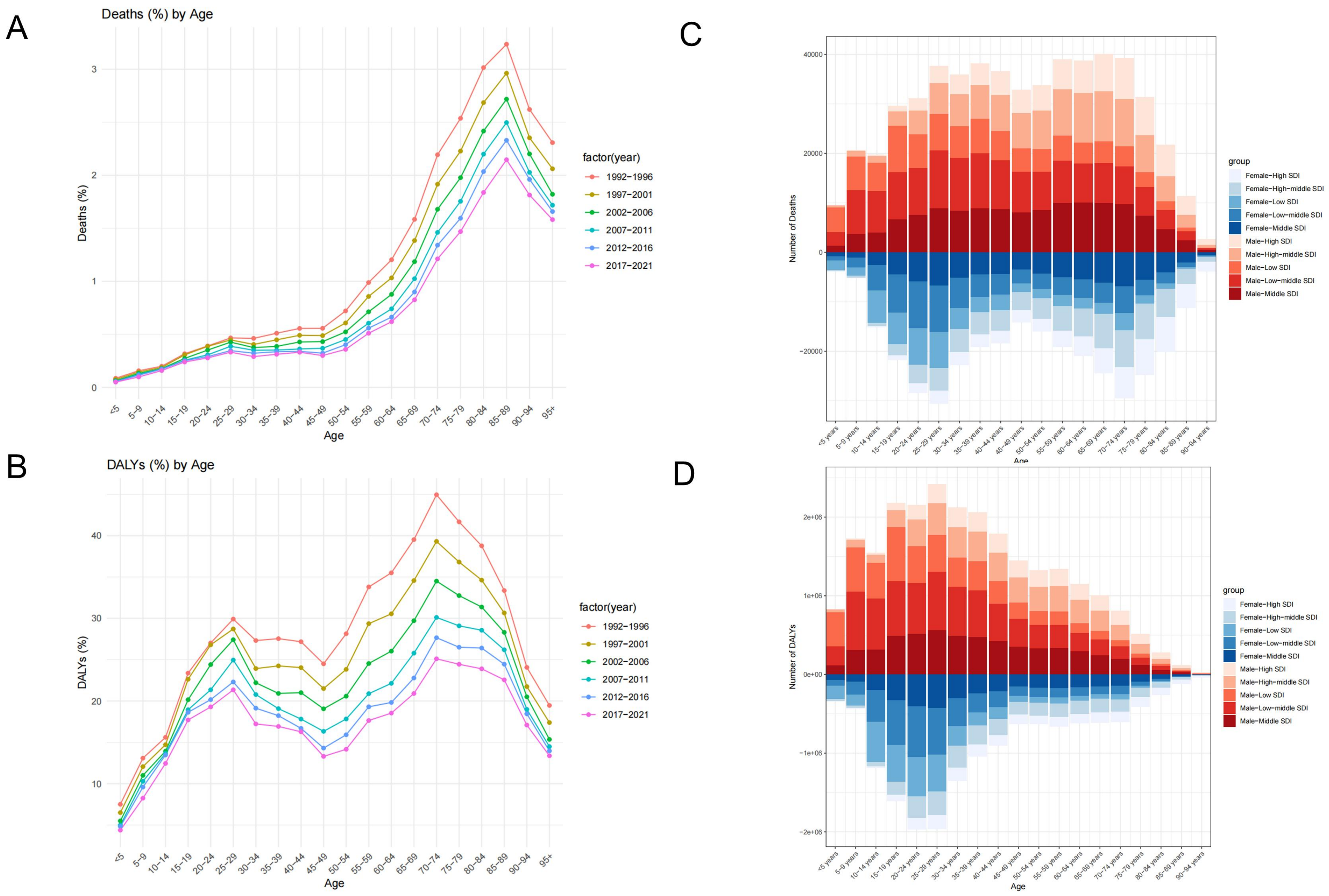

Figure S3. The global deaths and DALYs rate, age and gender distribution of Hodgkin's lymphoma. **A. C The death rate of HL. B. D The DALYs rate of HL.**

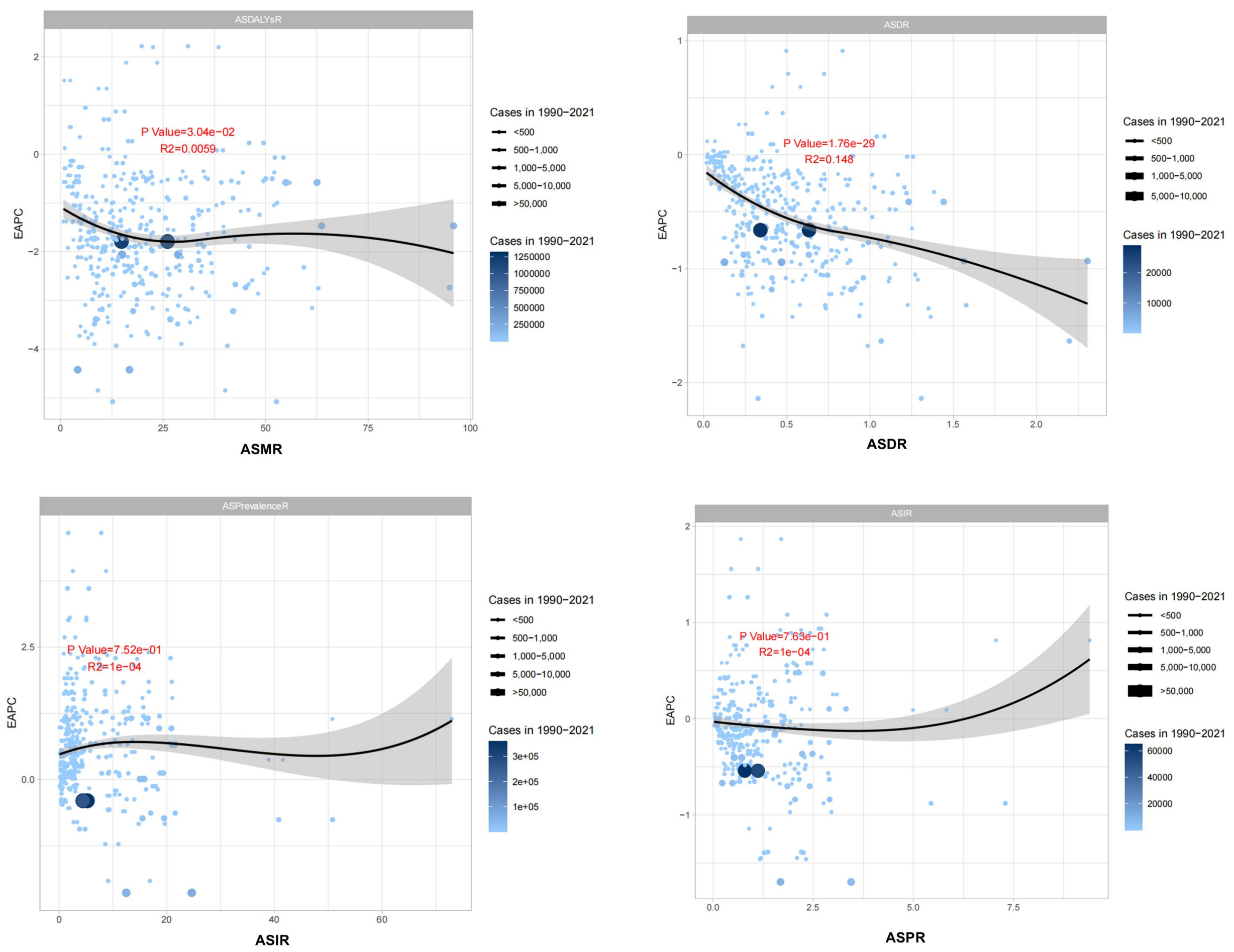

Figure S4. The correlation between HL EAPC and ASMR, ASDR, ASIR, and ASPR. **P<0.00001, ASMR, age standardized deaths rate; ASDR, age standardized DALYs rate; ASIR,age standardized incidence rate; ASPR, age standardized prevalence rate; EAPC, estimated annual percentage change.**

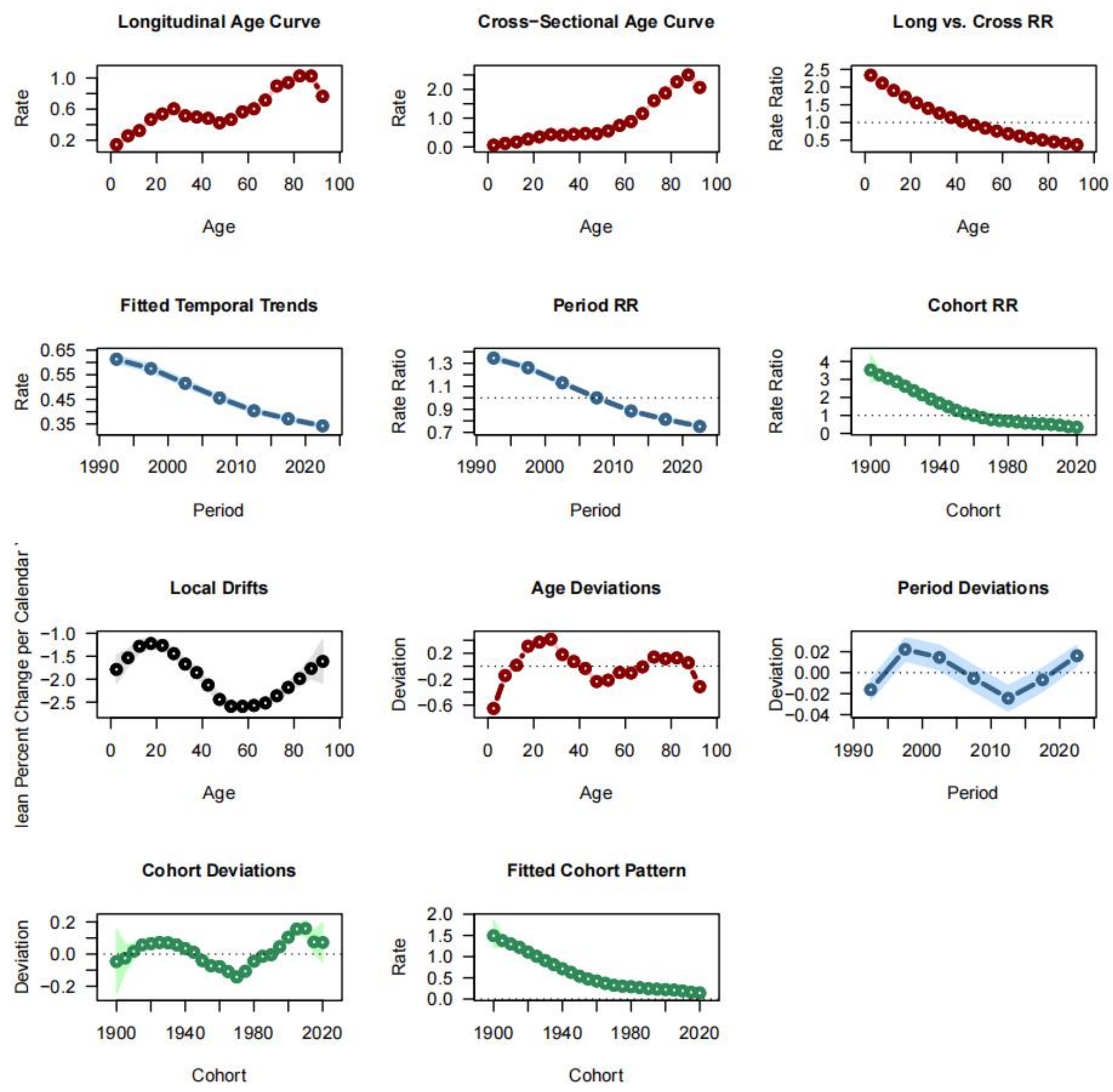

Figure S5. Age–period–cohort effect of ASR of deaths for HL.

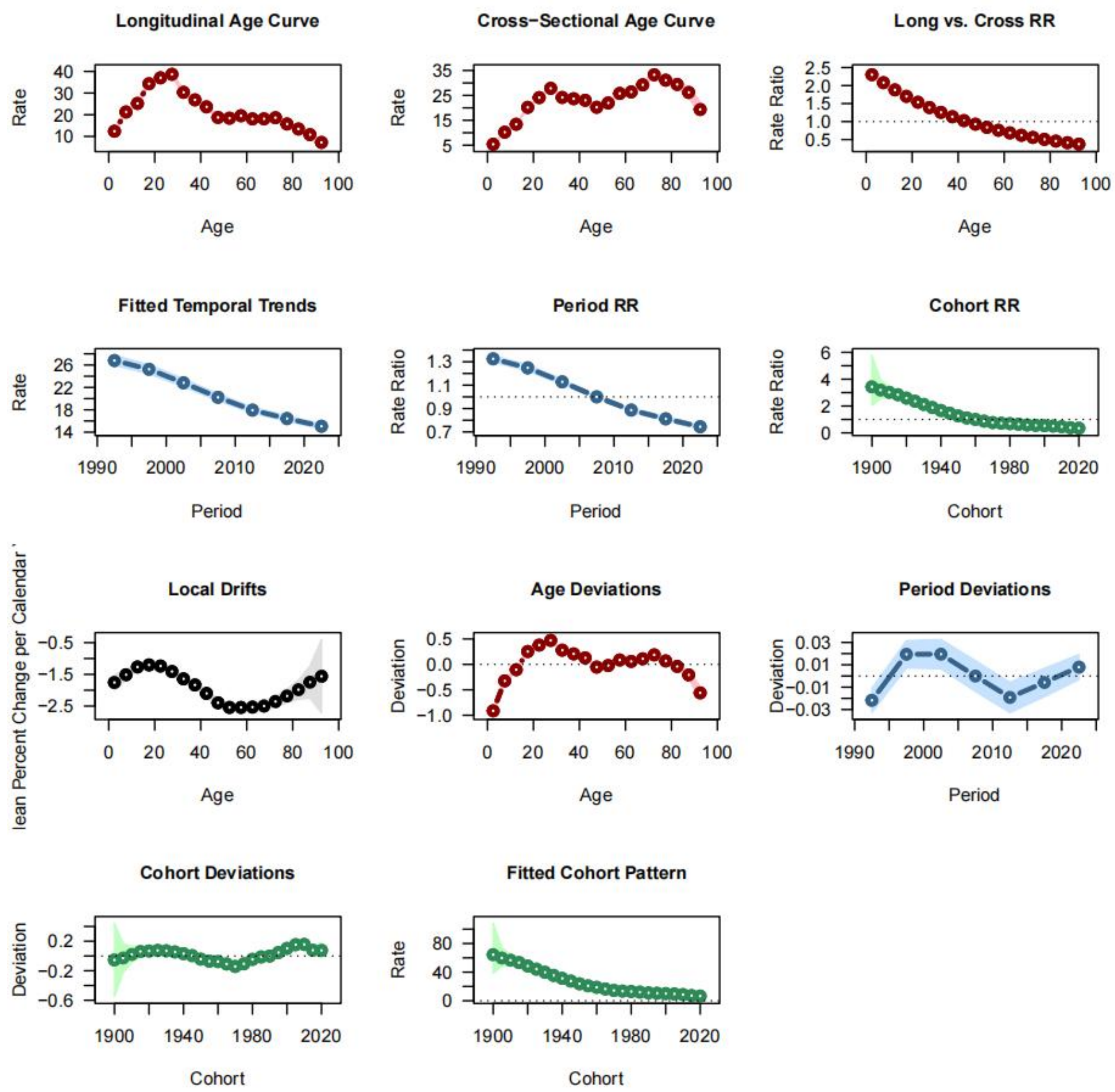

Figure S6. Age–period–cohort effect of ASR of DALYs for HL.

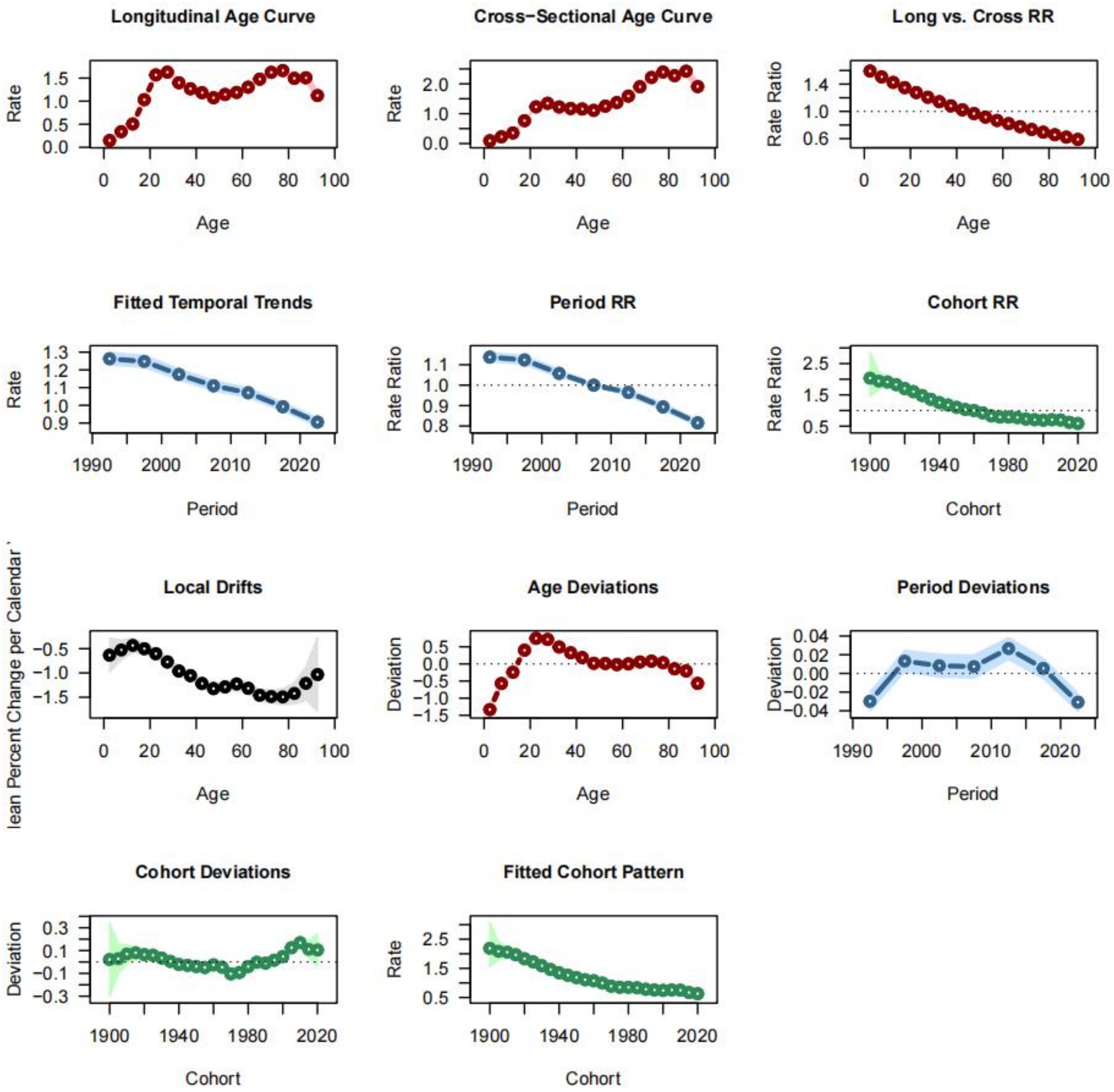

Figure S7.Age–period–cohort effect of ASR of incidence for HL.

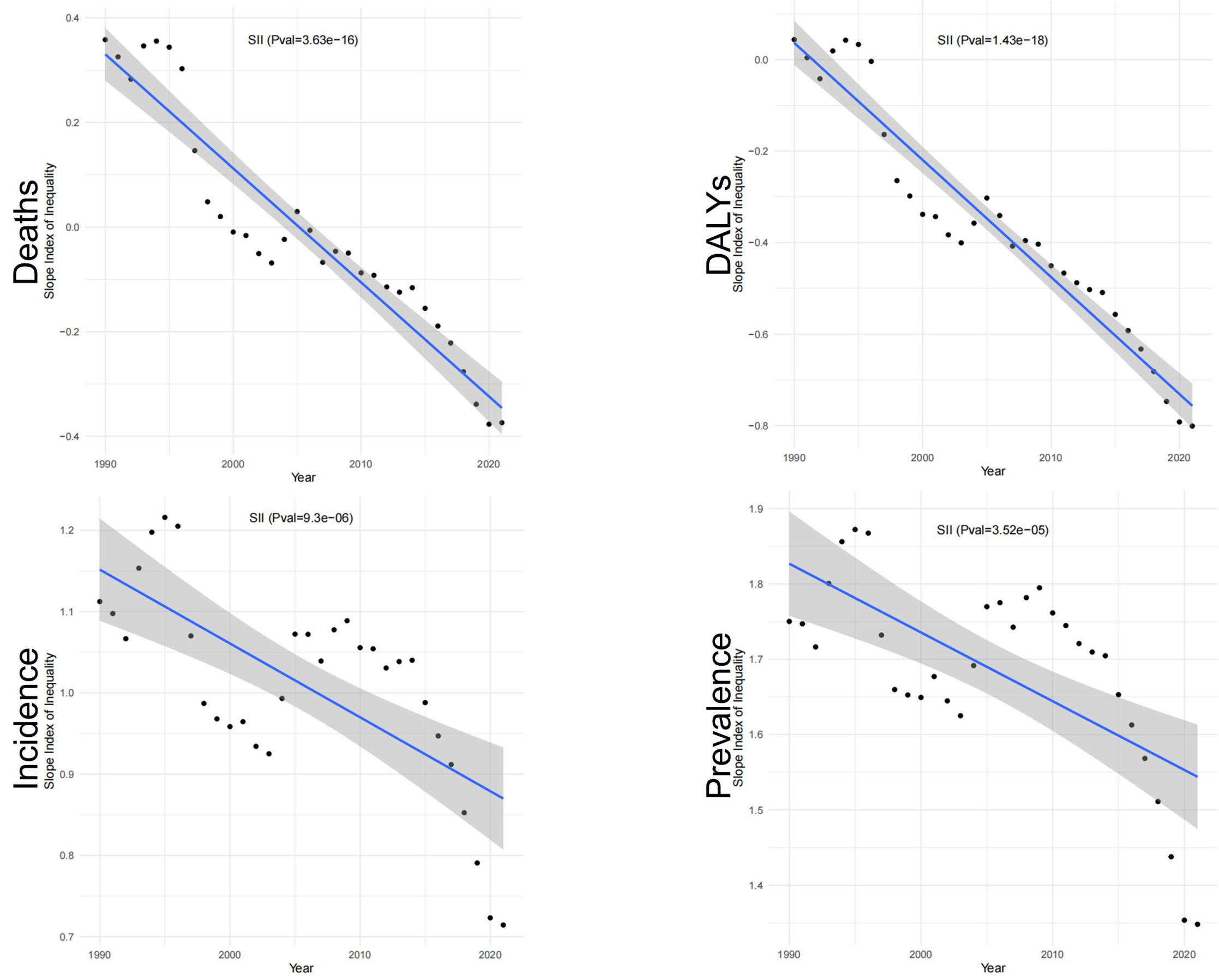

Figure S8. The relationship between the unequal slope index of HL and years from 1990 to 2021.

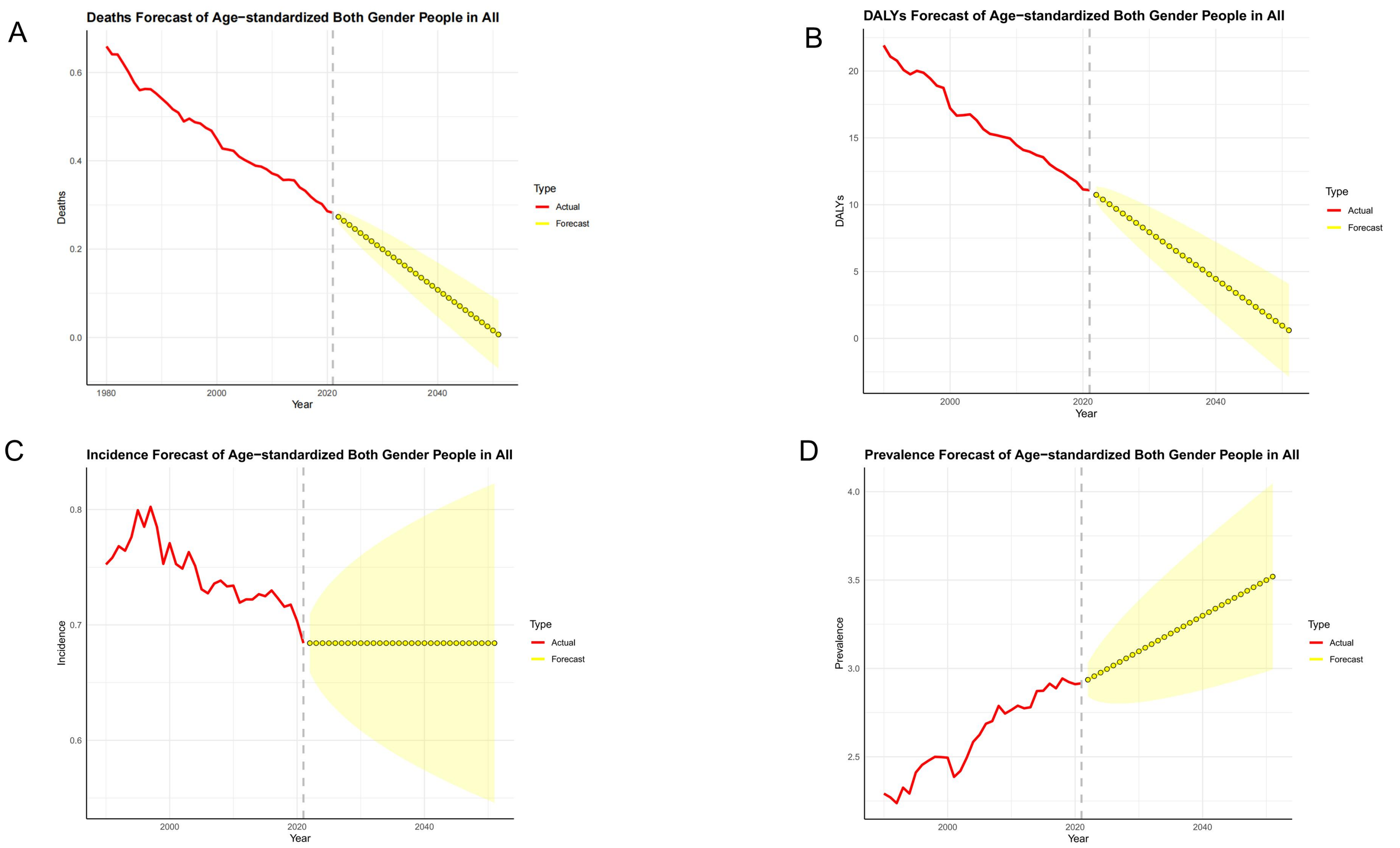

Figure S9. ARIMA analysis predictions of HL by 2050. **A. Global mortality. B. DALYs. C. Incidence rate. D. Prevalence rate. ARIMA – autoregressive integrated moving average, DALY – disability-adjusted life-year, HL – Hodgkin lymphoma. The 2021 baseline ASPR of 2.92 in this table is derived from the BioWinford default country subset ('ALL'), which differs in country coverage from the complete GBD 2021 'Global' dataset. The global ASPR of 4.42 reported in the main analysis is based on all 204 countries/territories.**

Supplemnet Table

Table S1. Adherence to JoGH's GRABDROP guidelines items.

**JoGH guideline items**
1. Please list all papers published by each co-author in previous 3 years that were based on secondary analysis of a big data repository
No other co-authors have published GBD-based secondary analyses in the past 3 years.
2. Please explain the key elements of your study design and the use of the available datasets that make your study an original scientific contribution
This study provides the first comprehensive analysis of the global burden of Hodgkin lymphoma using the most recent GBD 2021 data, extending previous assessments by incorporating projections to 2050, decomposition analysis of demographic drivers, and inequality metrics (SII, CI) across 204 countries. The originality lies in: (1) updating the epidemiological landscape to 2021; (2) quantifying contributions of population aging and growth to rising prevalence; and (3) identifying frontier outliers for policy prioritization. No prior study has integrated these dimensions using the GBD 2021 dataset.
3. Please list all publications that addressed similar research questions in the same dataset and indicate where you cited them in your paper
We identified prior studies using GBD data to assess lymphoma burden or Hodgkin lymphoma trends. These studies were cited in the Introduction and Discussion as supporting references. Specifically, previously published work addressing global lymphoma burden, GBD-based HL trends, and socioeconomic disparities was cited in the manuscript as References [5, 19]. The present study extends this literature by incorporating more recent GBD 2021 data and additional inequality and forecasting analyses.
4. Please explain how you addressed multiple testing through an appropriately rigorous statistical threshold and indicate this in the methods section
To address multiple comparisons across 204 countries, 21 regions, 4 indicators (incidence, prevalence, mortality, DALYs), and multiple time points, we applied a conservative statistical threshold: EAPC was considered significant only if the 95% confidence interval did not cross zero and the associated P-value was <0.05. This approach is clearly stated in the Methods section (Section 2.2).
5. Please declare to what extent have AI chatbots been used in developing your paper and to which parts of the paper did they contribute
AI tools were not used for data extraction, statistical analysis, interpretation of findings, or generation of scientific content. This time, ChatGPT was used for language polishing and grammar checking, and all final scientific decisions, analysis, and manuscript revisions were made by the author.

Table S2. The number of HL deaths and age standardized mortality of HL caused by different reasons from 1990 to 2021.

Cause	Location	1990		2021		EAPC No. (95% CI)
		Deaths cases (95% UI)	ASMR per 100,000 No. (95% UI)	Deaths cases (95% UI)	ASMR per 100,000 No. (95% UI)	
HL	American Samoa	0.02 (0.01-0.03)	0.06 (0.03-0.09)	0.03 (0.01-0.04)	0.06 (0.03-0.09)	0.61 (0.32-0.91)
HL	Antigua and Barbuda	0.1 (0.09-0.11)	0.17 (0.16-0.19)	0.09 (0.08-0.1)	0.09 (0.08-0.1)	-2.07 (-2.24--1.91)
HL	Arab Republic of Egypt	60.04 (27.8-156.19)	0.17 (0.08-0.46)	81.72 (40.7-194.53)	0.11 (0.05-0.27)	-0.83 (-1.2--0.46)
HL	Argentine Republic	283.59 (235.39-345.92)	0.88 (0.73-1.08)	194.07 (163.22-230.93)	0.36 (0.31-0.43)	-2.46 (-2.84--2.08)
HL	Australia	82.11 (72.75-91.84)	0.43 (0.38-0.48)	85.91 (69.95-103.73)	0.21 (0.17-0.26)	-1.76 (-2.02--1.51)
HL	Barbados	1.39 (1.23-1.55)	0.49 (0.44-0.54)	1.27 (0.98-1.6)	0.29 (0.23-0.37)	-1.8 (-1.98--1.62)
HL	Belize	0.7 (0.59-0.99)	0.54 (0.46-0.76)	1.45 (1.24-1.69)	0.39 (0.33-0.45)	-1.17 (-1.6--0.74)
HL	Bermuda	0.6 (0.51-0.71)	0.95 (0.8-1.12)	0.33 (0.26-0.42)	0.3 (0.24-0.38)	-3.82 (-4.04--3.61)
HL	Bolivarian Republic of Venezuela	77.68 (68.79-88.08)	0.63 (0.56-0.71)	154.08 (114.23-202.4)	0.54 (0.4-0.7)	-0.72 (-0.95--0.5)
HL	Bosnia and Herzegovina	32.22 (20.15-43.71)	0.72 (0.46-0.97)	16.38 (11.03-25.66)	0.31 (0.21-0.5)	-3.08 (-3.34--2.82)
HL	Brunei Darussalam	0.71 (0.49-1.3)	0.44 (0.31-0.81)	1.39 (0.9-1.9)	0.35 (0.22-0.47)	0.11 (-0.17-0.39)
HL	Burkina Faso	12.73 (6.5-17.58)	0.21 (0.11-0.29)	17.89 (11.58-27.66)	0.13 (0.08-0.19)	-1.75 (-1.95--1.55)
HL	Canada	176.95 (158.49-198.34)	0.56 (0.5-0.63)	122.17 (99.71-150.24)	0.2 (0.17-0.25)	-3.15 (-3.25--3.06)
HL	Central African Republic	9.36 (6.58-14.06)	0.57 (0.4-0.86)	17.19 (11.39-25.57)	0.5 (0.33-0.76)	-0.39 (-0.43--0.35)
HL	Commonwealth of Dominica	0.3 (0.18-0.44)	0.47 (0.29-0.7)	0.3 (0.19-0.45)	0.39 (0.25-0.58)	-0.6 (-0.72--0.49)
HL	Commonwealth of the Bahamas	1 (0.89-1.12)	0.47 (0.42-0.53)	1.25 (0.99-1.59)	0.3 (0.24-0.38)	-1.68 (-1.84--1.52)
HL	Cook Islands	0.01 (0.01-0.02)	0.07 (0.05-0.12)	0.01 (0-0.01)	0.04 (0.02-0.06)	-1.27 (-1.68--0.85)
HL	Czech Republic	144.11 (122.29-170.29)	1.13 (0.95-1.35)	57.37 (44.7-70.6)	0.32 (0.25-0.4)	-4.02 (-4.17--3.87)
HL	Democratic People's Republic of Korea	40.77 (23.81-60.39)	0.23 (0.14-0.33)	49.52 (34.25-83.49)	0.16 (0.11-0.26)	-1.3 (-1.34--1.25)
HL	Democratic Republic of Sao Tome and Principe	0.01 (0-0.01)	0.01 (0-0.01)	0.01 (0-0.01)	0.01 (0-0.01)	-0.76 (-0.94--0.57)
HL	Democratic Republic of the Congo	88.15 (59.05-140.6)	0.4 (0.26-0.64)	195.46 (121.17-309.92)	0.36 (0.22-0.57)	-0.21 (-0.27--0.15)
HL	Democratic Republic of Timor-Leste	1.51 (0.86-3)	0.32 (0.18-0.64)	2.16 (1.09-5.08)	0.2 (0.1-0.48)	-1.4 (-1.74--1.06)
HL	Democratic Socialist Republic of Sri Lanka	73.44 (42.27-95.19)	0.53 (0.3-0.69)	47.94 (29.44-82.55)	0.19 (0.12-0.33)	-3.78 (-4.05--3.51)
HL	Dominican Republic	3.76 (2.01-9.02)	0.07 (0.04-0.17)	5.48 (2.71-12.6)	0.05 (0.03-0.12)	-0.86 (-1.18--0.55)
HL	Eastern Republic of Uruguay	27.09 (22.53-31.86)	0.75 (0.63-0.88)	21.1 (17.47-25.4)	0.45 (0.37-0.54)	-1.72 (-2.09--1.35)
HL	Federal Democratic Republic of Ethiopia	737.25 (473.29-996.88)	2.2 (1.36-2.96)	814.24 (481.67-1290.36)	1.07 (0.62-1.62)	-2.69 (-2.81--2.56)
HL	Federal Democratic Republic of Nepal	117.85 (78.98-195.94)	0.82 (0.55-1.41)	120.93 (67.11-252.9)	0.44 (0.25-0.91)	-1.94 (-2.15--1.72)
HL	Federal Republic of Germany	834.15 (744.54-920.65)	0.75 (0.67-0.82)	392.18 (318.94-470.33)	0.23 (0.19-0.27)	-3.6 (-4.07--3.13)
HL	Federal Republic of Nigeria	1476.19 (520.89-2254.64)	2.31 (0.87-3.51)	2383.32 (759.79-3870.36)	1.56 (0.56-2.44)	-1.41 (-1.49--1.32)
HL	Federal Republic of Somalia	62.29 (37.33-88.04)	1.26 (0.76-1.8)	161.2 (93.14-249.44)	1.22 (0.73-1.82)	-0.03 (-0.1-0.04)
HL	Federated States of Micronesia	0.08 (0.04-0.11)	0.11 (0.06-0.16)	0.07 (0.04-0.1)	0.07 (0.04-0.11)	-1.3 (-1.37--1.23)
HL	Federative Republic of Brazil	648.67 (621.41-680.15)	0.57 (0.55-0.6)	650.86 (602.25-696.61)	0.27 (0.25-0.29)	-2.2 (-2.4--2.01)
HL	French Republic	419.93 (375.8-465.98)	0.58 (0.51-0.64)	279.12 (227.17-340.21)	0.24 (0.2-0.29)	-2.57 (-2.7--2.44)
HL	Gabonese Republic	2.61 (1.76-3.94)	0.38 (0.26-0.58)	3.53 (2.29-5.26)	0.27 (0.18-0.39)	-1.21 (-1.33--1.09)
HL	Georgia	66.86 (41.72-82.79)	1.12 (0.7-1.39)	40.87 (33.13-49.79)	0.84 (0.69-1.02)	-0.96 (-1.48--0.43)
HL	Global	28977.06 (21389.74-33106.92)	0.63 (0.47-0.72)	28179.99 (20895.4-35652.96)	0.34 (0.25-0.43)	-2.07 (-2.13--2)
HL	Grand Duchy of Luxembourg	2.69 (2.47-2.93)	0.54 (0.5-0.59)	1.62 (1.41-1.86)	0.17 (0.15-0.19)	-3.6 (-3.78--3.42)
HL	Greenland	0.32 (0.19-0.42)	0.65 (0.4-0.87)	0.17 (0.12-0.29)	0.25 (0.18-0.44)	-3.17 (-3.29--3.06)
HL	Grenada	0.37 (0.31-0.48)	0.5 (0.41-0.64)	0.27 (0.22-0.33)	0.24 (0.19-0.29)	-2.31 (-2.5--2.12)
HL	Guam	0.12 (0.08-0.18)	0.1 (0.07-0.16)	0.17 (0.08-0.21)	0.1 (0.05-0.13)	0.21 (-0.39-0.8)
HL	Hashemite Kingdom of Jordan	7.03 (4.9-11.39)	0.33 (0.23-0.53)	16.39 (10-23.62)	0.17 (0.11-0.25)	-2.01 (-2.21--1.8)
HL	Hellenic Republic	214.78 (201.36-227.87)	1.58 (1.48-1.67)	142.38 (125.9-156.92)	0.73 (0.66-0.8)	-2.52 (-2.93--2.11)
HL	High SDI	5618.56 (5385.3-5838.74)	0.54 (0.52-0.56)	3621.5 (3313.31-3860.61)	0.2 (0.18-0.21)	-3.12 (-3.21--3.03)
HL	High-middle SDI	6830.29 (5416.51-7795.2)	0.66 (0.52-0.75)	4472.94 (3735.3-4959.36)	0.25 (0.21-0.28)	-3.24 (-3.32--3.15)
HL	Hungary	127.03 (111.95-144.56)	0.99 (0.87-1.12)	37.64 (29.45-48.39)	0.24 (0.19-0.3)	-4.92 (-5.08--4.77)
HL	Independent State of Papua New Guinea	2.76 (1.04-4.74)	0.09 (0.04-0.16)	5.45 (2.75-8.45)	0.07 (0.03-0.11)	-1.01 (-1.06--0.96)
HL	Independent State of Samoa	0.99 (0.6-1.92)	0.85 (0.53-1.67)	1.22 (0.71-2.43)	0.71 (0.41-1.42)	-0.49 (-0.61--0.37)
HL	Ireland	31.15 (27.76-34.78)	0.8 (0.71-0.89)	19.23 (15.57-23.4)	0.27 (0.22-0.33)	-2.76 (-3.01--2.51)
HL	Islamic Republic of Afghanistan	91.5 (55.22-169.05)	1.21 (0.73-2.25)	189.56 (122.48-298.46)	1.03 (0.65-1.64)	-0.54 (-0.69--0.38)
HL	Islamic Republic of Iran	109.6 (81.89-185.15)	0.29 (0.21-0.51)	220.24 (112.76-271.8)	0.27 (0.14-0.33)	0.83 (0.37-1.28)
HL	Islamic Republic of Mauritania	2.62 (1.46-3.61)	0.18 (0.1-0.25)	2.71 (1.79-4.31)	0.09 (0.06-0.14)	-2.41 (-2.5--2.31)
HL	Islamic Republic of Pakistan	1180.28 (761.55-1790.44)	1.44 (0.93-2.31)	2314.59 (1579.47-3426.03)	1.23 (0.84-1.88)	-0.7 (-0.91--0.49)
HL	Jamaica	6.31 (5.42-7.35)	0.31 (0.27-0.37)	6.53 (4.81-8.78)	0.21 (0.16-0.28)	-1.32 (-1.71--0.93)
HL	Japan	143.04 (136.85-147.87)	0.09 (0.09-0.09)	235.99 (207.93-253.06)	0.07 (0.07-0.08)	-0.08 (-0.35-0.19)
HL	Kingdom of Bahrain	3.01 (1.81-4.19)	1.11 (0.68-1.46)	5.81 (3.53-8.75)	0.54 (0.32-0.74)	-2.53 (-2.76--2.3)
HL	Kingdom of Belgium	90.99 (79.42-104.25)	0.68 (0.59-0.77)	51.55 (42.11-62.9)	0.27 (0.22-0.33)	-2.87 (-3.03--2.7)
HL	Kingdom of Bhutan	3.47 (1.98-5.96)	0.8 (0.47-1.44)	2.99 (1.54-6.6)	0.42 (0.22-0.92)	-2.25 (-2.32--2.18)
HL	Kingdom of Cambodia	30.37 (20.19-55.99)	0.46 (0.3-0.84)	35.59 (18.86-77.35)	0.25 (0.13-0.53)	-2.08 (-2.18--1.98)
HL	Kingdom of Denmark	35.07 (30.69-40.36)	0.49 (0.43-0.56)	18.5 (14.76-22.53)	0.17 (0.14-0.21)	-3.12 (-3.44--2.8)
HL	Kingdom of Eswatini	2.33 (1.53-3.86)	0.5 (0.34-0.83)	4.36 (2.67-6.82)	0.51 (0.33-0.78)	0.37 (-0.09-0.83)
HL	Kingdom of Lesotho	4.31 (2.6-7.53)	0.41 (0.25-0.72)	8.4 (5.39-13.58)	0.58 (0.38-0.92)	1.78 (1.39-2.17)
HL	Kingdom of Morocco	150.09 (96.41-204.95)	0.77 (0.5-1.1)	185.31 (119.84-268.99)	0.52 (0.34-0.75)	-1.19 (-1.27--1.11)
HL	Kingdom of Norway	25.39 (23.61-27.01)	0.41 (0.39-0.44)	23.61 (20.77-26.29)	0.25 (0.23-0.28)	-1.36 (-1.76--0.96)
HL	Kingdom of Saudi Arabia	42.8 (13.9-70.91)	0.44 (0.15-0.7)	90.02 (22.07-157.1)	0.28 (0.08-0.47)	-1.13 (-1.3--0.96)
HL	Kingdom of Spain	350.87 (311.52-392.38)	0.74 (0.65-0.83)	224.68 (183.07-270.45)	0.27 (0.22-0.32)	-3.07 (-3.26--2.88)
HL	Kingdom of Sweden	39.52 (34.68-44.59)	0.3 (0.26-0.34)	30.85 (25.49-36.41)	0.15 (0.13-0.18)	-2.27 (-2.59--1.96)
HL	Kingdom of Thailand	83.03 (55.93-155.93)	0.17 (0.12-0.34)	84.04 (51.04-180)	0.1 (0.06-0.2)	-2.55 (-2.82--2.29)
HL	Kingdom of the Netherlands	110.07 (100.16-122.02)	0.59 (0.53-0.65)	85.51 (72.02-100.21)	0.28 (0.24-0.33)	-2.05 (-2.17--1.93)
HL	Kingdom of Tonga	0.04 (0.02-0.06)	0.06 (0.03-0.09)	0.04 (0.02-0.06)	0.04 (0.02-0.07)	-0.71 (-0.77--0.65)
HL	Kyrgyz Republic	28.49 (22.23-35.9)	0.79 (0.62-0.98)	13.98 (10.4-18.05)	0.23 (0.18-0.3)	-1.97 (-2.92--1.02)
HL	Lao People's Democratic Republic	13.37 (8.66-24.43)	0.47 (0.31-0.88)	13.23 (6.99-30.13)	0.22 (0.12-0.5)	-2.57 (-2.64--2.51)
HL	Lebanese Republic	31.08 (18.18-42.06)	1.29 (0.76-1.73)	36.51 (22.01-48.78)	0.6 (0.36-0.79)	-2.35 (-2.47--2.24)
HL	Low SDI	3757.08 (2190.65-4734.98)	1.06 (0.62-1.37)	5711.99 (3273.92-7873.57)	0.7 (0.41-0.96)	-1.5 (-1.57--1.43)
HL	Low-middle SDI	5982.12 (3904.89-8098.86)	0.69 (0.45-0.95)	7699.7 (5517.2-11204.7)	0.45 (0.33-0.66)	-1.42 (-1.47--1.37)

Cause	Location	1990		2021		EAPC No. (95% CI)
		Deaths cases (95% UI)	ASMR per 100,000 No. (95% UI)	Deaths cases (95% UI)	ASMR per 100,000 No. (95% UI)	
HL	Malaysia	48.6 (25.78-65.22)	0.38 (0.21-0.52)	72.35 (42.38-90.85)	0.24 (0.14-0.3)	-1.79 (-1.96--1.62)
HL	Middle SDI	6749.99 (4350.91-8031.42)	0.52 (0.33-0.62)	6648.34 (4795.92-8225.68)	0.26 (0.18-0.32)	-2.35 (-2.45--2.25)
HL	Mongolia	6.3 (3.44-12.83)	0.4 (0.22-0.84)	10.31 (6.21-14.94)	0.35 (0.21-0.5)	-0.4 (-0.78--0.01)
HL	Montenegro	5.44 (3.39-7.68)	0.85 (0.53-1.2)	4.86 (3.27-6.89)	0.57 (0.39-0.81)	-1.47 (-1.75--1.2)
HL	New Zealand	14.73 (12.5-17.34)	0.38 (0.32-0.44)	14.2 (11.84-16.77)	0.18 (0.15-0.22)	-2.26 (-2.97--1.55)
HL	North Macedonia	23.98 (14.27-34.32)	1.23 (0.73-1.75)	17.26 (11.71-27.25)	0.58 (0.39-0.91)	-2.76 (-2.95--2.57)
HL	Northern Mariana Islands	0.01 (0-0.01)	0.02 (0.01-0.04)	0.01 (0.01-0.02)	0.02 (0.01-0.03)	0.21 (-0.04-0.46)
HL	Palestine	12.96 (7.25-17.98)	1.11 (0.6-1.56)	21.06 (11.42-27.21)	0.65 (0.35-0.84)	-1.57 (-1.69--1.46)
HL	People's Democratic Republic of Algeria	198.08 (121.55-268.28)	1.12 (0.71-1.64)	239.43 (157.86-360.45)	0.62 (0.41-0.95)	-1.72 (-1.78--1.66)
HL	People's Republic of Bangladesh	760.31 (518.49-1241.01)	0.96 (0.65-1.63)	636.71 (355.05-1328.77)	0.41 (0.23-0.85)	-2.8 (-2.89--2.7)
HL	People's Republic of China	4392.92 (1869.39-6057.68)	0.47 (0.2-0.64)	2443.23 (1506.76-3231.64)	0.13 (0.08-0.17)	-4.69 (-4.92--4.47)
HL	Plurinational State of Bolivia	41.27 (24.38-55.43)	0.96 (0.59-1.26)	53.6 (30.85-81)	0.54 (0.31-0.8)	-1.89 (-1.96--1.82)
HL	Portuguese Republic	82.23 (73.7-90.88)	0.67 (0.61-0.75)	45.48 (37.16-55.16)	0.23 (0.19-0.28)	-3.46 (-3.61--3.32)
HL	Principality of Andorra	0.23 (0.14-0.34)	0.39 (0.22-0.57)	0.26 (0.15-0.4)	0.2 (0.12-0.3)	-1.84 (-2--1.68)
HL	Principality of Monaco	0.64 (0.33-0.91)	1.25 (0.65-1.75)	0.58 (0.35-0.95)	0.86 (0.52-1.4)	-1.27 (-1.32--1.21)
HL	Puerto Rico	33.12 (28.51-38.55)	0.91 (0.78-1.06)	18.72 (14.81-23.24)	0.36 (0.28-0.44)	-3.1 (-3.26--2.93)
HL	Republic of Albania	19.08 (12.19-28.5)	0.72 (0.46-1.11)	10.97 (7.26-17.86)	0.3 (0.2-0.48)	-2.94 (-3.13--2.75)
HL	Republic of Angola	27.34 (18.84-41.51)	0.45 (0.3-0.71)	60.03 (37.72-92.41)	0.32 (0.2-0.5)	-1.08 (-1.14--1.02)
HL	Republic of Armenia	12.23 (9.98-14.29)	0.39 (0.32-0.46)	8.87 (7.3-10.52)	0.23 (0.19-0.27)	-1.75 (-2.04--1.46)
HL	Republic of Austria	90.31 (81.14-100.23)	0.84 (0.76-0.93)	41.52 (35.07-49.11)	0.27 (0.23-0.31)	-3.5 (-3.87--3.13)
HL	Republic of Azerbaijan	48.71 (30.57-73.03)	0.75 (0.47-1.15)	52.2 (31.77-79.11)	0.48 (0.29-0.71)	-1.67 (-1.92--1.42)
HL	Republic of Belarus	91.89 (77.49-109.92)	0.77 (0.65-0.93)	61.9 (47.79-81.3)	0.48 (0.37-0.63)	-1.77 (-2.04--1.5)
HL	Republic of Benin	5.89 (3.12-8.1)	0.19 (0.11-0.26)	9.2 (5.78-14.32)	0.11 (0.07-0.17)	-1.95 (-2.13--1.78)
HL	Republic of Botswana	4.06 (2.47-6.97)	0.49 (0.31-0.82)	6.41 (3.62-10.73)	0.32 (0.2-0.52)	-1.33 (-1.52--1.14)
HL	Republic of Bulgaria	116.81 (96.55-139.49)	1.12 (0.93-1.33)	53.41 (41.22-69.83)	0.49 (0.38-0.63)	-2.57 (-2.71--2.43)
HL	Republic of Burundi	46.84 (27.36-64.85)	1.15 (0.69-1.58)	82.68 (42.32-128.61)	0.9 (0.47-1.39)	-0.94 (-1.01--0.87)
HL	Republic of Cabo Verde	0.05 (0.01-0.07)	0.02 (0.01-0.03)	0.04 (0.02-0.06)	0.01 (0-0.01)	-2.18 (-2.53--1.83)
HL	Republic of Cameroon	13.33 (6.92-18.35)	0.2 (0.11-0.26)	24.24 (14.74-38.12)	0.12 (0.07-0.18)	-1.85 (-2.05--1.65)
HL	Republic of Chad	7.57 (4.08-10.69)	0.19 (0.1-0.26)	15.32 (10.03-24.65)	0.16 (0.1-0.23)	-0.81 (-1.04--0.57)
HL	Republic of Chile	36.01 (30.85-42.7)	0.32 (0.27-0.38)	64.24 (54.06-75.5)	0.27 (0.23-0.32)	-0.01 (-0.75-0.74)
HL	Republic of Colombia	160.72 (145.81-176.29)	0.67 (0.6-0.73)	190.42 (153.72-234.56)	0.35 (0.29-0.43)	-2.41 (-2.62--2.21)
HL	Republic of Costa Rica	26.65 (23.49-29.91)	1.15 (1.02-1.29)	31.17 (25.35-37.72)	0.58 (0.47-0.7)	-2.19 (-2.43--1.95)
HL	Republic of C 么 te d'Ivoire	41.18 (24.67-61.25)	0.53 (0.35-0.84)	78.68 (47.79-121.84)	0.4 (0.27-0.64)	-0.79 (-0.92--0.67)
HL	Republic of Croatia	43.13 (38.2-48.56)	0.75 (0.67-0.84)	15.24 (12.64-18.25)	0.21 (0.17-0.25)	-4.16 (-4.44--3.87)
HL	Republic of Cuba	140.2 (114.39-164.64)	1.27 (1.04-1.49)	117.22 (97.63-143.28)	0.7 (0.58-0.86)	-2.13 (-2.29--1.98)
HL	Republic of Cyprus	6.09 (4.1-9.18)	0.83 (0.55-1.22)	5.49 (3.67-8.1)	0.29 (0.19-0.42)	-3.58 (-3.71--3.45)
HL	Republic of Djibouti	2.27 (1.26-3.53)	0.81 (0.46-1.24)	7.08 (3.96-11.49)	0.72 (0.42-1.14)	-0.37 (-0.62--0.13)
HL	Republic of Ecuador	25.48 (21.4-30.14)	0.38 (0.32-0.45)	80.04 (61.97-102.1)	0.48 (0.37-0.61)	1.24 (0.76-1.73)
HL	Republic of El Salvador	20.61 (13.29-29.05)	0.55 (0.35-0.79)	21.81 (13.98-29.61)	0.34 (0.22-0.47)	-1.5 (-1.69--1.31)
HL	Republic of Equatorial Guinea	1.28 (0.85-2.04)	0.5 (0.32-0.79)	2.1 (1.23-3.43)	0.23 (0.14-0.37)	-2.77 (-3.06--2.48)
HL	Republic of Estonia	14.15 (11.33-17.41)	0.77 (0.61-0.95)	5.39 (4.14-6.72)	0.27 (0.2-0.34)	-3.66 (-3.94--3.38)
HL	Republic of Fiji	1.23 (0.71-2.71)	0.21 (0.12-0.47)	2.16 (1.3-3.35)	0.25 (0.15-0.38)	1.39 (0.94-1.84)
HL	Republic of Finland	36.83 (33.22-41.36)	0.57 (0.51-0.64)	20.05 (16.7-23.3)	0.2 (0.17-0.23)	-2.84 (-3.12--2.57)
HL	Republic of Ghana	3.23 (1.03-5.27)	0.03 (0.01-0.06)	4.07 (2.02-5.96)	0.02 (0.01-0.02)	-2.69 (-3.03--2.36)
HL	Republic of Guatemala	22.18 (18.6-24.71)	0.45 (0.4-0.49)	25.78 (21.67-30.06)	0.2 (0.17-0.24)	-2.57 (-2.74--2.4)
HL	Republic of Guinea	27.04 (17.43-39.57)	0.58 (0.4-0.95)	46.42 (29.49-71.79)	0.49 (0.33-0.77)	-0.47 (-0.54--0.4)
HL	Republic of Guinea-Bissau	1.97 (1.05-2.88)	0.3 (0.16-0.42)	2.42 (1.62-3.72)	0.19 (0.13-0.28)	-1.64 (-1.75--1.52)
HL	Republic of Guyana	3.07 (2.55-3.76)	0.55 (0.48-0.66)	2.96 (2.26-3.81)	0.41 (0.31-0.52)	-0.75 (-0.97--0.53)
HL	Republic of Haiti	38.92 (25.55-57.92)	0.88 (0.56-1.36)	72.28 (38.52-111.66)	0.73 (0.38-1.12)	-0.48 (-0.56--0.41)
HL	Republic of Honduras	9.13 (5.83-12.18)	0.3 (0.19-0.41)	18.22 (10.86-26.86)	0.25 (0.15-0.37)	-0.58 (-0.68--0.49)
HL	Republic of Iceland	1.21 (1.07-1.4)	0.44 (0.38-0.5)	0.89 (0.72-1.08)	0.18 (0.14-0.22)	-2.29 (-2.55--2.03)
HL	Republic of India	4350.45 (2532.5-5459.65)	0.65 (0.37-0.82)	4892.7 (3408.12-7198.89)	0.37 (0.26-0.54)	-1.91 (-1.99--1.84)
HL	Republic of Indonesia	405.2 (278.77-758.9)	0.29 (0.2-0.55)	467.7 (274.38-1005.67)	0.18 (0.11-0.39)	-1.51 (-1.55--1.47)
HL	Republic of Iraq	93.29 (45.7-131.8)	0.77 (0.37-1.08)	132.82 (65.98-193.38)	0.43 (0.22-0.61)	-2.21 (-2.4--2.02)
HL	Republic of Italy	630.82 (596-665.74)	0.83 (0.78-0.88)	453.48 (403.62-499.21)	0.37 (0.34-0.41)	-2.08 (-2.39--1.76)
HL	Republic of Kazakhstan	68.37 (55.55-97.44)	0.45 (0.37-0.64)	51.52 (42.92-61.27)	0.27 (0.23-0.32)	-2.21 (-2.79--1.63)
HL	Republic of Kenya	61.05 (36.77-90.38)	0.42 (0.25-0.63)	143.96 (83.27-215.69)	0.4 (0.24-0.59)	0.3 (0.08-0.52)
HL	Republic of Kiribati	0.03 (0.01-0.05)	0.06 (0.02-0.08)	0.05 (0.02-0.08)	0.05 (0.02-0.08)	-0.34 (-0.4--0.27)
HL	Republic of Korea	44.41 (32.35-77.64)	0.13 (0.09-0.22)	42.41 (23.8-58.86)	0.05 (0.03-0.07)	-2.51 (-2.67--2.34)
HL	Republic of Latvia	21.83 (18.11-26.03)	0.69 (0.57-0.83)	10.54 (7.94-13.58)	0.37 (0.28-0.48)	-2.34 (-2.55--2.12)
HL	Republic of Liberia	3.22 (1.62-4.58)	0.19 (0.1-0.27)	4.35 (2.68-6.62)	0.12 (0.08-0.18)	-1.65 (-1.84--1.45)
HL	Republic of Lithuania	32.28 (28.05-36.64)	0.77 (0.67-0.88)	15.84 (12.71-19.43)	0.38 (0.31-0.46)	-2.48 (-2.77--2.18)
HL	Republic of Madagascar	75.14 (48.6-101.12)	0.86 (0.55-1.17)	149.64 (85.43-224.23)	0.7 (0.4-1.07)	-0.61 (-0.65--0.57)
HL	Republic of Malawi	41.49 (23.31-58.28)	0.56 (0.33-0.78)	62.6 (35.27-91.66)	0.45 (0.26-0.63)	-0.83 (-0.99--0.67)
HL	Republic of Maldives	0.55 (0.37-0.83)	0.41 (0.28-0.59)	0.56 (0.36-0.93)	0.13 (0.08-0.19)	-3.8 (-4--3.6)
HL	Republic of Mali	50.52 (30.2-71.47)	0.82 (0.55-1.3)	88.89 (57.44-145.56)	0.57 (0.38-1.01)	-1.06 (-1.18--0.94)
HL	Republic of Malta	3.35 (2.91-3.9)	0.81 (0.7-0.95)	2.57 (2.13-3.16)	0.33 (0.27-0.4)	-2.49 (-2.69--2.29)
HL	Republic of Mauritius	3.87 (3.59-4.14)	0.44 (0.41-0.47)	5.87 (5.26-6.46)	0.36 (0.32-0.39)	-2.42 (-3.42--1.42)
HL	Republic of Moldova	61.14 (54.79-68.44)	1.36 (1.23-1.53)	26.84 (22.84-31.26)	0.53 (0.45-0.62)	-3.04 (-3.22--2.85)
HL	Republic of Mozambique	108.32 (60.17-154.21)	1.09 (0.6-1.56)	220.49 (109.81-346.1)	1.04 (0.54-1.57)	0.31 (0.15-0.47)
HL	Republic of Namibia	5.63 (3.34-8.05)	0.6 (0.39-0.89)	9.66 (5.81-14.22)	0.51 (0.32-0.76)	-0.55 (-0.77--0.34)
HL	Republic of Nauru	0.01 (0-0.01)	0.11 (0.06-0.15)	0.01 (0-0.01)	0.08 (0.04-0.13)	-0.73 (-0.98--0.47)
HL	Republic of Nicaragua	8.48 (5.53-13.15)	0.36 (0.23-0.56)	12.38 (8.2-16.8)	0.22 (0.15-0.3)	-1.4 (-1.52--1.28)
HL	Republic of Niue	0 (0-0)	0.07 (0.04-0.1)	0 (0-0)	0.06 (0.03-0.08)	-1.27 (-1.5--1.04)
HL	Republic of Palau	0 (0-0.01)	0.03 (0.01-0.05)	0 (0-0.01)	0.02 (0.01-0.03)	-0.83 (-1.02--0.64)
HL	Republic of Panama	6.42 (5.84-7.08)	0.36 (0.33-0.4)	10.8 (8.59-13.07)	0.25 (0.2-0.3)	-1.09 (-1.21--0.98)
HL	Republic of Paraguay	10.01 (6.3-15.88)	0.34 (0.21-0.54)	17.4 (11.1-25.2)	0.27 (0.17-0.39)	-0.54 (-0.63--0.46)
HL	Republic of Peru	55.22 (38.82-82.16)	0.36 (0.25-0.52)	75.14 (42.78-107.1)	0.22 (0.12-0.31)	-1.65 (-1.8--1.49)
HL	Republic of Poland	551.64 (533.77-571.23)	1.31 (1.27-1.35)	193.8 (177.02-209.59)	0.33 (0.3-0.35)	-5.24 (-5.6--4.89)
HL	Republic of Rwanda	71.08 (44.36-96.81)	1.35 (0.85-1.77)	74.41 (41.51-119.47)	0.73 (0.43-1.13)	-2.62 (-2.85--2.39)
HL	Republic of San Marino	0.33 (0.2-0.48)	1.05 (0.64-1.51)	0.26 (0.16-0.42)	0.46 (0.27-0.71)	-1.76 (-2.08--1.45)
HL	Republic of Senegal	10 (5.07-14.31)	0.2 (0.1-0.27)	14.16 (9.18-22.27)	0.13 (0.09-0.19)	-1.37 (-1.47--1.27)

Cause	Location	1990		2021		EAPC No. (95% CI)
		Deaths cases (95% UI)	ASMR per 100,000 No. (95% UI)	Deaths cases (95% UI)	ASMR per 100,000 No. (95% UI)	
HL	Republic of Serbia	95.67 (57.28-139)	0.92 (0.56-1.34)	59.2 (39.53-87.33)	0.42 (0.28-0.62)	-2.83 (-3.02--2.63)
HL	Republic of Seychelles	0.35 (0.24-0.49)	0.55 (0.38-0.76)	0.34 (0.24-0.49)	0.3 (0.22-0.44)	-1.79 (-1.93--1.65)
HL	Republic of Sierra Leone	4.93 (2.32-6.97)	0.17 (0.08-0.23)	7.08 (4.46-11.14)	0.12 (0.08-0.18)	-1.14 (-1.24--1.04)
HL	Republic of Singapore	5.58 (4.87-6.44)	0.21 (0.19-0.25)	7.1 (5.84-8.67)	0.09 (0.08-0.11)	-1.57 (-1.97--1.16)
HL	Republic of Slovenia	14.29 (12.37-16.31)	0.61 (0.53-0.69)	6.62 (5.06-8.59)	0.18 (0.13-0.23)	-4.05 (-4.16--3.94)
HL	Republic of South Africa	86.84 (61.21-120.81)	0.3 (0.2-0.42)	144.54 (86.25-180.02)	0.27 (0.16-0.33)	-0.19 (-0.43-0.05)
HL	Republic of South Sudan	36.54 (22.29-53.16)	0.89 (0.54-1.3)	60.99 (35.42-90.67)	0.9 (0.53-1.32)	-0.02 (-0.24-0.21)
HL	Republic of Sudan	85.36 (54.86-157.93)	0.62 (0.39-1.25)	137.45 (79.31-228.72)	0.45 (0.27-0.75)	-0.89 (-0.98--0.8)
HL	Republic of Suriname	1.61 (1.07-2.48)	0.51 (0.35-0.8)	2.6 (1.52-3.61)	0.42 (0.25-0.59)	-0.52 (-0.68--0.36)
HL	Republic of Tajikistan	5.61 (3.55-8.15)	0.15 (0.09-0.22)	8.29 (5.16-12.99)	0.1 (0.06-0.15)	-1.61 (-1.82--1.4)
HL	Republic of the Congo	6.72 (4.7-10.27)	0.46 (0.32-0.7)	12.42 (8.11-18.64)	0.33 (0.22-0.5)	-1.25 (-1.37--1.13)
HL	Republic of the Gambia	2.81 (1.65-4.2)	0.43 (0.27-0.69)	6.29 (4.1-10.99)	0.36 (0.24-0.66)	-0.71 (-0.92--0.5)
HL	Republic of the Marshall Islands	0.03 (0.02-0.04)	0.1 (0.06-0.14)	0.04 (0.02-0.06)	0.08 (0.05-0.12)	-0.73 (-0.83--0.64)
HL	Republic of the Niger	10.53 (5.39-15.22)	0.21 (0.11-0.3)	18.04 (10.38-30.71)	0.14 (0.08-0.22)	-1.75 (-1.92--1.58)
HL	Republic of the Philippines	56.36 (37.84-78.03)	0.13 (0.08-0.19)	108.47 (68.14-140.15)	0.11 (0.07-0.14)	-0.16 (-0.23--0.08)
HL	Republic of the Union of Myanmar	140.42 (88.33-257.02)	0.44 (0.28-0.79)	102.21 (56.94-226.68)	0.2 (0.11-0.43)	-2.93 (-3.06--2.8)
HL	Republic of Trinidad and Tobago	4.69 (4.24-5.26)	0.47 (0.43-0.53)	4.46 (3.39-5.73)	0.26 (0.2-0.34)	-2.3 (-2.54--2.06)
HL	Republic of Tunisia	67.89 (45.17-103.98)	1.07 (0.7-1.71)	80.13 (50.94-128.49)	0.63 (0.4-1.01)	-1.77 (-1.81--1.73)
HL	Republic of Turkey	312.73 (218.96-546.71)	0.71 (0.5-1.23)	272.57 (187.51-442.98)	0.3 (0.21-0.49)	-2.85 (-3.01--2.7)
HL	Republic of Uganda	137.21 (83.34-201.85)	1.22 (0.75-1.83)	335.46 (196.43-493.98)	1.14 (0.69-1.63)	-0.59 (-0.83--0.35)
HL	Republic of Uzbekistan	79.2 (58.34-108.47)	0.5 (0.37-0.7)	107.22 (79.71-142.34)	0.34 (0.25-0.44)	-1.39 (-1.62--1.17)
HL	Republic of Vanuatu	0.09 (0.04-0.15)	0.09 (0.04-0.16)	0.18 (0.1-0.27)	0.07 (0.04-0.11)	-0.76 (-0.84--0.67)
HL	Republic of Yemen	41.64 (26.23-72.22)	0.57 (0.35-1.09)	102.6 (59.66-159.07)	0.5 (0.31-0.8)	-0.46 (-0.57--0.34)
HL	Republic of Zambia	55.78 (35.18-75.45)	1.01 (0.63-1.4)	104.39 (61.73-155.65)	0.76 (0.46-1.11)	-1.12 (-1.32--0.92)
HL	Republic of Zimbabwe	30.76 (17.36-47.54)	0.51 (0.31-0.8)	81.04 (48.4-116.35)	0.72 (0.45-1.04)	1.88 (1.38-2.39)
HL	Romania	239.82 (207.44-276.09)	0.94 (0.81-1.08)	104.93 (81.34-130.12)	0.35 (0.27-0.44)	-3.81 (-4.07--3.55)
HL	Russian Federation	1474.51 (1423.57-1531.53)	0.89 (0.86-0.92)	805 (738.91-872.14)	0.41 (0.38-0.44)	-2.98 (-3.27--2.68)
HL	Saint Kitts and Nevis	0.01 (0.01-0.01)	0.02 (0.02-0.02)	0.04 (0.04-0.05)	0.07 (0.06-0.08)	3.32 (1.49-5.19)
HL	Saint Lucia	0.88 (0.79-0.99)	0.86 (0.77-0.96)	1.01 (0.82-1.22)	0.46 (0.38-0.56)	-2.17 (-2.37--1.97)
HL	Saint Vincent and the Grenadines	0.64 (0.57-0.74)	0.75 (0.66-0.85)	0.58 (0.5-0.67)	0.44 (0.38-0.51)	-1.79 (-1.96--1.62)
HL	Slovak Republic	49.91 (31.13-67.98)	0.87 (0.54-1.18)	31.92 (20.88-48.62)	0.4 (0.26-0.6)	-2.57 (-2.66--2.47)
HL	Socialist Republic of Viet Nam	205.16 (133.99-339.41)	0.41 (0.27-0.69)	269.91 (177.74-415.75)	0.27 (0.18-0.41)	-1.16 (-1.22--1.1)
HL	Solomon Islands	0.23 (0.08-0.37)	0.11 (0.04-0.18)	0.45 (0.25-0.68)	0.09 (0.05-0.13)	-0.63 (-0.72--0.54)
HL	State of Eritrea	26.76 (16.24-36.62)	1.17 (0.71-1.57)	49.57 (28.42-72.43)	1.01 (0.59-1.45)	-0.34 (-0.4--0.27)
HL	State of Israel	34.46 (30.37-38.47)	0.71 (0.63-0.8)	29.66 (24.7-34.82)	0.26 (0.22-0.3)	-3.09 (-3.27--2.91)
HL	State of Kuwait	8.32 (7.26-9.41)	0.74 (0.65-0.84)	5.25 (4.14-6.49)	0.14 (0.11-0.17)	-2.03 (-3.91--0.12)
HL	State of Libya	38.68 (24.22-59.96)	1.38 (0.89-2.3)	68.39 (43.04-99.98)	1.08 (0.69-1.58)	-0.6 (-0.71--0.5)
HL	State of Qatar	0.91 (0.53-1.66)	0.42 (0.26-0.71)	3.24 (1.7-5.06)	0.18 (0.1-0.28)	-2.23 (-2.56--1.9)
HL	Sultanate of Oman	8.68 (4.36-12.88)	0.76 (0.39-1.11)	11.39 (5.5-16.51)	0.35 (0.18-0.49)	-1.83 (-2.03--1.62)
HL	Swiss Confederation	38.5 (34.35-43.82)	0.42 (0.37-0.48)	19.24 (15.62-23.47)	0.12 (0.1-0.15)	-3.75 (-4.09--3.42)
HL	Syrian Arab Republic	2.33 (1.51-3.26)	0.03 (0.02-0.04)	2.78 (1.37-3.99)	0.02 (0.01-0.03)	-0.93 (-1.26--0.6)
HL	Taiwan (Province of China)	19.24 (17.48-21.07)	0.11 (0.1-0.12)	16.41 (14.12-18.97)	0.04 (0.04-0.05)	-3.46 (-3.73--3.2)
HL	Togolese Republic	4.16 (2.18-5.64)	0.18 (0.1-0.25)	7.06 (4.61-10.25)	0.13 (0.08-0.18)	-1.38 (-1.53--1.23)
HL	Tokelau	0 (0-0)	0.09 (0.05-0.13)	0 (0-0)	0.07 (0.04-0.1)	-1.62 (-1.88--1.35)
HL	Turkmenistan	14.21 (12.42-16.56)	0.5 (0.44-0.57)	41 (31.52-53.1)	0.83 (0.65-1.08)	2.23 (1.72-2.73)
HL	Tuvalu	0.01 (0.01-0.01)	0.12 (0.06-0.17)	0.01 (0-0.01)	0.07 (0.04-0.1)	-1.68 (-1.76--1.59)
HL	Ukraine	394.56 (302.27-544.66)	0.66 (0.51-0.91)	298.39 (214.98-410.62)	0.54 (0.39-0.74)	-1.2 (-1.58--0.82)
HL	Union of the Comoros	3.25 (1.41-4.81)	0.97 (0.49-1.42)	4.99 (2.9-7.54)	0.78 (0.46-1.18)	-1.03 (-1.33--0.72)
HL	United Arab Emirates	7.3 (4.45-10.52)	0.73 (0.42-1.01)	21.37 (13.1-34.98)	0.38 (0.24-0.54)	-0.85 (-1.19--0.51)
HL	United Kingdom of Great Britain and Northern Ireland	485.6 (471.32-496.48)	0.64 (0.62-0.65)	343.69 (322.25-360.06)	0.32 (0.3-0.33)	-1.76 (-2.04--1.49)
HL	United Mexican States	541.97 (524.79-561.66)	0.99 (0.96-1.03)	678.87 (595.15-761.01)	0.53 (0.47-0.59)	-1.89 (-2.22--1.56)
HL	United Republic of Tanzania	174.08 (108.37-246.85)	0.93 (0.57-1.32)	297.07 (166.97-452.24)	0.68 (0.39-1.02)	-0.93 (-1--0.87)
HL	United States of America	1889.29 (1827.34-1938.8)	0.63 (0.61-0.65)	1242.65 (1146.35-1308.08)	0.24 (0.22-0.25)	-3.08 (-3.18--2.98)
HL	United States Virgin Islands	0.14 (0.09-0.25)	0.14 (0.1-0.27)	0.13 (0.07-0.18)	0.12 (0.07-0.18)	0.2 (-0.12-0.52)

Table S3.The number of HL and age standardized DALYs rate of leukemia caused by different reasons from 1990 to 2021.

Cause	Location	1990		2021		EAPC No. (95% CI)
		DALYs cases (95% UI)	ASDR per 100,000 No. (95% UI)	DALYs cases (95% UI)	ASDR per 100,000 No. (95% UI)	
HL	American Samoa	0.88 (0.5-1.47)	2.18 (1.27-3.55)	1.18 (0.6-1.74)	2.5 (1.27-3.73)	0.79 (0.49-1.1)
HL	Antigua and Barbuda	3.8 (3.37-4.44)	6.56 (5.82-7.52)	3.15 (2.84-3.54)	3.09 (2.79-3.47)	-2.12 (-2.34--1.91)
HL	Arab Republic of Egypt	2866.98 (1340.78-7314.28)	6.25 (2.9-16.22)	3498.92 (1747.88-8436.73)	3.88 (1.93-9.25)	-1.03 (-1.42--0.65)
HL	Argentine Republic	10280.74 (8496.76-12580.96)	31.68 (26.2-38.75)	6701.12 (5624.45-7948.01)	13.29 (11.13-15.76)	-2.42 (-2.79--2.04)
HL	Australia	2974.02 (2613.43-3360.68)	16.09 (14.1-18.2)	2668.13 (2180.75-3291.58)	8.15 (6.63-10.16)	-1.73 (-1.95--1.5)
HL	Barbados	53.53 (48.07-58.57)	19.66 (17.67-21.51)	40.57 (31.21-51.56)	11.08 (8.47-14.17)	-2.03 (-2.24--1.83)
HL	Belize	33.96 (28.73-48.9)	22.03 (18.52-31.72)	65.95 (55.98-77.08)	15.64 (13.32-18.27)	-1.16 (-1.57--0.74)
HL	Bermuda	21.96 (18.68-25.83)	33.38 (28.36-39.24)	9.42 (7.44-11.99)	11.41 (8.89-14.54)	-3.53 (-3.74--3.32)
HL	Bolivarian Republic of Venezuela	3414.3 (3015.54-3887.81)	22.31 (19.76-25.27)	5641.46 (4158.5-7433.84)	20.11 (14.87-26.39)	-0.48 (-0.69--0.27)
HL	Bosnia and Herzegovina	1355.34 (835.22-1825.56)	28.66 (17.73-38.31)	512.08 (339.12-827.12)	11.82 (7.72-19.43)	-3.26 (-3.52--3)
HL	Brunei Darussalam	33.81 (22.6-61.81)	15.75 (10.79-29.14)	56.03 (36.77-78.11)	11.92 (7.8-16.48)	-0.05 (-0.34-0.23)
HL	Burkina Faso	641.8 (318.59-908.32)	8.45 (4.3-11.76)	885.66 (578.5-1400.05)	4.94 (3.2-7.7)	-1.97 (-2.2--1.73)
HL	Canada	6712.78 (5965.73-7670.66)	22.08 (19.59-25.31)	3887.54 (3160.33-4732.37)	8.26 (6.68-10.08)	-3.06 (-3.18--2.94)
HL	Central African Republic	457.52 (320.36-707.33)	22.03 (15.44-33.33)	850.48 (563.8-1267.15)	19.61 (12.98-29.17)	-0.38 (-0.43--0.33)
HL	Commonwealth of Dominica	11.96 (7.18-18.13)	17.88 (10.87-27.16)	11.54 (7.2-17.79)	15.38 (9.62-23.68)	-0.42 (-0.6--0.24)
HL	Commonwealth of the Bahamas	46.97 (41.98-52.74)	19.31 (17.21-21.63)	53.49 (42.06-68.17)	12.59 (9.93-15.98)	-1.62 (-1.79--1.45)
HL	Cook Islands	0.45 (0.29-0.86)	2.66 (1.75-4.93)	0.31 (0.19-0.49)	1.65 (0.99-2.69)	-1 (-1.43--0.56)
HL	Czech Republic	4960.9 (4140.37-5950.37)	42.37 (35.12-51.02)	1733.4 (1340.07-2193.9)	12.14 (9.24-15.76)	-3.91 (-4.03--3.78)
HL	Democratic People's Republic of Korea	1725.91 (972.91-2603.57)	8.72 (4.95-13)	1762.7 (1183.33-3066.76)	5.73 (3.9-9.91)	-1.46 (-1.52--1.41)
HL	Democratic Republic of Sao Tome and Principe	0.26 (0.11-0.38)	0.27 (0.11-0.38)	0.4 (0.2-0.6)	0.21 (0.1-0.31)	-0.99 (-1.27--0.71)
HL	Democratic Republic of the Congo	4288.85 (2934.97-6789.91)	15.15 (10.09-24.26)	9430.55 (5854.89-15326.22)	13.63 (8.43-21.55)	-0.19 (-0.27--0.12)
HL	Democratic Republic of Timor-Leste	79.77 (45.69-156.2)	12.56 (7.06-24.93)	97.13 (48.8-228.89)	7.79 (3.85-18.32)	-1.57 (-2.03--1.11)
HL	Democratic Socialist Republic of Sri Lanka	3477.91 (2009.03-4523.2)	21.25 (12.24-27.63)	1811.08 (1104.86-3136.96)	7.5 (4.6-12.93)	-4 (-4.31--3.68)
HL	Dominican Republic	190.52 (103.3-438.03)	2.93 (1.57-6.97)	235.37 (118.86-526.58)	2.09 (1.06-4.7)	-0.9 (-1.24--0.56)
HL	Eastern Republic of Uruguay	924.24 (778.4-1101.92)	27.52 (23.19-32.9)	675.85 (561.63-808.79)	16.79 (13.89-20.1)	-1.63 (-2.03--1.24)
HL	Federal Democratic Republic of Ethiopia	40916.29 (26878.19-55190.17)	94.9 (60.03-128.42)	44591.46 (26485.55-72419.5)	45.1 (26.52-71.8)	-2.78 (-2.91--2.65)
HL	Federal Democratic Republic of Nepal	6463.46 (4418.46-10562.8)	36.32 (24.19-60.04)	5700.72 (3059.42-12152.47)	18.46 (9.92-38.91)	-2.11 (-2.31--1.91)
HL	Federal Republic of Germany	27972.83 (25314.82-30946.92)	28.52 (25.85-31.53)	10449.63 (8663.04-12550.37)	8.25 (6.92-9.89)	-3.72 (-4.12--3.32)
HL	Federal Republic of Nigeria	76502.62 (25303.41-119666.68)	95.85 (33.52-147.09)	129803.37 (38544.82-215853.92)	63.66 (20.38-103.43)	-1.49 (-1.59--1.39)
HL	Federal Republic of Somalia	3599.96 (2161.19-5070.6)	54.34 (32.33-77.24)	9238.91 (5364.87-14515.38)	52.24 (30.49-80)	-0.07 (-0.16-0.03)
HL	Federated States of Micronesia	4.02 (2.17-5.83)	4.75 (2.63-6.76)	3.21 (1.75-4.77)	3.17 (1.74-4.69)	-1.32 (-1.39--1.25)
HL	Federative Republic of Brazil	29510.03 (28213.85-30993.08)	22.34 (21.4-23.42)	25565.87 (23739.85-27365.87)	10.69 (9.95-11.43)	-2.11 (-2.29--1.94)
HL	French Republic	15108.64 (13279.31-16864.01)	23.13 (20.25-25.84)	7909.29 (6481.29-9704.33)	9.14 (7.52-11.17)	-2.67 (-2.85--2.49)
HL	Gabonese Republic	113.58 (77.09-171.96)	14.51 (9.74-22)	157.43 (100.14-245.89)	10 (6.44-15.23)	-1.25 (-1.38--1.12)
HL	Georgia	2819.2 (1732.26-3616.81)	48.52 (29.87-62.29)	1407.74 (1148.56-1715.84)	33.73 (26.62-41.65)	-1.27 (-1.85--0.7)
HL	Global	1324591.06 (959601.49-1519698.01)	26.05 (18.98-29.88)	1196188.22 (847435.78-1528816.43)	14.82 (10.43-18.96)	-1.88 (-1.94--1.83)
HL	Grand Duchy of Luxembourg	94.89 (86.92-103.19)	20.89 (19.18-22.68)	49.76 (43.06-57.24)	5.98 (5.19-6.88)	-3.97 (-4.15--3.8)
HL	Greenland	14.96 (8.8-19.81)	26 (15.34-34.22)	6.11 (4.21-10.55)	9.41 (6.43-16.49)	-3.4 (-3.54--3.26)
HL	Grenada	15.89 (12.97-19.69)	20.88 (17.12-25.84)	10.33 (8.41-12.56)	9.12 (7.42-11.1)	-2.49 (-2.64--2.35)
HL	Guam	6.26 (3.91-9.23)	4.66 (2.95-6.92)	7.59 (3.67-10)	4.71 (2.24-6.26)	0.44 (-0.19-1.06)
HL	Hashemite Kingdom of Jordan	362.96 (249.54-588.29)	12.45 (8.77-20.26)	756.41 (463.93-1094.61)	6.58 (4.05-9.45)	-2.06 (-2.25--1.87)
HL	Hellenic Republic	7225.21 (6758.95-7704.35)	59.34 (55.5-63.39)	3889.92 (3499.8-4279.49)	28.13 (25.5-31.09)	-2.38 (-2.72--2.04)
HL	High SDI	209507.81 (200842.06-217614.2)	21.21 (20.32-22.05)	108065.79 (99602.68-116259.09)	7.47 (6.79-8.08)	-3.27 (-3.36--3.19)
HL	High-middle SDI	282741.94 (224555.03-321732.35)	26.01 (20.61-29.61)	150856.48 (127241.46-169550.52)	9.62 (8.12-10.92)	-3.42 (-3.52--3.33)
HL	Hungary	4753.56 (4189.87-5399.65)	40.12 (35.49-45.37)	1169.94 (929.56-1490.17)	9.05 (7.19-11.38)	-5.11 (-5.27--4.95)
HL	Independent State of Papua New Guinea	144.92 (52.74-244.14)	4.06 (1.52-6.97)	283.74 (143.63-441.35)	2.96 (1.49-4.6)	-1.04 (-1.1--0.98)
HL	Independent State of Samoa	48.96 (28.97-95.47)	34.81 (21.01-68.62)	56.9 (33.24-111.49)	29.76 (17.28-59.14)	-0.36 (-0.5--0.22)
HL	Ireland	1083.78 (970.3-1212.57)	29.02 (25.94-32.35)	627.08 (515.23-748.27)	10.51 (8.59-12.59)	-2.53 (-2.79--2.27)
HL	Islamic Republic of Afghanistan	4116.05 (2447.73-7498.05)	49.45 (29.22-90.74)	10192.87 (6574.67-16208.9)	41.64 (26.73-65.55)	-0.56 (-0.72--0.4)
HL	Islamic Republic of Iran	5654.54 (4213.82-9245.26)	11.72 (8.75-19.63)	9155.58 (4669.78-11296.04)	10.55 (5.42-13.03)	0.78 (0.29-1.26)
HL	Islamic Republic of Mauritania	129.49 (71.44-184.98)	7.41 (4.11-10.23)	126.71 (79.51-215.64)	3.49 (2.25-5.7)	-2.75 (-2.86--2.64)
HL	Islamic Republic of Pakistan	62791.78 (38965.25-90838.78)	62.53 (40.54-95.17)	125293.35 (84296.61-180499.97)	54.93 (37.39-81.16)	-0.59 (-0.8--0.37)
HL	Jamaica	263.04 (223.73-308.14)	11.94 (10.26-13.96)	242.65 (175.31-330.91)	7.87 (5.69-10.72)	-1.56 (-1.98--1.13)
HL	Japan	4557.37 (4423.16-4700.32)	3.04 (2.95-3.14)	5192.48 (4765.62-5515.17)	2.44 (2.31-2.55)	-0.19 (-0.41--0.04)
HL	Kingdom of Bahrain	143.69 (89.13-208.85)	36.93 (22.37-50)	252.4 (155.94-385.44)	17.38 (10.72-25.18)	-2.52 (-2.77--2.28)
HL	Kingdom of Belgium	3116.79 (2697.5-3586.79)	26.31 (22.75-30.37)	1543.96 (1255.77-1879.91)	10.4 (8.43-12.64)	-2.94 (-3.13--2.75)
HL	Kingdom of Bhutan	193.59 (105.67-321.01)	34.48 (19.61-59.25)	135.9 (66.71-305.82)	17.49 (8.7-39.02)	-2.39 (-2.48--2.29)
HL	Kingdom of Cambodia	1534.74 (1009.67-2817.61)	18.51 (12.29-34.25)	1553.13 (788.86-3426.04)	9.47 (4.91-20.69)	-2.32 (-2.43--2.22)
HL	Kingdom of Denmark	1151.05 (1015.09-1314.5)	18.55 (16.41-21.1)	497.51 (406.45-600.58)	6.01 (4.99-7.17)	-3.38 (-3.72--3.05)
HL	Kingdom of Eswatini	120.01 (77.44-200.98)	20.1 (13.1-33.39)	216.53 (131.22-342.65)	21.05 (12.88-32.88)	0.49 (0.01-0.97)
HL	Kingdom of Lesotho	195.66 (114.89-342.76)	15.96 (9.33-28.14)	397.97 (257.96-652.51)	23.54 (15.02-38.43)	1.98 (1.57-2.39)
HL	Kingdom of Morocco	7474.75 (4603.67-10038.21)	32.28 (20.82-43.39)	7364.51 (4554.06-11345.88)	19.56 (12.11-30.03)	-1.51 (-1.65--1.37)
HL	Kingdom of Norway	768.09 (718.97-814.19)	14.68 (13.8-15.58)	646.19 (576.72-721.24)	8.59 (7.67-9.63)	-1.47 (-1.78--1.15)

Cause	Location	1990		2021		EAPC No. (95% CI)
		DALYs cases (95% UI)	ASDR per 100,000 No. (95% UI)	DALYs cases (95% UI)	ASDR per 100,000 No. (95% UI)	
HL	Kingdom of Saudi Arabia	2200.71 (684.67-3684.29)	16.95 (5.55-28.1)	4423.69 (1053.93-7840.12)	11.09 (2.83-19.16)	-1.02 (-1.23--0.8)
HL	Kingdom of Spain	13231.59 (11546.76-15245.86)	30.43 (26.49-35.16)	6322.74 (5230.47-7688.64)	10.11 (8.29-12.6)	-3.36 (-3.55--3.17)
HL	Kingdom of Sweden	1148.85 (992.18-1312.8)	10.46 (8.98-12.14)	772.25 (642.13-925)	5.3 (4.36-6.48)	-2.11 (-2.45--1.77)
HL	Kingdom of Thailand	4075.43 (2792.99-7000.27)	7.28 (4.97-13.12)	3192.02 (1949.06-6453.17)	4.17 (2.62-8.2)	-2.49 (-2.8--2.18)
HL	Kingdom of the Netherlands	3806.68 (3426.97-4271.59)	22.03 (19.74-24.76)	2390.04 (2021.8-2812.93)	10.24 (8.58-12.25)	-2.07 (-2.21--1.94)
HL	Kingdom of Tonga	1.79 (0.92-2.77)	2.28 (1.19-3.51)	1.66 (0.9-2.58)	1.78 (0.97-2.72)	-0.65 (-0.71--0.59)
HL	Kyrgyz Republic	1370.64 (1051.71-1753.8)	33.63 (26.09-42.6)	656.39 (482.9-862.17)	9.95 (7.4-12.98)	-2.13 (-3.02--1.24)
HL	Lao People's Democratic Republic	656.2 (425.56-1193.67)	19.24 (12.47-34.89)	617.56 (322.42-1421.65)	8.79 (4.65-20.07)	-2.7 (-2.77--2.63)
HL	Lebanese Republic	1348.15 (768.72-1818.55)	49.62 (28.49-67.11)	1326.85 (800.08-1764.08)	22.2 (13.34-29.43)	-2.52 (-2.61--2.43)
HL	Low SDI	204921.45 (116641.08-254461.28)	46.17 (27.01-58.33)	310628.28 (175856.68-424443)	30.28 (17.44-41.75)	-1.52 (-1.59--1.45)
HL	Low-middle SDI	312022.44 (204527.59-410803.13)	29.47 (19.31-39.82)	365163.4 (254654.84-521023.79)	19.23 (13.52-27.62)	-1.41 (-1.45--1.36)
HL	Malaysia	2297.47 (1170.99-3111.93)	15.02 (7.88-20.21)	2968.35 (1707.16-3738.03)	8.96 (5.17-11.27)	-1.89 (-2.13--1.66)
HL	Middle SDI	313804.3 (201728.53-372719.42)	20.02 (12.85-23.74)	260519.9 (186113.52-318967.32)	10.07 (7.16-12.25)	-2.25 (-2.34--2.15)
HL	Mongolia	328.6 (178.28-629.18)	17.9 (9.89-35.71)	478.15 (291.32-696.83)	14.8 (9.01-21.66)	-0.54 (-0.93--0.16)
HL	Montenegro	233.21 (142.86-321.56)	35.84 (21.98-49.57)	165.78 (111.34-236.6)	22.1 (14.79-31.92)	-1.69 (-2--1.38)
HL	New Zealand	474.7 (403.13-559.19)	12.57 (10.69-14.8)	409.15 (343.87-484.2)	6.21 (5.23-7.4)	-2.37 (-3.16--1.58)
HL	North Macedonia	981.48 (589.44-1429.47)	48.16 (28.97-70.07)	580.69 (386.14-944.66)	21.14 (13.95-34.65)	-2.95 (-3.14--2.75)
HL	Northern Mariana Islands	0.46 (0.25-0.71)	0.99 (0.53-1.53)	0.52 (0.24-0.72)	0.99 (0.45-1.4)	0.27 (0-0.53)
HL	Palestine	607.84 (351.19-839.42)	39.82 (22.19-55.78)	953.91 (536.02-1244.11)	22.52 (12.21-29.01)	-1.66 (-1.78--1.53)
HL	People's Democratic Republic of Algeria	10558.27 (6259.39-14158.09)	45.99 (28.51-61.91)	10369.34 (6715.08-14914.38)	24.26 (15.79-34.97)	-1.95 (-1.99--1.92)
HL	People's Republic of Bangladesh	42665.9 (29825.71-68075.05)	42.61 (28.91-70.59)	30094 (16585.57-62758.99)	18.01 (10-37.51)	-2.77 (-2.86--2.69)
HL	People's Republic of China	182007.04 (78811.42-252106.29)	16.78 (7.24-23.16)	74190.62 (46299.49-100603.17)	4.13 (2.63-5.62)	-4.98 (-5.21--4.74)
HL	Plurinational State of Bolivia	1984.73 (1127.91-2715.64)	36.75 (21.46-49.28)	2056.24 (1144.31-3291.29)	18.61 (10.48-29.35)	-2.32 (-2.41--2.23)
HL	Portuguese Republic	3034.64 (2715.8-3375.77)	27.05 (24.17-30.22)	1300.04 (1070.56-1595.63)	9.11 (7.47-11.31)	-3.6 (-3.74--3.45)
HL	Principality of Andorra	9.3 (5.41-13.82)	14.78 (8.67-21.88)	8.98 (5.18-13.62)	8.25 (4.78-12.51)	-1.6 (-1.73--1.47)
HL	Principality of Monaco	20.37 (10.61-28.55)	52 (27.85-74.28)	17.74 (10.73-29.18)	38.01 (23.07-63.24)	-1.07 (-1.1--1.04)
HL	Puerto Rico	1326.56 (1148.15-1540.2)	36.23 (31.38-42.04)	597.85 (468.63-740.95)	14.71 (11.64-18.2)	-3.03 (-3.2--2.87)
HL	Republic of Albania	901.88 (571.24-1342.8)	29.06 (18.38-43.03)	367.4 (243.21-585.19)	11.43 (7.4-17.66)	-3.06 (-3.26--2.85)
HL	Republic of Angola	1377.95 (953.57-2011.59)	17.72 (12.07-27.3)	2999.86 (1879.82-4699.2)	12.49 (7.82-19.28)	-1.07 (-1.16--0.97)
HL	Republic of Armenia	557.13 (435.46-656.59)	16.53 (13.13-19.36)	308.96 (251.31-368.38)	8.87 (7.25-10.61)	-2.06 (-2.39--1.73)
HL	Republic of Austria	2930.44 (2629.89-3244.58)	31.12 (27.95-34.58)	1220.43 (1031.13-1431.86)	10.14 (8.47-12.02)	-3.38 (-3.68--3.08)
HL	Republic of Azerbaijan	2469.4 (1514.61-3745.64)	34.3 (21.2-51.72)	2329.95 (1397.52-3635.14)	20.83 (12.44-32)	-1.9 (-2.14--1.66)
HL	Republic of Belarus	3629.13 (3042.11-4417.81)	32.26 (26.93-39.34)	2372.28 (1826.35-3150.81)	20.84 (15.85-27.53)	-1.61 (-1.94--1.28)
HL	Republic of Benin	308.76 (157.72-437.26)	7.88 (4.14-10.9)	469.54 (292.94-771.95)	4.35 (2.73-6.76)	-2.22 (-2.42--2.02)
HL	Republic of Botswana	204.99 (124.07-358.31)	19.87 (12.06-34.17)	300.23 (166.16-512.95)	12.86 (7.26-21.61)	-1.37 (-1.55--1.18)
HL	Republic of Bulgaria	4506.87 (3758.19-5367.77)	46.72 (39.07-55.38)	1732.09 (1340.91-2219.93)	19.88 (15.46-25.14)	-2.68 (-2.84--2.52)
HL	Republic of Burundi	2695.98 (1568.37-3751.59)	52.78 (30.91-73.3)	4669.61 (2385.9-7347.49)	39.55 (20.25-61.08)	-1.02 (-1.09--0.95)
HL	Republic of Cabo Verde	2.1 (0.65-3.51)	0.71 (0.22-1.14)	1.65 (0.87-2.43)	0.3 (0.16-0.43)	-2.65 (-3--2.3)
HL	Republic of Cameroon	697.86 (353.52-990.53)	8.12 (4.21-11.16)	1249.87 (764.5-2020.02)	4.72 (2.85-7.4)	-2.03 (-2.24--1.81)
HL	Republic of Chad	386.93 (201.1-562.41)	7.71 (4.12-11.05)	816.25 (529.9-1364.18)	6.1 (4.02-9.65)	-1 (-1.24--0.75)
HL	Republic of Chile	1454.33 (1231.61-1735.49)	11.63 (9.89-13.86)	2228.69 (1871.5-2670.54)	10.2 (8.56-12.28)	0.13 (-0.61-0.87)
HL	Republic of Colombia	7793.94 (7051.37-8639.9)	26.54 (23.99-29.18)	6742.92 (5492.69-8371.85)	12.71 (10.38-15.79)	-2.6 (-2.79--2.4)
HL	Republic of Costa Rica	1259.08 (1100.52-1430.02)	45.85 (40.29-51.78)	1057.06 (869.8-1279.8)	20.12 (16.48-24.4)	-2.76 (-3.1--2.42)
HL	Republic of C 么 te d'Ivoire	2354.96 (1330.27-3438.86)	22.6 (13.91-33.84)	4320.78 (2436.15-6507.93)	17.28 (10.55-26.68)	-0.74 (-0.88--0.6)
HL	Republic of Croatia	1633.93 (1438.71-1853.67)	29.47 (25.87-33.42)	480.7 (397.16-576.71)	8.24 (6.8-9.86)	-4.14 (-4.46--3.82)
HL	Republic of Cuba	5837.32 (4708.97-6855.77)	50.42 (40.76-59.25)	3700.97 (3055.35-4511.72)	26.01 (21.26-31.72)	-2.41 (-2.6--2.23)
HL	Republic of Cyprus	197.72 (131.76-307.28)	24.95 (16.57-38.23)	175.4 (116.43-272.68)	10.15 (6.73-15.86)	-3.04 (-3.2--2.88)
HL	Republic of Djibouti	132.62 (72.65-210.73)	35.28 (19.69-54.43)	364.21 (200.16-596.71)	30.52 (17.07-49.62)	-0.48 (-0.78--0.18)
HL	Republic of Ecuador	1126.46 (923.81-1361.56)	13.41 (11.23-15.89)	2763.5 (2131.38-3548.27)	15.61 (12.04-20.02)	0.94 (0.42-1.46)
HL	Republic of El Salvador	948.71 (623.76-1298.97)	21.01 (13.54-29.32)	825.13 (528.1-1117.5)	12.88 (8.21-17.48)	-1.54 (-1.75--1.32)
HL	Republic of Equatorial Guinea	60.64 (39.99-96.76)	19.19 (12.71-30.42)	107.45 (61.98-179.65)	8.82 (5.19-14.4)	-2.86 (-3.14--2.58)
HL	Republic of Estonia	535.71 (427.8-664.26)	31.56 (24.98-39.4)	182.51 (136.77-230.15)	11.59 (8.6-14.8)	-3.62 (-3.94--3.3)
HL	Republic of Fiji	65.21 (36.99-143.05)	9.23 (5.3-20.2)	102.93 (61.38-163.92)	11.19 (6.69-17.76)	1.48 (1.03-1.93)
HL	Republic of Finland	1292.83 (1161.31-1471.31)	22.06 (19.69-25.16)	585.88 (488.44-686.54)	7.88 (6.55-9.35)	-2.65 (-2.97--2.33)
HL	Republic of Ghana	163.24 (54.98-265.92)	1.35 (0.43-2.2)	198.92 (99.25-298.15)	0.66 (0.33-0.97)	-2.76 (-3.09--2.44)
HL	Republic of Guatemala	1127.53 (898.84-1287.77)	16.85 (14.39-18.69)	1073.92 (895.55-1252.11)	7.4 (6.19-8.67)	-2.56 (-2.73--2.38)
HL	Republic of Guinea	1461.37 (823.1-2091.37)	26.07 (16.76-38.29)	2579.04 (1533.91-4070.38)	21.17 (13.57-33.23)	-0.54 (-0.62--0.47)
HL	Republic of Guinea-Bissau	108.05 (57.04-159.31)	12.77 (6.85-18.73)	129.68 (84.42-201.65)	7.58 (5.13-11.68)	-1.88 (-2.01--1.75)
HL	Republic of Guyana	149.61 (120.56-187.77)	22.38 (18.58-27.28)	131.63 (100.66-170.04)	16.99 (12.97-21.9)	-0.63 (-0.87--0.39)
HL	Republic of Haiti	1902.67 (1264.76-2817.16)	35.92 (23.66-53.49)	3452.7 (1834.11-5348.44)	29.22 (15.56-45.05)	-0.52 (-0.61--0.44)
HL	Republic of Honduras	465.08 (293.71-619.66)	12.05 (7.72-16.02)	699.68 (424.64-1062.65)	8.24 (4.99-12.28)	-1.32 (-1.41--1.23)
HL	Republic of Iceland	45.7 (39.79-52.98)	16.98 (14.77-19.67)	31.4 (25.59-38.28)	7.56 (6.1-9.23)	-2.07 (-2.31--1.83)
HL	Republic of India	228484.51 (131940.41-286054.08)	28.66 (16.73-35.95)	215853.3 (149156.54-318060.53)	14.94 (10.32-21.99)	-2.16 (-2.24--2.08)
HL	Republic of Indonesia	20271.63 (14106.01-37059.29)	11.99 (8.25-22.35)	19802.01 (11456-42925.13)	6.9 (4.03-14.91)	-1.77 (-1.81--1.73)
HL	Republic of Iraq	4833.5 (2326.92-6898.27)	31.82 (15.57-44.78)	6124.83 (2981.58-9166.85)	16.43 (8.1-24.07)	-2.39 (-2.61--2.16)
HL	Republic of Italy	22697.54 (21496.02-24047.22)	33.68 (31.89-35.75)	11838.28 (10768.21-13037.71)	13.84 (12.65-15.2)	-2.29 (-2.6--1.97)
HL	Republic of Kazakhstan	3342.2 (2708.26-4806.79)	20.65 (16.76-29.63)	2203.91 (1828.37-2620.97)	11.36 (9.4-13.51)	-2.49 (-3.14--1.83)
HL	Republic of Kenya	3388.7 (2034.85-4978.78)	17.43 (10.51-26.02)	7442.21 (4381.77-11525.28)	16.44 (9.53-24.58)	0.28 (0.05-0.52)
HL	Republic of Kiribati	1.71 (0.68-2.7)	2.51 (1-3.87)	2.53 (0.95-4.24)	2.17 (0.82-3.51)	-0.33 (-0.4--0.26)
HL	Republic of Korea	1895.87 (1381.22-3327.97)	4.45 (3.21-7.77)	1247.81 (716.98-1745.36)	1.75 (1.04-2.45)	-2.65 (-2.84--2.46)
HL	Republic of Latvia	835.18 (686.74-1000.3)	28.94 (23.72-34.84)	355.84 (265.73-457.64)	15.46 (11.52-19.85)	-2.38 (-2.63--2.13)
HL	Republic of Liberia	166.4 (82.53-244.42)	7.89 (3.94-11.2)	227.3 (138.06-359.24)	4.8 (2.96-7.27)	-1.81 (-2.03--1.59)
HL	Republic of Lithuania	1255.87 (1087.47-1423.14)	31.79 (27.47-36.05)	538.93 (434-651.23)	16.07 (13.06-19.51)	-2.49 (-2.84--2.14)

Cause	Location	1990		2021		EAPC No. (95% CI)
		DALYs cases (95% UI)	ASDR per 100,000 No. (95% UI)	DALYs cases (95% UI)	ASDR per 100,000 No. (95% UI)	
HL	Republic of Madagascar	4291.9 (2776.5-5906.3)	39.17 (25.31-52.75)	8576.8 (4984.56-13011.86)	31.91 (18.19-47.75)	-0.6 (-0.64--0.55)
HL	Republic of Malawi	2521.17 (1411.13-3654.68)	26.33 (14.96-36.44)	3576.52 (1940.37-5399.34)	19.85 (11.24-28.99)	-0.97 (-1.12--0.82)
HL	Republic of Maldives	27.79 (18.16-43.24)	16.22 (10.92-24.71)	25.27 (15.74-45.1)	4.84 (3.13-7.78)	-3.95 (-4.12--3.77)
HL	Republic of Mali	2817.37 (1520.78-3950.78)	36.01 (22.05-52.45)	5002.82 (3068.4-7700.49)	23.98 (15.58-40.17)	-1.18 (-1.31--1.06)
HL	Republic of Malta	117.28 (101.29-136.43)	29.29 (25.21-34.33)	76.35 (63.71-93.65)	12.95 (10.79-15.95)	-2.09 (-2.32--1.86)
HL	Republic of Mauritius	168.59 (156.99-179.83)	16.39 (15.28-17.5)	214.42 (192.39-235.37)	14.05 (12.59-15.41)	-2.26 (-3.28--1.22)
HL	Republic of Moldova	2770.59 (2469.65-3114.51)	61.35 (54.71-68.93)	995.31 (842.07-1163.67)	22.43 (18.91-26.22)	-3.24 (-3.44--3.04)
HL	Republic of Mozambique	6281.88 (3502.7-8747.64)	49.5 (27.13-71.18)	12609.19 (6090.92-20096.05)	45.96 (22.98-71.75)	0.24 (0.06-0.41)
HL	Republic of Namibia	286.31 (160.87-405.06)	24.55 (14.65-35.65)	466.1 (269.01-706.43)	20.8 (12.27-30.89)	-0.58 (-0.79--0.37)
HL	Republic of Nauru	0.4 (0.21-0.57)	4.54 (2.45-6.47)	0.36 (0.17-0.56)	3.6 (1.75-5.46)	-0.72 (-0.99--0.45)
HL	Republic of Nicaragua	433.87 (287.57-658.39)	13.85 (9.05-21.69)	496.19 (329.77-713.43)	8.01 (5.33-11.23)	-1.62 (-1.73--1.5)
HL	Republic of Niue	0.06 (0.03-0.09)	2.89 (1.48-4.3)	0.05 (0.03-0.06)	2.79 (1.68-3.72)	-1.15 (-1.51--0.79)
HL	Republic of Palau	0.17 (0.09-0.32)	1.16 (0.58-2.15)	0.17 (0.09-0.26)	0.89 (0.44-1.46)	-0.56 (-0.79--0.34)
HL	Republic of Panama	270.51 (245.42-299.26)	12.84 (11.71-14.13)	377.62 (303.46-455.33)	8.62 (6.93-10.4)	-1.08 (-1.23--0.94)
HL	Republic of Paraguay	475.47 (300.44-759.8)	13.72 (8.63-22.12)	720.82 (465.58-1057.35)	10.25 (6.57-15.06)	-0.71 (-0.8--0.62)
HL	Republic of Peru	2514.84 (1772.09-3852.65)	13.46 (9.58-20.24)	2658.02 (1623.42-3755.48)	7.39 (4.48-10.43)	-1.91 (-2.06--1.76)
HL	Republic of Poland	21621.49 (20927-22372.32)	52.66 (50.94-54.48)	6251.91 (5750.43-6769.81)	12.66 (11.67-13.71)	-5.28 (-5.59--4.97)
HL	Republic of Rwanda	4182.81 (2643.12-5828.38)	62.86 (39.11-84.81)	4011.64 (2232.94-6675.67)	32.09 (17.84-51.97)	-2.81 (-3.03--2.59)
HL	Republic of San Marino	11.3 (6.87-15.96)	39.7 (23.99-55.46)	8.05 (4.6-12.64)	19.48 (11.06-30.27)	-1.53 (-1.81--1.26)
HL	Republic of Senegal	537.49 (264.97-778.85)	8.26 (4.21-11.82)	698.81 (446.27-1146.86)	5.04 (3.24-8.03)	-1.62 (-1.75--1.5)
HL	Republic of Serbia	3688.33 (2223.9-5319.42)	35.46 (21.57-51.19)	1841.63 (1215.61-2747.71)	15.39 (10.07-23.43)	-2.95 (-3.16--2.74)
HL	Republic of Seychelles	15.04 (10.6-20.81)	21.87 (15.17-29.9)	13.11 (9.42-18.92)	11.42 (8.24-16.62)	-1.84 (-2--1.67)
HL	Republic of Sierra Leone	248.64 (111.43-367.03)	6.93 (3.22-9.93)	359.12 (225.69-577.15)	4.7 (2.97-7.43)	-1.31 (-1.39--1.23)
HL	Republic of Singapore	226.02 (193.45-263.67)	7.44 (6.45-8.62)	212.4 (175.65-254.73)	3.04 (2.52-3.64)	-1.71 (-2.17--1.25)
HL	Republic of Slovenia	534.82 (461.07-607.54)	24.14 (20.84-27.39)	193.21 (144.63-253.01)	6.68 (5.01-8.82)	-4.06 (-4.21--3.92)
HL	Republic of South Africa	4373.67 (3144.85-6235.43)	13.06 (9.22-18.29)	6349.16 (3841.02-7914.65)	10.91 (6.57-13.55)	-0.49 (-0.78--0.19)
HL	Republic of South Sudan	2002.05 (1245.5-2872.53)	38.07 (23.02-55.12)	3448.68 (1972.02-5147.36)	39.59 (22.95-58.53)	0.08 (-0.21-0.37)
HL	Republic of Sudan	4374.41 (2761-7806.38)	25.9 (16.59-48)	7037.21 (4004.24-11635.53)	18.33 (10.61-30.39)	-0.93 (-1.04--0.82)
HL	Republic of Suriname	74.09 (46.75-110.11)	20.63 (13.48-31.18)	106.92 (63.19-148.22)	17.58 (10.4-24.37)	-0.51 (-0.66--0.37)
HL	Republic of Tajikistan	288.15 (185.26-416.7)	6.27 (4.04-9.12)	421.96 (262.08-669.73)	4.37 (2.71-6.86)	-1.61 (-1.8--1.42)
HL	Republic of the Congo	317.02 (218.5-495.82)	17.66 (12.31-27.13)	583.06 (380.58-887)	12.51 (8.22-18.83)	-1.23 (-1.37--1.08)
HL	Republic of the Gambia	159.95 (86.99-238.63)	18.5 (11.09-28.13)	346.62 (220.06-594.37)	15.71 (10.27-27.53)	-0.76 (-1.01--0.51)
HL	Republic of the Marshall Islands	1.51 (0.82-2.01)	4.38 (2.44-5.75)	2 (1.03-2.9)	3.65 (1.92-5.27)	-0.67 (-0.78--0.55)
HL	Republic of the Niger	591.65 (301.35-858.49)	9 (4.62-12.98)	941.73 (541.35-1642.16)	5.11 (2.92-8.61)	-2.19 (-2.39--1.98)
HL	Republic of the Philippines	2900.45 (1936.25-3931.61)	5.21 (3.5-7.22)	4851.13 (3078.19-6298.99)	4.41 (2.79-5.69)	-0.32 (-0.41--0.22)
HL	Republic of the Union of Myanmar	7095.84 (4399.55-13054.73)	18.63 (11.73-34.3)	4379.63 (2378.19-9853.46)	7.74 (4.23-17.38)	-3.23 (-3.38--3.07)
HL	Republic of Trinidad and Tobago	203.63 (183.32-230.45)	18.36 (16.57-20.69)	171.48 (129.12-221.84)	11.01 (8.34-14.17)	-2.02 (-2.27--1.76)
HL	Republic of Tunisia	3252.94 (2097.06-4559.44)	42.5 (27.95-63.46)	3097.87 (1958.03-4832.07)	24.68 (15.51-38.03)	-1.8 (-1.84--1.77)
HL	Republic of Turkey	14793.26 (10352.48-25465.41)	28.21 (19.85-49.24)	9822.85 (6771.42-16624.87)	10.81 (7.53-18.4)	-3.16 (-3.26--3.06)
HL	Republic of Uganda	7784.61 (4673.63-11214.68)	51.6 (31.06-76.12)	19569.93 (11169.47-29400.25)	50.03 (29.67-73.43)	-0.51 (-0.77--0.25)
HL	Republic of Uzbekistan	4091.67 (3014.81-5463.08)	21.97 (16.18-29.94)	4994.7 (3661.82-6695.91)	14.58 (10.72-19.52)	-1.45 (-1.67--1.22)
HL	Republic of Vanuatu	4.63 (2.03-7.63)	3.79 (1.66-6.42)	8.97 (4.95-13.09)	3.19 (1.78-4.68)	-0.74 (-0.83--0.65)
HL	Republic of Yemen	2043.53 (1294.65-3397.03)	21.56 (13.4-37.74)	4859.28 (2684.49-7517.98)	18.46 (10.71-28.69)	-0.52 (-0.68--0.37)
HL	Republic of Zambia	3284.27 (2081.94-4422.33)	45.8 (28.77-62.16)	5908.51 (3440.5-8933.5)	33.57 (19.87-49.68)	-1.17 (-1.38--0.97)
HL	Republic of Zimbabwe	1541.49 (820.57-2318.21)	19.75 (11.24-31.32)	4237.94 (2469.44-6117.9)	31.07 (18.56-44.95)	2.31 (1.75-2.88)
HL	Romania	10012.05 (8574.65-11698.15)	40.66 (34.6-47.65)	3343.39 (2562.31-4238.82)	13.55 (10.39-17.21)	-4.1 (-4.34--3.87)
HL	Russian Federation	67151.01 (64652.46-69819)	42.07 (40.46-43.77)	30486.02 (28060.97-33089.64)	17.67 (16.24-19.15)	-3.33 (-3.65--3.01)
HL	Saint Kitts and Nevis	0.33 (0.3-0.36)	0.85 (0.76-0.94)	1.56 (1.28-1.94)	2.33 (1.91-2.87)	2.75 (0.96-4.57)
HL	Saint Lucia	38.53 (34.48-44.1)	33.34 (29.79-37.52)	39.13 (31.79-47.53)	19.17 (15.68-23.27)	-1.93 (-2.09--1.77)
HL	Saint Vincent and the Grenadines	29.33 (25.94-33.67)	30.35 (26.91-34.81)	22.46 (19.22-26.2)	17.97 (15.41-20.95)	-1.8 (-1.98--1.63)
HL	Slovak Republic	1920.18 (1199.31-2601.91)	34.21 (21.39-46.29)	1070.65 (693.99-1626.67)	15.58 (9.96-23.63)	-2.52 (-2.63--2.41)
HL	Socialist Republic of Viet Nam	9491.9 (5977.07-14902.54)	15.98 (10.38-26.14)	10695.23 (6936.5-15986.92)	10.32 (6.7-15.33)	-1.27 (-1.35--1.2)
HL	Solomon Islands	11.78 (4.07-19.56)	4.49 (1.58-7.44)	22.55 (12.2-34.39)	3.73 (2.05-5.65)	-0.56 (-0.64--0.47)
HL	State of Eritrea	1566.85 (944.77-2181.19)	52.12 (31.83-70.55)	2726.36 (1558.34-4079.17)	44.44 (25.49-64.76)	-0.38 (-0.46--0.31)
HL	State of Israel	1247.63 (1096.78-1410.84)	25.86 (22.78-29.22)	965.59 (800.03-1133.5)	9.44 (7.83-11.11)	-3.09 (-3.29--2.88)
HL	State of Kuwait	446.34 (385.78-509.13)	29.56 (25.86-33.28)	237.4 (186.95-291.1)	5.08 (4.03-6.22)	-2.29 (-4.19--0.36)
HL	State of Libya	1961.08 (1209.62-2826.45)	55.84 (35.02-86.54)	3093.31 (1866.6-4512.7)	43.34 (26.32-62.59)	-0.6 (-0.72--0.47)
HL	State of Qatar	46 (26.83-83.99)	13.65 (8.36-24.01)	165.01 (87.54-255.15)	6.22 (3.36-9.37)	-1.95 (-2.29--1.61)
HL	Sultanate of Oman	437.57 (213.06-658.32)	28.63 (14.47-42.45)	552.96 (260.7-823.47)	12.95 (6.36-18.34)	-1.84 (-2.07--1.61)
HL	Swiss Confederation	1382.71 (1217.39-1582.02)	17.18 (15.1-19.61)	551.28 (447.83-671.43)	4.59 (3.71-5.71)	-4.15 (-4.5--3.8)
HL	Syrian Arab Republic	118.33 (77.58-162.64)	1.17 (0.75-1.62)	109.3 (55.03-156.87)	0.76 (0.39-1.1)	-1.17 (-1.54--0.79)
HL	Taiwan (Province of China)	827.12 (736.26-915.5)	4.12 (3.68-4.54)	552.05 (473.9-644.54)	1.7 (1.44-2.04)	-3.41 (-3.66--3.15)
HL	Togolese Republic	230.84 (118.2-320.8)	7.65 (4.04-10.32)	349.93 (224.87-525.38)	4.91 (3.2-7.14)	-1.65 (-1.81--1.49)
HL	Tokelau	0.06 (0.03-0.08)	3.77 (2.04-5.68)	0.05 (0.03-0.07)	3.43 (2.08-5.09)	-1.43 (-1.86--0.99)
HL	Turkmenistan	757.02 (649.25-907.67)	22.55 (19.61-26.44)	2009.1 (1540.54-2654.4)	38.52 (29.61-50.52)	2.27 (1.77-2.77)
HL	Tuvalu	0.44 (0.24-0.61)	5.03 (2.75-6.97)	0.35 (0.2-0.5)	2.85 (1.62-4.11)	-1.77 (-1.88--1.67)
HL	Ukraine	17855.03 (13705.66-24447.16)	32.26 (24.67-43.92)	12397.24 (8912.34-17180.8)	25.54 (18.61-35.51)	-1.35 (-1.73--0.96)
HL	Union of the Comoros	184.59 (77.39-279.26)	43.9 (19.01-65.24)	251.55 (145.03-379.91)	34.44 (19.96-52.06)	-1.2 (-1.62--0.78)
HL	United Arab Emirates	378.1 (229.8-543.26)	26.09 (15.44-37.44)	979.21 (594.26-1616.17)	12.24 (7.71-17.38)	-1.62 (-1.86--1.39)
HL	United Kingdom of Great Britain and Northern Ireland	17194.58 (16775.1-17627.74)	25.79 (25.15-26.46)	10780 (10278.06-11308.58)	12.73 (12.15-13.37)	-1.8 (-2.07--1.52)
HL	United Mexican States	24684.46 (23829.54-25633.94)	34.84 (33.77-36.09)	24046.05 (21375.75-26835.75)	18.07 (16.08-20.14)	-1.97 (-2.33--1.62)
HL	United Republic of Tanzania	10042.35 (6176.26-14262.6)	42.05 (26.14-59.78)	16550.89 (9242.32-25776.9)	30.5 (17.09-46.41)	-0.92 (-1--0.85)
HL	United States of America	75891.13 (73775.9-78219.27)	26.74 (26-27.57)	37269.34	8.57 (8.15-9.03)	-3.62 (-3.76--3.48)

Cause	Location	1990		2021		EAPC No. (95% CI)
		DALYs cases (95% UI)	ASDR per 100,000 No. (95% UI)	DALYs cases (95% UI)	ASDR per 100,000 No. (95% UI)	
				(35310.55-39283.38)		
HL	United States Virgin Islands	6.02 (4.01-10.61)	5.87 (3.93-10.43)	4.98 (2.73-7.26)	6.15 (3.24-9.42)	1.1 (0.81-1.4)

Table S4. The incident cases and age standardized incidence of HL caused by different reasons from 1990 to 2021.

Cause	Location	1990		2021		EAPC No. (95% CI)
		Incident cases (95% UI)	ASIR per 100,000 No. (95% UI)	Incident cases (95% UI)	ASIR per 100,000 No. (95% UI)	
HL	American Samoa	0.03 (0.02-0.05)	0.08 (0.05-0.13)	0.05 (0.03-0.08)	0.11 (0.06-0.17)	1.27 (1.01-1.52)
HL	Antigua and Barbuda	0.14 (0.12-0.16)	0.24 (0.22-0.28)	0.17 (0.15-0.2)	0.17 (0.15-0.2)	-0.82 (-1.04--0.61)
HL	Arab Republic of Egypt	73.91 (34.16-192.07)	0.19 (0.09-0.5)	160.76 (77.07-395.57)	0.18 (0.09-0.45)	0.27 (-0.06-0.61)
HL	Argentine Republic	439.29 (366.86-538.42)	1.36 (1.13-1.66)	533.67 (439-642.45)	1.08 (0.88-1.3)	-0.42 (-0.78--0.06)
HL	Australia	334.13 (289.22-385.94)	1.84 (1.59-2.14)	615.79 (498.65-758.33)	2.09 (1.67-2.59)	0.81 (0.41-1.21)
HL	Barbados	2.09 (1.87-2.34)	0.75 (0.68-0.84)	2.33 (1.78-2.99)	0.65 (0.5-0.84)	-0.69 (-0.94--0.44)
HL	Belize	0.99 (0.81-1.42)	0.69 (0.57-0.98)	2.62 (2.2-3.11)	0.63 (0.53-0.75)	-0.26 (-0.63-0.1)
HL	Bermuda	1.1 (0.94-1.3)	1.69 (1.44-2)	1.17 (0.91-1.53)	1.72 (1.31-2.24)	0.06 (-0.16-0.28)
HL	Bolivarian Republic of Venezuela	102.75 (90.58-117.19)	0.74 (0.66-0.84)	264.98 (193.23-349.17)	0.96 (0.7-1.25)	0.84 (0.69-0.99)
HL	Bosnia and Herzegovina	59.26 (37.49-80.36)	1.27 (0.8-1.71)	44.94 (29.73-72.26)	1.18 (0.77-1.92)	-0.16 (-0.3--0.02)
HL	Brunei Darussalam	0.88 (0.6-1.59)	0.48 (0.33-0.89)	2.14 (1.42-2.98)	0.48 (0.32-0.66)	0.86 (0.58-1.14)
HL	Burkina Faso	14.28 (7.23-19.83)	0.22 (0.11-0.31)	22.04 (14.24-34.6)	0.14 (0.09-0.22)	-1.61 (-1.8--1.41)
HL	Canada	857.4 (741.2-1001.73)	2.9 (2.49-3.42)	869.52 (695.7-1057.83)	2.15 (1.7-2.63)	-0.87 (-1.2--0.54)
HL	Central African Republic	9.97 (6.94-15)	0.57 (0.4-0.88)	18.91 (12.47-27.84)	0.52 (0.34-0.78)	-0.33 (-0.38--0.29)
HL	Commonwealth of Dominica	0.38 (0.23-0.57)	0.57 (0.36-0.86)	0.44 (0.28-0.66)	0.57 (0.36-0.88)	-0.01 (-0.11-0.09)
HL	Commonwealth of the Bahamas	1.49 (1.32-1.69)	0.64 (0.57-0.72)	2.38 (1.87-3.02)	0.57 (0.45-0.72)	-0.52 (-0.68--0.36)
HL	Cook Islands	0.02 (0.01-0.04)	0.12 (0.08-0.22)	0.03 (0.02-0.05)	0.15 (0.1-0.26)	1.38 (0.9-1.86)
HL	Czech Republic	329.62 (270.99-398.18)	2.91 (2.39-3.55)	275.7 (209.63-356.61)	2.35 (1.74-3.12)	-0.71 (-1.03--0.39)
HL	Democratic People's Republic of Korea	65.34 (37.72-97.19)	0.34 (0.2-0.51)	106.35 (72.21-184.75)	0.34 (0.23-0.59)	0.03 (-0.08-0.14)
HL	Democratic Republic of Sao Tome and Principe	0.01 (0-0.01)	0.01 (0-0.01)	0.01 (0.01-0.02)	0.01 (0-0.01)	-0.21 (-0.39--0.04)
HL	Democratic Republic of the Congo	96.69 (64.95-153.86)	0.41 (0.27-0.66)	238.94 (147.99-380.95)	0.4 (0.25-0.64)	0.02 (-0.03-0.07)
HL	Democratic Republic of Timor-Leste	1.84 (1.03-3.68)	0.35 (0.2-0.71)	3.16 (1.58-7.4)	0.27 (0.14-0.63)	-0.76 (-1.16--0.35)
HL	Democratic Socialist Republic of Sri Lanka	123.74 (71.36-161.79)	0.8 (0.46-1.04)	156.04 (95.33-275.49)	0.66 (0.4-1.16)	-1.17 (-1.46--0.88)
HL	Dominican Republic	4.84 (2.61-11.41)	0.08 (0.04-0.2)	8.85 (4.38-20.12)	0.08 (0.04-0.18)	-0.03 (-0.45-0.4)
HL	Eastern Republic of Uruguay	39.39 (32.83-47.55)	1.16 (0.96-1.4)	51.48 (42-61.93)	1.35 (1.08-1.64)	0.47 (0.11-0.83)
HL	Federal Democratic Republic of Ethiopia	791.77 (511.72-1067.64)	2.25 (1.4-3.04)	1102.7 (643.83-1748.11)	1.28 (0.75-1.96)	-2.22 (-2.36--2.08)
HL	Federal Democratic Republic of Nepal	133.62 (89.24-222.61)	0.88 (0.58-1.5)	180.18 (97.04-384.08)	0.61 (0.33-1.29)	-1.14 (-1.37--0.91)
HL	Federal Republic of Germany	2691 (2384.89-3023.82)	2.91 (2.57-3.27)	2180.04 (1806.18-2589.86)	2.05 (1.7-2.43)	-1.15 (-1.41--0.89)
HL	Federal Republic of Nigeria	1634.04 (568.21-2519.34)	2.42 (0.9-3.69)	3233.14 (995.12-5444.13)	1.85 (0.63-2.99)	-1.03 (-1.12--0.93)
HL	Federal Republic of Somalia	68.77 (41.02-97.31)	1.31 (0.78-1.86)	184.78 (105.28-289.11)	1.3 (0.76-1.96)	0.04 (-0.03-0.11)
HL	Federated States of Micronesia	0.1 (0.05-0.14)	0.13 (0.07-0.18)	0.1 (0.06-0.15)	0.1 (0.06-0.16)	-0.65 (-0.75--0.56)
HL	Federative Republic of Brazil	855.23 (816.19-897.47)	0.7 (0.67-0.73)	1225.67 (1135.97-1313.17)	0.52 (0.48-0.55)	-0.73 (-0.92--0.54)
HL	French Republic	1307.02 (1133.26-1507.02)	2.06 (1.78-2.37)	1573.34 (1293.94-1926.2)	2.15 (1.74-2.66)	0.68 (0.26-1.1)
HL	Gabonese Republic	2.88 (1.96-4.35)	0.41 (0.27-0.62)	5 (3.18-7.63)	0.34 (0.22-0.51)	-0.65 (-0.73--0.58)
HL	Georgia	123.28 (79.29-156.18)	2.13 (1.37-2.71)	77.69 (62.61-95.44)	1.93 (1.52-2.45)	-0.68 (-1.25--0.11)
HL	Global	54671.44 (45648.57-59832.16)	1.12 (0.93-1.23)	65182.01 (53167.43-77142.98)	0.79 (0.64-0.94)	-1.1 (-1.16--1.05)
HL	Grand Duchy of Luxembourg	7.85 (7.06-8.64)	1.79 (1.6-1.99)	10.07 (8.64-11.58)	1.36 (1.17-1.56)	-0.9 (-1.28--0.53)
HL	Greenland	0.5 (0.29-0.66)	0.92 (0.54-1.21)	0.36 (0.24-0.62)	0.56 (0.38-0.99)	-1.59 (-1.74--1.44)
HL	Grenada	0.47 (0.38-0.59)	0.62 (0.51-0.78)	0.44 (0.35-0.53)	0.39 (0.31-0.47)	-1.56 (-1.76--1.36)
HL	Guam	0.33 (0.2-0.49)	0.25 (0.16-0.37)	0.49 (0.23-0.66)	0.3 (0.14-0.41)	1.21 (0.87-1.54)
HL	Hashemite Kingdom of Jordan	11.86 (8.05-19.65)	0.44 (0.31-0.72)	61.48 (37.17-88.19)	0.51 (0.31-0.73)	0.89 (0.62-1.16)
HL	Hellenic Republic	837.74 (757.91-927.86)	7.3 (6.5-8.17)	624.12 (557.08-704.03)	5.44 (4.77-6.25)	-1.01 (-1.56--0.46)
HL	High SDI	20495.98 (19865.97-21164.32)	2.11 (2.04-2.17)	18581.14 (17508.59-19686.46)	1.42 (1.34-1.51)	-1.18 (-1.41--0.95)
HL	High-middle SDI	14471.76 (12560.17-15815.17)	1.33 (1.15-1.45)	14769.2 (13071.53-16109.33)	0.99 (0.88-1.08)	-0.93 (-1.03--0.82)
HL	Hungary	243.74 (213.2-279.06)	2.1 (1.82-2.41)	132.43 (105.16-168.43)	1.2 (0.95-1.51)	-2.32 (-2.51--2.12)
HL	Independent State of Papua New Guinea	3.6 (1.34-6.06)	0.11 (0.04-0.19)	7.73 (3.89-12.06)	0.09 (0.04-0.14)	-0.88 (-0.96--0.8)
HL	Independent State of Samoa	1.47 (0.87-2.84)	1.13 (0.69-2.23)	2.37 (1.35-4.62)	1.26 (0.73-2.48)	0.38 (0.29-0.47)
HL	Ireland	97.52 (85.21-112.94)	2.64 (2.3-3.06)	144.51 (118.96-172.6)	2.7 (2.22-3.23)	1.26 (0.75-1.76)
HL	Islamic Republic of Afghanistan	97.13 (58.94-180.27)	1.26 (0.75-2.35)	246.13 (158.24-394.68)	1.18 (0.76-1.88)	-0.22 (-0.34--0.1)
HL	Islamic Republic of Iran	183.86 (134.98-283.64)	0.41 (0.3-0.68)	734.1 (359.75-948.17)	0.86 (0.43-1.12)	3.35 (2.97-3.73)
HL	Islamic Republic of Mauritania	2.94 (1.65-4.05)	0.19 (0.11-0.26)	4.2 (2.67-7.02)	0.13 (0.08-0.2)	-1.79 (-1.93--1.66)
HL	Islamic Republic of Pakistan	1349.21 (850.25-2006.59)	1.56 (1-2.46)	3202.27 (2135.22-4724.35)	1.54 (1.04-2.34)	-0.23 (-0.4--0.06)
HL	Jamaica	9.64 (8.1-11.39)	0.44 (0.38-0.52)	12.08 (8.81-16.48)	0.39 (0.29-0.53)	-0.62 (-0.99--0.25)
HL	Japan	257.12 (245.33-268.54)	0.17 (0.17-0.18)	507.68 (466.06-541.98)	0.29 (0.27-0.32)	2.39 (2.14-2.64)
HL	Kingdom of Bahrain	4.83 (2.98-6.94)	1.4 (0.85-1.87)	22.03 (13.64-33.28)	1.47 (0.88-2.15)	0.31 (0.01-0.62)
HL	Kingdom of Belgium	304.65 (261.68-360.15)	2.7 (2.31-3.22)	291.75 (233.35-363.49)	2.28 (1.82-2.85)	-0.56 (-0.79--0.33)
HL	Kingdom of Bhutan	4.02 (2.27-6.9)	0.86 (0.5-1.54)	4.7 (2.32-10.79)	0.62 (0.31-1.41)	-1.22 (-1.32--1.13)
HL	Kingdom of Cambodia	35.49 (23.6-64.24)	0.5 (0.33-0.92)	55.26 (28.55-123.21)	0.35 (0.18-0.78)	-1.23 (-1.38--1.07)
HL	Kingdom of Denmark	99.01 (86.79-113.47)	1.67 (1.46-1.93)	87.69 (72.6-107.32)	1.25 (1.03-1.52)	-0.41 (-0.95-0.13)
HL	Kingdom of Eswatini	2.8 (1.8-4.69)	0.55 (0.37-0.92)	5.77 (3.57-9.12)	0.62 (0.39-0.95)	0.64 (0.23-1.05)
HL	Kingdom of Lesotho	4.83 (2.87-8.43)	0.44 (0.26-0.78)	9.92 (6.37-16.1)	0.65 (0.42-1.03)	1.88 (1.51-2.26)
HL	Kingdom of Morocco	189.59 (120.67-257.04)	0.91 (0.59-1.27)	314.09 (197.42-477.95)	0.85 (0.53-1.27)	-0.17 (-0.23--0.11)
HL	Kingdom of Norway	79.62 (72.85-86.89)	1.61 (1.46-1.77)	143.39 (128.17-159.55)	2.18 (1.95-2.42)	1.31 (0.94-1.68)
HL	Kingdom of Saudi Arabia	60.31 (19.76-100.75)	0.53 (0.18-0.87)	336.89 (81.68-609.55)	0.83 (0.21-1.45)	2 (1.64-2.36)
HL	Kingdom of Spain	1238.7 (1063.84-1444.59)	2.9 (2.47-3.4)	1304.67 (1057.68-1616.64)	2.43 (1.95-3.1)	-0.34 (-0.66--0.02)
HL	Kingdom of Sweden	125.06 (106.65-145.3)	1.22 (1.02-1.45)	151.49 (122.71-185.61)	1.26 (1.01-1.59)	0.28 (-0.24-0.8)

Cause	Location	1990		2021		EAPC No. (95% CI)
		Incident cases (95% UI)	ASIR per 100,000 No. (95% UI)	Incident cases (95% UI)	ASIR per 100,000 No. (95% UI)	
HL	Kingdom of Thailand	156.47 (106.65-270.97)	0.29 (0.2-0.53)	289.18 (176.17-591.05)	0.4 (0.25-0.78)	0.39 (0.15-0.63)
HL	Kingdom of the Netherlands	390.42 (341.87-448.9)	2.35 (2.04-2.72)	462.65 (381.57-559.98)	2.45 (1.98-3.02)	0.8 (0.51-1.09)
HL	Kingdom of Tonga	0.06 (0.03-0.09)	0.08 (0.04-0.12)	0.07 (0.04-0.12)	0.08 (0.04-0.12)	-0.03 (-0.09-0.04)
HL	Kyrgyz Republic	44.11 (34.05-56.31)	1.12 (0.87-1.41)	33.79 (24.09-45.53)	0.51 (0.37-0.69)	-0.58 (-1.6-0.45)
HL	Lao People's Democratic Republic	14.91 (9.7-27.25)	0.5 (0.33-0.93)	19.05 (9.9-43.76)	0.29 (0.16-0.66)	-1.91 (-2.01--1.82)
HL	Lebanese Republic	48.91 (27.75-66.39)	1.86 (1.07-2.52)	126.07 (75.94-171.7)	2.13 (1.28-2.92)	0.75 (0.62-0.87)
HL	Low SDI	4161.95 (2423.49-5218.62)	1.12 (0.65-1.44)	7494.47 (4361.26-10262.42)	0.83 (0.49-1.15)	-1.12 (-1.19--1.05)
HL	Low-middle SDI	6959.4 (4556.99-9351.94)	0.75 (0.49-1.04)	11300.51 (8143.82-16508.39)	0.62 (0.45-0.91)	-0.69 (-0.74--0.63)
HL	Malaysia	76.43 (39.3-105.7)	0.54 (0.28-0.73)	190.34 (109.54-240.7)	0.58 (0.33-0.73)	0.07 (-0.14-0.28)
HL	Middle SDI	8508.28 (5675.85-10032.08)	0.6 (0.4-0.71)	12968.21 (9236.49-15912.52)	0.51 (0.36-0.62)	-0.59 (-0.74--0.43)
HL	Mongolia	7.86 (4.27-15.54)	0.47 (0.26-0.97)	18.69 (11.24-27.27)	0.59 (0.35-0.87)	0.99 (0.7-1.27)
HL	Montenegro	20.11 (12.65-27.91)	3.09 (1.95-4.28)	20.09 (13.3-28.5)	2.99 (1.98-4.24)	0.33 (0.15-0.52)
HL	New Zealand	32.85 (27.92-39.07)	0.87 (0.74-1.04)	53.55 (44.85-63.4)	0.85 (0.7-1.01)	-0.15 (-1.06-0.77)
HL	North Macedonia	41.3 (25.68-58.75)	2.05 (1.28-2.91)	48.05 (31.56-79.07)	1.91 (1.25-3.19)	-0.14 (-0.39-0.11)
HL	Northern Mariana Islands	0.02 (0.01-0.03)	0.05 (0.03-0.07)	0.03 (0.01-0.05)	0.06 (0.03-0.09)	0.61 (0.35-0.87)
HL	Palestine	18.94 (10.74-26.65)	1.37 (0.77-1.91)	56.78 (32.26-76.41)	1.3 (0.73-1.73)	-0.05 (-0.22-0.12)
HL	People's Democratic Republic of Algeria	293.73 (181.97-399.28)	1.43 (0.9-1.99)	625.51 (396.97-889.57)	1.48 (0.95-2.09)	0.24 (0.16-0.32)
HL	People's Republic of Bangladesh	871.74 (602.47-1417.8)	1.03 (0.7-1.74)	1098.16 (610.24-2310.73)	0.67 (0.38-1.39)	-1.47 (-1.6--1.34)
HL	People's Republic of China	4745.97 (2015.45-6560.88)	0.48 (0.2-0.67)	4211.06 (2542-5541.5)	0.23 (0.14-0.3)	-2.73 (-2.99--2.46)
HL	Plurinational State of Bolivia	45.28 (26.36-60.92)	0.99 (0.6-1.31)	69.82 (39.98-111.95)	0.66 (0.38-1.03)	-1.37 (-1.46--1.29)
HL	Portuguese Republic	197.74 (173.32-226.79)	1.79 (1.56-2.07)	216.14 (175.2-265.17)	1.81 (1.43-2.28)	-0.02 (-0.34-0.3)
HL	Principality of Andorra	1.16 (0.68-1.76)	1.84 (1.09-2.79)	2.07 (1.18-3.19)	2.18 (1.25-3.31)	0.69 (0.3-1.07)
HL	Principality of Monaco	2.43 (1.28-3.46)	7.07 (3.73-10.2)	3.56 (2.13-5.97)	9.4 (5.39-15.7)	0.91 (0.74-1.08)
HL	Puerto Rico	61.75 (53.35-71.88)	1.68 (1.45-1.95)	57.25 (45.45-72.21)	1.55 (1.22-1.96)	-0.19 (-0.48-0.11)
HL	Republic of Albania	34.25 (21.68-49.09)	1.13 (0.72-1.67)	34.42 (22.13-53.71)	1.16 (0.73-1.81)	0.57 (0.19-0.95)
HL	Republic of Angola	29.73 (20.2-45.16)	0.46 (0.31-0.73)	76.29 (48.76-119.43)	0.37 (0.23-0.57)	-0.75 (-0.82--0.68)
HL	Republic of Armenia	22.63 (18.08-26.53)	0.68 (0.55-0.8)	21.4 (17.09-25.58)	0.65 (0.52-0.79)	0.07 (-0.28-0.43)
HL	Republic of Austria	263.32 (230.94-297.97)	2.88 (2.51-3.27)	223.38 (183.07-267.41)	2.13 (1.7-2.59)	-0.78 (-1.07--0.49)
HL	Republic of Azerbaijan	76.56 (48.49-116.85)	1.1 (0.7-1.66)	118.64 (71.02-183.13)	1.08 (0.65-1.66)	-0.14 (-0.31-0.03)
HL	Republic of Belarus	206.13 (168.91-250.23)	1.85 (1.51-2.27)	295 (225.66-384.18)	2.84 (2.13-3.74)	1.56 (1.36-1.76)
HL	Republic of Benin	6.62 (3.49-9.23)	0.2 (0.11-0.27)	11.99 (7.6-19.32)	0.13 (0.08-0.2)	-1.72 (-1.9--1.54)
HL	Republic of Botswana	4.83 (2.95-8.37)	0.54 (0.34-0.91)	8.91 (4.97-15.51)	0.41 (0.24-0.68)	-0.87 (-1.02--0.71)
HL	Republic of Bulgaria	221.13 (184.11-264.57)	2.32 (1.91-2.77)	140.56 (108.4-181.16)	1.79 (1.39-2.3)	-0.72 (-0.97--0.47)
HL	Republic of Burundi	51.65 (30.22-71.22)	1.21 (0.72-1.66)	101.04 (52.14-157.72)	1.01 (0.52-1.53)	-0.72 (-0.79--0.65)
HL	Republic of Cabo Verde	0.06 (0.02-0.1)	0.02 (0.01-0.03)	0.07 (0.04-0.1)	0.01 (0.01-0.02)	-1.16 (-1.56--0.76)
HL	Republic of Cameroon	15.45 (8.05-21.46)	0.21 (0.11-0.29)	32.99 (20.3-51.2)	0.14 (0.09-0.22)	-1.58 (-1.76--1.41)
HL	Republic of Chad	8.29 (4.5-11.98)	0.19 (0.11-0.27)	18.29 (11.56-30.03)	0.17 (0.11-0.26)	-0.71 (-0.93--0.49)
HL	Republic of Chile	55.71 (46.69-67.43)	0.45 (0.38-0.54)	232.83 (188.51-285.48)	1.13 (0.9-1.4)	3.73 (2.94-4.53)
HL	Republic of Colombia	224.37 (200.26-251.07)	0.83 (0.75-0.92)	433.11 (346.86-541.58)	0.82 (0.66-1.03)	-0.28 (-0.48--0.07)
HL	Republic of Costa Rica	54.21 (46.43-62.67)	2 (1.72-2.27)	77.57 (63.54-93.25)	1.5 (1.23-1.81)	-1.02 (-1.35--0.69)
HL	Republic of C 么 te d'Ivoire	48.85 (29.01-73.62)	0.58 (0.37-0.9)	112.54 (65.01-171.27)	0.51 (0.32-0.78)	-0.34 (-0.44--0.25)
HL	Republic of Croatia	162.97 (143.07-187.67)	2.96 (2.59-3.41)	102.72 (84.53-122.65)	1.92 (1.56-2.32)	-1.35 (-1.71--1)
HL	Republic of Cuba	267.23 (218.89-316.03)	2.29 (1.89-2.72)	272.06 (224.22-331.15)	2.04 (1.66-2.49)	-0.76 (-0.94--0.57)
HL	Republic of Cyprus	13.16 (8.7-20.26)	1.68 (1.11-2.55)	34.8 (22.53-55.25)	2.2 (1.44-3.49)	1.35 (0.91-1.78)
HL	Republic of Djibouti	2.7 (1.48-4.15)	0.88 (0.5-1.34)	9.55 (5.35-15.64)	0.88 (0.51-1.41)	-0.02 (-0.23-0.2)
HL	Republic of Ecuador	30.59 (25.58-36.79)	0.42 (0.35-0.49)	131.42 (99.74-170.41)	0.75 (0.57-0.96)	2.37 (1.84-2.91)
HL	Republic of El Salvador	25.09 (16.49-35.15)	0.62 (0.4-0.89)	42.36 (27.69-58.08)	0.66 (0.43-0.9)	0.27 (0.13-0.4)
HL	Republic of Equatorial Guinea	1.36 (0.9-2.15)	0.5 (0.33-0.79)	3.51 (2.01-5.73)	0.31 (0.19-0.51)	-1.78 (-2.1--1.45)
HL	Republic of Estonia	32.56 (25.56-41.15)	1.95 (1.51-2.5)	27.96 (21.03-35.5)	2.05 (1.51-2.62)	0.31 (-0.15-0.76)
HL	Republic of Fiji	1.82 (1.04-4.02)	0.28 (0.16-0.63)	3.47 (2.08-5.55)	0.39 (0.23-0.62)	1.75 (1.34-2.16)
HL	Republic of Finland	113.44 (100.38-131.86)	2 (1.76-2.35)	126.87 (105.13-149.04)	2.01 (1.64-2.46)	0.83 (0.27-1.39)
HL	Republic of Ghana	3.8 (1.24-6.18)	0.04 (0.01-0.06)	5.73 (2.86-8.41)	0.02 (0.01-0.03)	-2.33 (-2.67--1.98)
HL	Republic of Guatemala	25.22 (20.9-28.37)	0.46 (0.4-0.51)	37.81 (31.51-44.35)	0.27 (0.23-0.32)	-1.71 (-1.87--1.55)
HL	Republic of Guinea	29.9 (18.8-43.1)	0.62 (0.42-0.97)	58.19 (36.2-91.77)	0.56 (0.37-0.88)	-0.25 (-0.31--0.19)
HL	Republic of Guinea-Bissau	2.15 (1.15-3.17)	0.31 (0.17-0.44)	2.9 (1.96-4.51)	0.2 (0.14-0.31)	-1.5 (-1.61--1.38)
HL	Republic of Guyana	3.59 (2.95-4.44)	0.61 (0.52-0.73)	3.98 (3.04-5.18)	0.53 (0.4-0.69)	-0.31 (-0.55--0.06)
HL	Republic of Haiti	41.61 (27.71-63.02)	0.9 (0.59-1.41)	83.34 (43.5-127.93)	0.79 (0.41-1.22)	-0.31 (-0.39--0.23)
HL	Republic of Honduras	10.75 (6.84-14.2)	0.33 (0.21-0.44)	23.46 (14.43-35.02)	0.3 (0.18-0.43)	-0.4 (-0.5--0.29)
HL	Republic of Iceland	6.46 (5.52-7.8)	2.42 (2.07-2.93)	8.39 (6.75-10.25)	2.22 (1.77-2.73)	0.13 (-0.25-0.51)
HL	Republic of India	5078.77 (2947.4-6373.42)	0.72 (0.41-0.9)	7455.17 (5107.64-10809.06)	0.53 (0.36-0.77)	-1.06 (-1.16--0.96)
HL	Republic of Indonesia	531.58 (363.89-994.12)	0.35 (0.24-0.67)	766.71 (447.91-1649.22)	0.28 (0.16-0.59)	-0.82 (-0.87--0.78)
HL	Republic of Iraq	136.07 (66.64-193.79)	0.98 (0.49-1.37)	359.94 (177.86-549.26)	0.95 (0.47-1.42)	-0.24 (-0.33--0.16)
HL	Republic of Italy	2143.62 (1972.22-2372.18)	3.32 (3.03-3.71)	2003.71 (1798.21-2201.79)	2.9 (2.57-3.22)	0.12 (-0.3-0.55)
HL	Republic of Kazakhstan	120.33 (97.16-173.68)	0.75 (0.61-1.08)	141.9 (116.54-172.29)	0.74 (0.61-0.9)	-0.18 (-0.48-0.11)
HL	Republic of Kenya	76.9 (46.81-113.63)	0.47 (0.28-0.71)	204.58 (117.44-310)	0.5 (0.29-0.74)	0.52 (0.35-0.69)
HL	Republic of Kiribati	0.04 (0.02-0.06)	0.06 (0.02-0.09)	0.06 (0.02-0.1)	0.06 (0.02-0.09)	-0.14 (-0.19--0.09)
HL	Republic of Korea	58.83 (42.51-102.27)	0.15 (0.11-0.26)	132.48 (77.34-185.89)	0.21 (0.13-0.29)	2.02 (1.65-2.39)
HL	Republic of Latvia	48.58 (39.54-58.67)	1.71 (1.38-2.07)	36.96 (27.81-47.25)	1.77 (1.33-2.29)	-0.16 (-0.39-0.07)
HL	Republic of Liberia	3.57 (1.8-5.09)	0.2 (0.1-0.28)	6.12 (3.79-9.52)	0.15 (0.09-0.22)	-1.17 (-1.36--0.97)
HL	Republic of Lithuania	76.67 (65.35-88.34)	1.97 (1.66-2.28)	51.17 (41.29-62.33)	1.67 (1.34-2.05)	-1 (-1.32--0.68)
HL	Republic of Madagascar	85.88 (55.53-115.87)	0.93 (0.6-1.25)	195.51 (112.98-303.61)	0.83 (0.48-1.27)	-0.34 (-0.38--0.29)
HL	Republic of Malawi	47.42 (26.71-67.04)	0.6 (0.36-0.84)	83.76 (46.39-123.71)	0.53 (0.31-0.76)	-0.48 (-0.62--0.35)
HL	Republic of Maldives	0.77 (0.5-1.18)	0.5 (0.35-0.75)	2.09 (1.28-3.65)	0.4 (0.25-0.65)	-0.55 (-0.66--0.43)
HL	Republic of Mali	56.99 (33.52-80.52)	0.87 (0.58-1.38)	117.36 (73.75-186.49)	0.66 (0.43-1.16)	-0.76 (-0.88--0.65)
HL	Republic of Malta	9.71 (8.22-11.48)	2.49 (2.1-2.97)	13.66 (11.23-16.91)	2.77 (2.24-3.43)	0.97 (0.66-1.27)
HL	Republic of Mauritius	7.01 (6.38-7.64)	0.7 (0.65-0.76)	13.99 (12.4-15.69)	0.94 (0.82-1.05)	-1.08 (-2.17-0.01)
HL	Republic of Moldova	123.52 (109.35-139.6)	2.76 (2.45-3.13)	80.13 (66.73-95.59)	1.93 (1.6-2.33)	-1.26 (-1.61--0.9)
HL	Republic of Mozambique	120.86 (66.47-171.18)	1.15 (0.63-1.65)	273.59 (136.4-426.22)	1.18 (0.6-1.78)	0.49 (0.34-0.64)
HL	Republic of Namibia	6.66 (3.93-9.55)	0.66 (0.42-0.97)	14.35 (8.3-21.85)	0.68 (0.41-1.01)	0.05 (-0.1-0.21)
HL	Republic of Nauru	0.01 (0.01-0.02)	0.13 (0.07-0.19)	0.01 (0.01-0.02)	0.12 (0.06-0.19)	-0.31 (-0.43--0.2)
HL	Republic of Nicaragua	11.4 (7.5-17.39)	0.42 (0.27-0.65)	22.75 (15.15-32.99)	0.37 (0.24-0.52)	-0.19 (-0.32--0.07)
HL	Republic of Niue	0 (0-0)	0.1 (0.05-0.15)	0 (0-0)	0.14 (0.08-0.18)	-0.04 (-0.42-0.33)

Cause	Location	1990		2021		EAPC No. (95% CI)
		Incident cases (95% UI)	ASIR per 100,000 No. (95% UI)	Incident cases (95% UI)	ASIR per 100,000 No. (95% UI)	
HL	Republic of Palau	0.01 (0-0.01)	0.04 (0.02-0.08)	0.01 (0-0.01)	0.05 (0.02-0.08)	0.46 (0.28-0.64)
HL	Republic of Panama	9.41 (8.49-10.6)	0.47 (0.43-0.53)	23.24 (18.65-28.58)	0.53 (0.43-0.65)	0.42 (0.29-0.54)
HL	Republic of Paraguay	14.02 (8.85-22.3)	0.44 (0.27-0.69)	32.9 (21.56-47.59)	0.47 (0.31-0.68)	0.44 (0.36-0.51)
HL	Republic of Peru	67.24 (46.82-101.93)	0.4 (0.28-0.59)	160.48 (99.52-226.13)	0.44 (0.27-0.62)	0.47 (0.27-0.68)
HL	Republic of Poland	894.04 (850.29-942.49)	2.2 (2.09-2.33)	571.36 (516.23-629.76)	1.37 (1.21-1.53)	-2.14 (-2.47--1.81)
HL	Republic of Rwanda	78.21 (49.49-106.3)	1.42 (0.9-1.87)	100.73 (54.93-162.19)	0.9 (0.5-1.39)	-2.11 (-2.34--1.89)
HL	Republic of San Marino	1.58 (0.94-2.26)	5.83 (3.47-8.38)	1.71 (0.96-2.83)	4.99 (2.74-8.28)	0.1 (-0.22-0.42)
HL	Republic of Senegal	11.59 (5.82-16.52)	0.21 (0.11-0.29)	19.07 (12.27-31.2)	0.15 (0.1-0.24)	-1.1 (-1.21--0.99)
HL	Republic of Serbia	181.2 (110.46-259.53)	1.75 (1.08-2.52)	200.88 (132.62-298.36)	1.78 (1.17-2.71)	0.04 (-0.08-0.17)
HL	Republic of Seychelles	0.55 (0.38-0.76)	0.81 (0.56-1.1)	0.77 (0.56-1.12)	0.69 (0.49-1.01)	-0.37 (-0.53--0.2)
HL	Republic of Sierra Leone	5.5 (2.57-7.96)	0.18 (0.09-0.25)	9.11 (5.74-14.14)	0.14 (0.09-0.21)	-0.92 (-1--0.84)
HL	Republic of Singapore	8.76 (7.47-10.16)	0.3 (0.26-0.34)	21.81 (17.88-26.49)	0.35 (0.28-0.45)	1.96 (1.42-2.5)
HL	Republic of Slovenia	47.25 (40.21-54.83)	2.2 (1.86-2.56)	40.31 (30.48-53.49)	1.75 (1.33-2.32)	-0.11 (-0.53-0.3)
HL	Republic of South Africa	116.67 (82.91-165.22)	0.37 (0.26-0.52)	224.07 (134.87-279.3)	0.4 (0.24-0.49)	0.32 (0.12-0.53)
HL	Republic of South Sudan	41.33 (25.11-58.91)	0.94 (0.56-1.36)	79 (44.63-119.89)	1.05 (0.6-1.57)	0.28 (0.03-0.54)
HL	Republic of Sudan	100.58 (63.81-186.2)	0.69 (0.43-1.34)	244.46 (140.42-408.39)	0.67 (0.39-1.13)	0.05 (-0.01-0.11)
HL	Republic of Suriname	1.98 (1.3-2.94)	0.59 (0.4-0.91)	3.7 (2.16-5.16)	0.61 (0.35-0.84)	0.14 (-0.02-0.3)
HL	Republic of Tajikistan	8.22 (5.36-12.18)	0.19 (0.12-0.28)	14.64 (9.01-23.02)	0.16 (0.1-0.24)	-1.13 (-1.32--0.95)
HL	Republic of the Congo	7.35 (5.14-11.23)	0.48 (0.34-0.72)	16.14 (10.41-24.38)	0.38 (0.26-0.58)	-0.83 (-0.95--0.72)
HL	Republic of the Gambia	3.41 (1.96-5.18)	0.47 (0.3-0.76)	9.04 (5.8-15.6)	0.46 (0.31-0.81)	-0.32 (-0.56--0.09)
HL	Republic of the Marshall Islands	0.04 (0.02-0.05)	0.12 (0.07-0.16)	0.06 (0.03-0.08)	0.11 (0.06-0.16)	-0.36 (-0.42--0.3)
HL	Republic of the Niger	11.71 (6.02-16.95)	0.22 (0.12-0.31)	21.92 (12.56-37.64)	0.15 (0.09-0.24)	-1.63 (-1.79--1.46)
HL	Republic of the Philippines	87.39 (57.01-117.74)	0.17 (0.11-0.24)	188.79 (117.51-240.06)	0.18 (0.11-0.22)	0.14 (0.01-0.27)
HL	Republic of the Union of Myanmar	166.42 (103.23-309.12)	0.49 (0.31-0.9)	157.68 (86.48-352.01)	0.29 (0.16-0.63)	-2.11 (-2.26--1.96)
HL	Republic of Trinidad and Tobago	6.21 (5.54-7.05)	0.59 (0.53-0.66)	7.76 (5.82-10.01)	0.5 (0.38-0.64)	-0.73 (-0.95--0.5)
HL	Republic of Tunisia	112.6 (74.58-166.09)	1.54 (1.04-2.39)	230.75 (147.73-359.5)	1.89 (1.21-2.93)	0.62 (0.57-0.67)
HL	Republic of Turkey	421.85 (294.66-731.08)	0.87 (0.61-1.54)	750.61 (515.57-1251.91)	0.84 (0.58-1.42)	0.1 (-0.05-0.24)
HL	Republic of Uganda	159.5 (96.68-233.71)	1.31 (0.81-1.97)	475.36 (274.28-694.07)	1.4 (0.84-2.02)	-0.17 (-0.37-0.04)
HL	Republic of Uzbekistan	130.28 (97.25-175.46)	0.73 (0.54-0.99)	216.76 (158.55-288.96)	0.64 (0.47-0.85)	-0.43 (-0.57--0.29)
HL	Republic of Vanuatu	0.12 (0.05-0.19)	0.11 (0.05-0.18)	0.25 (0.14-0.36)	0.1 (0.05-0.14)	-0.57 (-0.67--0.48)
HL	Republic of Yemen	48.41 (30.27-80.84)	0.61 (0.38-1.12)	147.94 (84.77-226.97)	0.62 (0.37-0.98)	0.11 (-0.02-0.24)
HL	Republic of Zambia	63.58 (40.63-85.21)	1.08 (0.68-1.48)	143.51 (83.61-214.46)	0.93 (0.56-1.35)	-0.7 (-0.85--0.56)
HL	Republic of Zimbabwe	38.26 (21.36-58.47)	0.57 (0.35-0.9)	102.45 (61.71-147.7)	0.84 (0.52-1.22)	1.87 (1.36-2.38)
HL	Romania	399.78 (339.6-465.4)	1.62 (1.37-1.9)	286.51 (214.11-365.5)	1.31 (0.97-1.69)	-0.99 (-1.19--0.79)
HL	Russian Federation	3900.71 (3752.19-4071.82)	2.48 (2.39-2.6)	3556.78 (3258.91-3845.68)	2.22 (2.05-2.39)	-0.51 (-0.75--0.27)
HL	Saint Kitts and Nevis	0.01 (0.01-0.01)	0.02 (0.02-0.03)	0.07 (0.06-0.09)	0.11 (0.09-0.13)	4.33 (2.42-6.27)
HL	Saint Lucia	1.14 (1.01-1.32)	1.04 (0.93-1.19)	1.75 (1.41-2.16)	0.86 (0.69-1.08)	-0.76 (-0.88--0.63)
HL	Saint Vincent and the Grenadines	0.87 (0.77-0.99)	0.94 (0.83-1.08)	0.89 (0.76-1.06)	0.71 (0.6-0.84)	-1.05 (-1.21--0.89)
HL	Slovak Republic	108.29 (67.14-147.69)	1.94 (1.2-2.64)	118.23 (76.65-175.43)	1.95 (1.25-2.89)	0.26 (0.13-0.39)
HL	Socialist Republic of Viet Nam	302.74 (193.53-482.3)	0.55 (0.36-0.89)	743.56 (469.9-1144.83)	0.73 (0.46-1.12)	1.06 (0.94-1.18)
HL	Solomon Islands	0.28 (0.1-0.45)	0.12 (0.04-0.2)	0.61 (0.34-0.93)	0.11 (0.06-0.17)	-0.31 (-0.42--0.2)
HL	State of Eritrea	29.78 (18.14-40.91)	1.23 (0.75-1.65)	60.74 (34.98-88.91)	1.14 (0.67-1.64)	-0.13 (-0.18--0.07)
HL	State of Israel	93.61 (81.4-106.9)	1.93 (1.68-2.2)	165.98 (134.42-201.38)	1.71 (1.38-2.08)	-0.05 (-0.49-0.39)
HL	State of Kuwait	27.25 (23.15-32.06)	1.75 (1.52-2.03)	32.5 (25.23-40.13)	0.68 (0.53-0.84)	0.47 (-1.48-2.45)
HL	State of Libya	64.33 (40.12-95.63)	1.95 (1.25-3.09)	171.53 (102.5-246.77)	2.43 (1.49-3.48)	1.29 (1.08-1.5)
HL	State of Qatar	1.75 (1.01-3.28)	0.57 (0.36-1.03)	21.71 (11.59-34.05)	0.74 (0.41-1.12)	1.8 (1.4-2.2)
HL	Sultanate of Oman	14.08 (6.96-21.11)	1.01 (0.52-1.5)	47.84 (22.22-70.38)	1.07 (0.52-1.54)	0.83 (0.58-1.09)
HL	Swiss Confederation	176.1 (152.11-209.16)	2.32 (1.98-2.78)	117.58 (95.2-146)	1.17 (0.93-1.48)	-2.28 (-2.89--1.67)
HL	Syrian Arab Republic	3.39 (2.21-4.55)	0.04 (0.02-0.05)	7.14 (3.63-10.18)	0.05 (0.03-0.07)	1.2 (0.85-1.55)
HL	Taiwan (Province of China)	74.85 (65.47-84.91)	0.37 (0.33-0.42)	117.01 (98.6-137.89)	0.38 (0.32-0.47)	-0.49 (-0.85--0.13)
HL	Togolese Republic	4.94 (2.62-6.79)	0.2 (0.11-0.26)	9.41 (6.11-13.92)	0.15 (0.1-0.21)	-1.16 (-1.29--1.02)
HL	Tokelau	0 (0-0)	0.11 (0.06-0.17)	0 (0-0)	0.15 (0.09-0.23)	-0.19 (-0.65-0.27)
HL	Turkmenistan	22.1 (18.95-26.42)	0.69 (0.61-0.81)	88.18 (67.05-117.08)	1.7 (1.3-2.24)	3.52 (3.06-3.98)
HL	Tuvalu	0.01 (0.01-0.02)	0.14 (0.07-0.19)	0.01 (0.01-0.02)	0.1 (0.06-0.14)	-0.99 (-1.07--0.9)
HL	Ukraine	833.22 (645.02-1123.46)	1.52 (1.18-2.02)	723.22 (510.82-1012.98)	1.56 (1.1-2.17)	-0.28 (-0.62-0.06)
HL	Union of the Comoros	3.74 (1.63-5.57)	1.05 (0.52-1.54)	6.46 (3.75-9.75)	0.95 (0.55-1.42)	-0.68 (-1.01--0.34)
HL	United Arab Emirates	10.96 (6.68-15.78)	0.88 (0.52-1.24)	53.2 (32.62-84.33)	0.71 (0.43-0.98)	0.18 (-0.05-0.41)
HL	United Kingdom of Great Britain and Northern Ireland	1775.6 (1722.93-1826.8)	2.74 (2.65-2.83)	2095.69 (2014.47-2183.1)	2.74 (2.62-2.86)	0.63 (0.29-0.98)
HL	United Mexican States	700.3 (677.64-726.72)	1.13 (1.09-1.17)	1158.5 (1029.65-1287.25)	0.88 (0.78-0.98)	-0.75 (-1.04--0.46)
HL	United Republic of Tanzania	205.78 (126.83-289.7)	1.02 (0.63-1.44)	414.76 (233.5-634.44)	0.86 (0.48-1.29)	-0.56 (-0.61--0.51)
HL	United States of America	9659.55 (9395.5-9916.41)	3.45 (3.35-3.54)	6837.47 (6486.49-7134.34)	1.68 (1.61-1.76)	-2.36 (-2.7--2.02)
HL	United States Virgin Islands	0.22 (0.14-0.39)	0.22 (0.14-0.39)	0.28 (0.15-0.41)	0.35 (0.18-0.56)	2.45 (2.12-2.77)

Table S5. The prevalence cases and age standardized prevalence of HL caused by different reasons from 1990 to 2021.

Cause	Location	1990		2021		EAPC No. (95% CI)
		Prevalence cases (95% UI)	ASPR per 100,000 No. (95% UI)	Prevalence cases (95% UI)	ASPR per 100,000 No. (95% UI)	
HL	American Samoa	0.14 (0.08-0.25)	0.31 (0.18-0.52)	0.24 (0.13-0.38)	0.53 (0.28-0.86)	1.88 (1.6-2.17)
HL	Antigua and Barbuda	0.55 (0.47-0.67)	0.91 (0.79-1.09)	0.87 (0.75-1.01)	0.89 (0.76-1.05)	0.24 (-0.09-0.57)
HL	Arab Republic of Egypt	229.44 (106.92-577.31)	0.47 (0.22-1.19)	786.01 (373.63-1885.25)	0.77 (0.37-1.86)	1.91 (1.59-2.23)
HL	Argentine Republic	1888.57 (1555.58-2325.84)	5.82 (4.79-7.17)	3274.2 (2664.24-4021.02)	6.83 (5.53-8.42)	0.78 (0.42-1.13)
HL	Australia	2228.49 (1921.39-2590.97)	12.47 (10.71-14.58)	4588.6 (3683.76-5698.27)	16.12 (12.86-20.04)	1.22 (0.75-1.69)
HL	Barbados	8.61 (7.65-9.71)	3.16 (2.81-3.55)	11.67 (8.93-15.16)	3.7 (2.82-4.83)	0.19 (-0.13-0.51)
HL	Belize	3.85 (3.12-5.55)	2.3 (1.88-3.31)	12.45 (10.38-14.87)	2.74 (2.31-3.25)	0.49 (0.13-0.85)
HL	Bermuda	5.62 (4.69-6.7)	8.54 (7.09-10.26)	8.14 (6.29-10.54)	12.85 (9.81-16.72)	1.41 (1.04-1.78)
HL	Bolivarian Republic of Venezuela	357.57 (313.2-411.15)	2.11 (1.85-2.4)	1231.84 (882.38-1643.17)	4.59 (3.27-6.11)	2.75 (2.53-2.98)
HL	Bosnia and Herzegovina	276.44 (171.66-370.83)	5.7 (3.55-7.64)	267.44 (175.06-432.28)	7.83 (5.09-12.77)	1.23 (0.9-1.56)
HL	Brunei Darussalam	4.7 (3.18-8.57)	2.58 (1.76-4.74)	14.65 (9.7-20.35)	3.19 (2.1-4.38)	1.56 (1.28-1.84)
HL	Burkina Faso	39.62 (19.62-57.24)	0.51 (0.25-0.71)	71.82 (46.61-114.45)	0.37 (0.24-0.57)	-1.21 (-1.4--1.02)
HL	Canada	6315.2 (5417.95-7402.38)	21.56 (18.37-25.49)	6691.88 (5345.1-8171.54)	17.02 (13.44-20.89)	-0.66 (-1.03--0.3)
HL	Central African Republic	23.99 (16.76-36.75)	1.12 (0.79-1.7)	48.52 (31.85-71.9)	1.08 (0.71-1.61)	-0.12 (-0.19--0.05)
HL	Commonwealth of Dominica	1.2 (0.71-1.87)	1.74 (1.04-2.69)	1.59 (0.99-2.57)	2.22 (1.37-3.59)	0.75 (0.55-0.94)
HL	Commonwealth of the Bahamas	6.04 (5.28-6.88)	2.29 (2.01-2.58)	11.83 (9.18-15.05)	2.8 (2.18-3.57)	0.68 (0.45-0.9)
HL	Cook Islands	0.09 (0.06-0.18)	0.52 (0.34-0.99)	0.18 (0.11-0.3)	1.03 (0.63-1.72)	2.69 (2.16-3.22)
HL	Czech Republic	1754.36 (1421.54-2166.65)	16.59 (13.52-20.67)	1910.89 (1437.62-2486.48)	17.57 (12.96-23.5)	0.06 (-0.4-0.52)
HL	Democratic People's Republic of Korea	259.02 (147.13-386.23)	1.26 (0.72-1.87)	519.22 (340.22-901.77)	1.73 (1.14-3.01)	1.08 (0.87-1.3)
HL	Democratic Republic of Sao Tome and Principe	0.02 (0.01-0.03)	0.02 (0.01-0.03)	0.06 (0.03-0.09)	0.03 (0.01-0.04)	1.03 (0.85-1.21)
HL	Democratic Republic of the Congo	250.33 (170.63-394.58)	0.85 (0.57-1.35)	751.45 (458.95-1251.46)	1 (0.62-1.61)	0.64 (0.52-0.76)
HL	Democratic Republic of Timor-Leste	5.68 (3.18-10.88)	0.84 (0.47-1.68)	11.84 (5.83-28.05)	0.87 (0.43-2.08)	0.34 (-0.25-0.94)
HL	Democratic Socialist Republic of Sri Lanka	555.64 (316.35-736.9)	3.17 (1.82-4.16)	989.32 (599.52-1744.9)	4.29 (2.61-7.52)	0.39 (0.08-0.7)
HL	Dominican Republic	16.54 (8.96-37.34)	0.24 (0.13-0.55)	38.74 (19.08-85.24)	0.34 (0.17-0.73)	1.32 (0.8-1.85)
HL	Eastern Republic of Uruguay	155.08 (128.81-189.26)	4.85 (4.02-5.94)	301.5 (240.73-371.11)	8.57 (6.8-10.65)	1.86 (1.55-2.17)
HL	Federal Democratic Republic of Ethiopia	2091.27 (1367.85-2830.76)	4.83 (3.08-6.48)	4063.45 (2276.9-6790.33)	3.8 (2.15-6.11)	-1.16 (-1.35--0.97)
HL	Federal Democratic Republic of Nepal	383.16 (259.04-621.68)	2.12 (1.41-3.54)	717.98 (370.1-1546.41)	2.21 (1.15-4.76)	0.24 (-0.01-0.49)
HL	Federal Republic of Germany	16893.64 (14864.56-19125.49)	19.5 (17.05-22.1)	15234.52 (12686.56-18093.77)	15.49 (12.85-18.44)	-0.77 (-1.07--0.47)
HL	Federal Republic of Nigeria	4227.47 (1381.39-6690.91)	5.24 (1.82-8.14)	10257.59 (2973.31-17863.79)	4.71 (1.45-7.97)	-0.51 (-0.65--0.37)
HL	Federal Republic of Somalia	190.76 (112.94-271.45)	2.84 (1.7-4.1)	544.25 (310.03-860.26)	3 (1.73-4.69)	0.23 (0.15-0.31)
HL	Federated States of Micronesia	0.31 (0.17-0.44)	0.35 (0.19-0.49)	0.39 (0.21-0.59)	0.37 (0.2-0.56)	0.19 (0.08-0.3)
HL	Federative Republic of Brazil	2887.19 (2729.46-3043.03)	2.05 (1.94-2.15)	5945.17 (5469.38-6383.47)	2.56 (2.35-2.75)	1.01 (0.81-1.2)
HL	French Republic	8109.11 (6918.68-9388.82)	13.37 (11.36-15.54)	11364.15 (9303.17-14019.31)	16.49 (13.29-20.48)	1.23 (0.74-1.72)
HL	Gabonese Republic	7.39 (5.1-11.13)	0.91 (0.62-1.37)	18.89 (11.62-29.69)	1.1 (0.69-1.72)	0.4 (0.25-0.55)
HL	Georgia	584.78 (377.42-763.75)	10.39 (6.67-13.68)	376.6 (288.92-476.64)	10.62 (7.91-13.8)	-0.23 (-0.81-0.35)
HL	Global	270221.94 (241909.37-286756.81)	5.24 (4.7-5.55)	361579.01 (307336.25-414718)	4.42 (3.75-5.09)	-0.48 (-0.6--0.36)
HL	Grand Duchy of Luxembourg	46.75 (41.49-51.73)	11.23 (9.88-12.55)	73.93 (63.75-85.33)	10.33 (8.9-11.9)	-0.31 (-0.76-0.14)
HL	Greenland	2.08 (1.17-2.85)	3.34 (1.89-4.51)	1.85 (1.22-3.25)	3.02 (1.94-5.4)	-0.13 (-0.32-0.07)
HL	Grenada	1.56 (1.24-1.98)	1.99 (1.6-2.5)	1.85 (1.48-2.3)	1.66 (1.33-2.07)	-0.82 (-1.06--0.59)
HL	Guam	1.96 (1.23-2.87)	1.38 (0.86-2.07)	2.99 (1.39-4.04)	1.89 (0.87-2.57)	1.56 (1.27-1.85)
HL	Hashemite Kingdom of Jordan	54.28 (36.18-90.4)	1.59 (1.08-2.61)	412.89 (248.54-588.73)	3.19 (1.92-4.56)	2.85 (2.48-3.23)
HL	Hellenic Republic	5570.16 (4941.17-6237.55)	50.79 (44.73-57.53)	4278.32 (3796.37-4792)	40.79 (35.67-47.1)	-0.77 (-1.34--0.2)
HL	High SDI	134609.93 (130619.59-138707.82)	14.12 (13.7-14.55)	132507.04 (125199.4-140138.92)	10.67 (10.06-11.31)	-0.82 (-1.1--0.53)
HL	High-middle SDI	75510.1 (68602.47-80832.63)	6.74 (6.12-7.22)	95848.63 (85469.11-104168.78)	6.71 (6.02-7.35)	0.07 (-0.09-0.23)
HL	Hungary	1185.27 (1018.24-1375.85)	11.02 (9.43-12.77)	867.77 (687.09-1101.97)	8.56 (6.8-10.78)	-1.34 (-1.6--1.07)
HL	Independent State of Papua New Guinea	11.81 (4.28-19.86)	0.31 (0.12-0.53)	27.98 (14.29-43.22)	0.27 (0.14-0.42)	-0.64 (-0.77--0.5)
HL	Independent State of Samoa	5.78 (3.35-11.02)	3.82 (2.23-7.52)	11.86 (6.61-22.81)	5.88 (3.31-11.47)	1.43 (1.32-1.55)
HL	Ireland	600.76 (517.61-702.52)	16.65 (14.36-19.48)	1087.66 (892.2-1298.63)	20.93 (17.14-25.14)	1.93 (1.36-2.51)
HL	Islamic Republic of Afghanistan	229.44 (135.78-423.12)	2.84 (1.65-5.25)	906.05 (572.04-1442.29)	3.38 (2.14-5.36)	0.6 (0.54-0.66)
HL	Islamic Republic of Iran	840.87 (576.44-1207.43)	1.56 (1.08-2.27)	4747.23 (2292.72-6210.94)	5.51 (2.69-7.27)	4.8 (4.42-5.17)
HL	Islamic Republic of Mauritania	7.96 (4.47-11.24)	0.44 (0.25-0.62)	17.12 (10.38-29.31)	0.42 (0.26-0.7)	-0.61 (-0.81--0.42)
HL	Islamic Republic of Pakistan	3628.09 (2229.14-5238.09)	3.59 (2.28-5.44)	10174.45 (6689.12-14616.33)	4.23 (2.81-6.15)	0.32 (0.18-0.45)
HL	Jamaica	40.19 (33.14-48.45)	1.7 (1.42-2.03)	60.24 (43.21-83)	1.95 (1.4-2.68)	0.08 (-0.31-0.47)
HL	Japan	1900.1 (1817.15-1984.86)	1.27 (1.21-1.34)	3900.18 (3591.72-4155.76)	2.35 (2.16-2.54)	2.69 (2.41-2.97)
HL	Kingdom of Bahrain	20.83 (12.99-30.43)	4.41 (2.79-6.12)	149.87 (92.18-225.1)	8.98 (5.39-13.39)	2.72 (2.44-3.01)
HL	Kingdom of Belgium	1904.99 (1615.2-2284.84)	17.92 (15.06-21.66)	2098.07 (1678.31-2607.38)	17.38 (13.84-21.84)	-0.13 (-0.38-0.11)
HL	Kingdom of Bhutan	11.9 (6.41-19.58)	2.06 (1.17-3.54)	19.69 (9.35-46.34)	2.42 (1.17-5.64)	0.45 (0.31-0.58)
HL	Kingdom of Cambodia	109.39 (70.17-200.38)	1.26 (0.82-2.31)	239.31 (118.42-531.99)	1.38 (0.69-3.06)	0.23 (0-0.46)
HL	Kingdom of Denmark	582.57 (506.65-673.92)	10.55 (9.15-12.3)	609.5 (505.78-751.09)	9.3 (7.65-11.38)	0.15 (-0.44-0.75)
HL	Kingdom of Eswatini	8.28 (5.29-14.21)	1.31 (0.84-2.23)	18.73 (11.04-30.21)	1.69 (1-2.7)	0.88 (0.69-1.07)
HL	Kingdom of Lesotho	12.37 (7.21-21.83)	0.98 (0.57-1.76)	27.43 (17.53-44.09)	1.54 (0.98-2.47)	1.98 (1.74-2.21)
HL	Kingdom of Morocco	625.01 (389.74-857.95)	2.56 (1.62-3.46)	1403.08 (836.27-2183.58)	3.67 (2.19-5.69)	1.19 (1.13-1.25)
HL	Kingdom of Norway	490.78 (443.05-542.33)	10.64 (9.54-11.82)	1050.24 (938.96-1167.98)	16.68 (14.9-18.59)	1.78 (1.33-2.22)
HL	Kingdom of Saudi Arabia	248.94 (79.33-421.01)	1.71 (0.57-2.83)	2215.06 (533.88-3988.45)	5.03 (1.2-8.92)	4.11 (3.56-4.66)
HL	Kingdom of Spain	7997.24 (6778.85-9487.1)	19.36 (16.32-23.07)	9577.66 (7712.01-11995.09)	18.75 (14.91-24.01)	0.13 (-0.24-0.5)
HL	Kingdom of Sweden	759.14 (637.39-902.21)	8.12 (6.75-9.81)	1059.62 (852.67-1305.62)	9.55 (7.58-12.15)	0.73 (0.16-1.31)
HL	Kingdom of Thailand	736 (503.44-1213.27)	1.23 (0.84-2.07)	1856.62 (1145.56-3709)	2.67 (1.66-5.16)	1.83 (1.5-2.17)
HL	Kingdom of the Netherlands	2508.53 (2170.69-2921.68)	15.68 (13.47-18.39)	3287.78 (2687.74-4020.5)	18.63 (14.96-23.18)	1.26 (0.93-1.6)
HL	Kingdom of Tonga	0.23 (0.12-0.38)	0.28 (0.14-0.44)	0.35 (0.19-0.56)	0.36 (0.2-0.57)	0.67 (0.55-0.79)
HL	Kyrgyz Republic	185.27 (138.79-240.78)	4.2 (3.16-5.41)	193.91 (136.21-265.38)	2.79 (1.97-3.79)	0.6 (-0.44-1.65)
HL	Lao People's Democratic Republic	39.22 (25.41-69.81)	1.12 (0.72-2.03)	69.39 (35.33-159.87)	0.92 (0.48-2.13)	-0.77 (-0.94--0.6)
HL	Lebanese Republic	213.2 (118.13-296.14)	7.4 (4.15-10.24)	850.72 (511.87-1165.58)	14.51 (8.7-19.93)	2.5 (2.32-2.67)

Cause	Location	1990		2021		EAPC No. (95% CI)
		Prevalence cases (95% UI)	ASPR per 100,000 No. (95% UI)	Prevalence cases (95% UI)	ASPR per 100,000 No. (95% UI)	
HL	Low SDI	11349.42 (6571.35-14095.53)	2.53 (1.49-3.22)	25377.35 (14742.55-34679.74)	2.35 (1.38-3.23)	-0.41 (-0.49--0.32)
HL	Low-middle SDI	20144.72 (13640.4-26423.66)	1.86 (1.25-2.48)	41552.99 (30423.16-61267.26)	2.11 (1.55-3.12)	0.4 (0.31-0.49)
HL	Malaysia	319.19 (162.33-451.29)	1.91 (0.98-2.64)	1123.12 (633.15-1447.19)	3.29 (1.85-4.23)	1.6 (1.38-1.81)
HL	Middle SDI	28253 (19795.52-32870.4)	1.7 (1.18-1.98)	65887.98 (46901.28-80894.43)	2.6 (1.84-3.18)	1.48 (1.35-1.6)
HL	Mongolia	25 (13.66-47.8)	1.28 (0.72-2.44)	85.88 (51.95-125.51)	2.57 (1.55-3.76)	2.73 (2.44-3.03)
HL	Montenegro	134.64 (84.75-186.02)	20.62 (13-28.45)	137.63 (90.99-194.27)	21.56 (14.17-30.66)	0.73 (0.52-0.93)
HL	New Zealand	170.95 (143.3-207.02)	4.65 (3.9-5.64)	345.55 (287.79-411.81)	5.78 (4.77-6.97)	0.61 (-0.4-1.64)
HL	North Macedonia	181.96 (114.49-256.73)	8.8 (5.54-12.36)	286.39 (185.44-475.37)	12.2 (7.84-20.49)	1.4 (0.99-1.81)
HL	Northern Mariana Islands	0.12 (0.07-0.18)	0.24 (0.13-0.37)	0.19 (0.08-0.26)	0.35 (0.16-0.5)	0.9 (0.57-1.23)
HL	Palestine	76.47 (45.01-109.22)	4.4 (2.49-6.17)	348.79 (199.39-478.46)	6.93 (3.96-9.37)	1.55 (1.2-1.9)
HL	People's Democratic Republic of Algeria	1195.36 (705.26-1611.91)	4.78 (2.99-6.55)	3749.33 (2331.84-5381.15)	8.52 (5.28-12.2)	1.94 (1.91-1.98)
HL	People's Republic of Bangladesh	2551.44 (1781.58-3998.75)	2.5 (1.69-4.07)	4927.15 (2675.71-10325.27)	2.85 (1.56-5.98)	0.52 (0.31-0.74)
HL	People's Republic of China	12934.65 (5607.01-17906.06)	1.16 (0.5-1.61)	23818.09 (14286.95-31455.07)	1.36 (0.83-1.79)	0.49 (0.35-0.64)
HL	Plurinational State of Bolivia	116.96 (66.32-158.18)	2.11 (1.22-2.85)	229.28 (130.72-381.59)	1.96 (1.12-3.22)	-0.28 (-0.39--0.18)
HL	Portuguese Republic	1087.12 (925.14-1283.97)	10.4 (8.83-12.35)	1511.95 (1215.92-1872.82)	13.61 (10.67-17.36)	0.79 (0.39-1.2)
HL	Principality of Andorra	7.8 (4.58-11.87)	12.25 (7.29-18.68)	15.03 (8.56-22.97)	16.48 (9.45-24.99)	1.12 (0.68-1.57)
HL	Principality of Monaco	15.84 (8.26-22.6)	50.75 (26.72-74.36)	25.72 (15.36-43.3)	72.94 (41.51-123.08)	1.16 (0.96-1.36)
HL	Puerto Rico	309.66 (265.12-364.53)	8.42 (7.22-9.9)	369.72 (292.74-470.07)	10.87 (8.57-13.82)	0.95 (0.53-1.37)
HL	Republic of Albania	163.86 (100.12-233.2)	4.77 (2.97-6.81)	222.77 (138.63-347.67)	7.98 (4.89-12.23)	2.43 (1.76-3.11)
HL	Republic of Angola	76.03 (51.9-113.99)	0.95 (0.63-1.46)	259.06 (161.84-410.73)	0.99 (0.62-1.53)	0.19 (0.05-0.34)
HL	Republic of Armenia	107.79 (84.63-128.07)	3.05 (2.41-3.62)	119.03 (94.74-143.98)	3.9 (3.07-4.77)	1.17 (0.73-1.62)
HL	Republic of Austria	1576.77 (1371.47-1802.02)	18.32 (15.91-21)	1606.37 (1303.73-1937.4)	16.14 (12.86-19.79)	-0.2 (-0.54-0.15)
HL	Republic of Azerbaijan	323.62 (200.9-488.22)	4.21 (2.62-6.3)	652.46 (387.44-1056.55)	5.98 (3.48-9.63)	1.25 (0.94-1.56)
HL	Republic of Belarus	1119.27 (906.33-1393.56)	10.5 (8.4-13.21)	2055.99 (1547.67-2707.68)	20.69 (15.44-27.58)	2.45 (2.24-2.67)
HL	Republic of Benin	18.42 (9.41-26.24)	0.46 (0.24-0.63)	40.38 (24.96-68.49)	0.34 (0.22-0.57)	-1.33 (-1.53--1.12)
HL	Republic of Botswana	14.21 (8.52-25.23)	1.31 (0.79-2.26)	30.94 (16.93-54.44)	1.24 (0.69-2.12)	-0.13 (-0.39-0.13)
HL	Republic of Bulgaria	969.91 (799.7-1160.17)	11.15 (9.1-13.36)	757.9 (588.77-979.83)	11.2 (8.67-14.33)	0.08 (-0.23-0.39)
HL	Republic of Burundi	147.44 (85.86-204.63)	2.85 (1.67-3.95)	331.1 (169.62-522.25)	2.67 (1.38-4.13)	-0.18 (-0.26--0.1)
HL	Republic of Cabo Verde	0.2 (0.06-0.34)	0.06 (0.02-0.11)	0.35 (0.2-0.53)	0.06 (0.03-0.09)	0.1 (-0.4-0.6)
HL	Republic of Cameroon	44.94 (23.07-65.17)	0.5 (0.26-0.7)	116.05 (71.6-185.09)	0.4 (0.25-0.62)	-1.2 (-1.39--1)
HL	Republic of Chad	21.83 (11.43-32.09)	0.43 (0.23-0.62)	55.02 (35.4-93.24)	0.39 (0.25-0.63)	-0.58 (-0.78--0.39)
HL	Republic of Chile	225.99 (186.35-279.8)	1.69 (1.4-2.08)	1565.76 (1255.48-1935.36)	7.83 (6.22-9.81)	5.99 (5.01-6.98)
HL	Republic of Colombia	836.4 (733.7-961.79)	2.63 (2.34-2.98)	2428 (1945.24-3036.89)	4.66 (3.72-5.84)	1.94 (1.75-2.13)
HL	Republic of Costa Rica	285.52 (242.23-334.73)	9.38 (7.99-10.95)	458.37 (377.4-554.14)	8.99 (7.38-10.98)	-0.34 (-0.71-0.03)
HL	Republic of Côte d'Ivoire	151.02 (85.97-221.16)	1.38 (0.85-2.06)	422.95 (233.2-639.39)	1.56 (0.91-2.37)	0.42 (0.29-0.56)
HL	Republic of Croatia	1068.48 (931.73-1233.13)	19.64 (17.04-22.85)	761.17 (624.79-916.16)	14.77 (11.97-17.84)	-0.88 (-1.24--0.52)
HL	Republic of Cuba	1343.08 (1073.55-1611.49)	11.1 (8.92-13.27)	1560.72 (1285.85-1888.45)	12.84 (10.42-15.65)	0.13 (-0.08-0.33)
HL	Republic of Cyprus	68.58 (43.86-107.98)	8.5 (5.45-13.32)	256.12 (165.78-409.7)	16.62 (10.85-26.75)	2.57 (1.93-3.22)
HL	Republic of Djibouti	8.42 (4.62-13.33)	2.16 (1.2-3.33)	33.84 (18.54-55.83)	2.66 (1.48-4.33)	0.57 (0.39-0.76)
HL	Republic of Ecuador	90.38 (74.17-110.33)	1 (0.84-1.2)	555.06 (417.93-737.21)	3.02 (2.28-4)	4.24 (3.61-4.87)
HL	Republic of El Salvador	75.44 (49.77-102.29)	1.58 (1.04-2.17)	215.38 (142.21-295)	3.28 (2.15-4.48)	2.77 (2.54-3)
HL	Republic of Equatorial Guinea	3.28 (2.17-5.13)	1.02 (0.67-1.6)	16.49 (9.33-27.29)	1.16 (0.66-1.89)	0.42 (0.01-0.84)
HL	Republic of Estonia	176.54 (135.87-225.25)	11.21 (8.55-14.48)	197.75 (148.55-251.63)	15.41 (11.34-19.78)	1.12 (0.62-1.62)
HL	Republic of Fiji	7.07 (4-15.68)	0.94 (0.54-2.11)	14.29 (8.49-23.07)	1.53 (0.91-2.46)	2.12 (1.73-2.51)
HL	Republic of Finland	699.48 (607.84-819.77)	12.98 (11.17-15.3)	936.34 (771.51-1108.65)	15.65 (12.66-19.26)	1.45 (0.81-2.1)
HL	Republic of Ghana	11.67 (4.04-19)	0.09 (0.03-0.15)	22.1 (11.14-33.75)	0.07 (0.03-0.1)	-1.65 (-2.05--1.24)
HL	Republic of Guatemala	69.72 (56.17-80.24)	1.01 (0.86-1.12)	143.47 (119.43-169.76)	0.91 (0.76-1.08)	-0.08 (-0.27-0.1)
HL	Republic of Guinea	81.64 (46.59-117.17)	1.45 (0.93-2.13)	187.53 (112.74-291.15)	1.47 (0.94-2.27)	0.07 (0.03-0.11)
HL	Republic of Guinea-Bissau	5.69 (3.02-8.47)	0.67 (0.36-0.98)	8.73 (5.74-13.73)	0.48 (0.32-0.75)	-1.29 (-1.4--1.17)
HL	Republic of Guyana	10.43 (8.31-13.36)	1.47 (1.21-1.83)	13.58 (10.32-17.75)	1.71 (1.3-2.23)	0.49 (0.22-0.76)
HL	Republic of Haiti	102.33 (67.9-151.57)	1.9 (1.26-2.88)	228.68 (121.39-354.43)	1.86 (0.98-2.85)	0.09 (-0.03-0.22)
HL	Republic of Honduras	31.94 (20.28-43.27)	0.79 (0.51-1.06)	74.7 (44.82-117.24)	0.8 (0.49-1.22)	-0.11 (-0.25-0.03)
HL	Republic of Iceland	45.98 (38.97-56.07)	17.35 (14.7-21.21)	64.45 (51.78-78.67)	17.42 (13.89-21.47)	0.44 (0.01-0.86)
HL	Republic of India	14998.8 (8757.74-18802.17)	1.83 (1.07-2.3)	29043.16 (19547.6-41202.79)	1.94 (1.31-2.75)	0.17 (0.03-0.31)
HL	Republic of Indonesia	1810.71 (1253.09-3312.5)	1.01 (0.7-1.89)	3251.98 (1863.86-7044.3)	1.1 (0.63-2.39)	0.08 (-0.08-0.24)
HL	Republic of Iraq	534.18 (255.03-786.55)	3.19 (1.54-4.58)	2131.03 (1026.88-3323.84)	5.06 (2.47-7.78)	1.56 (1.42-1.71)
HL	Republic of Italy	12716.84 (11483.96-14342.42)	20.91 (18.81-23.7)	13409.71 (12013.38-14818)	21.48 (18.98-23.96)	0.68 (0.16-1.19)
HL	Republic of Kazakhstan	561.5 (453.05-802.05)	3.32 (2.69-4.74)	864.72 (703.2-1054.88)	4.52 (3.67-5.54)	0.94 (0.7-1.19)
HL	Republic of Kenya	257.17 (155.89-382.58)	1.25 (0.76-1.86)	821.01 (472.51-1261.65)	1.66 (0.96-2.49)	1.1 (0.98-1.22)
HL	Republic of Kiribati	0.11 (0.04-0.17)	0.16 (0.06-0.24)	0.2 (0.08-0.33)	0.16 (0.06-0.27)	0.27 (0.21-0.32)
HL	Republic of Korea	356.03 (255.92-618.38)	0.9 (0.65-1.56)	1047.39 (613.44-1468.32)	1.67 (1-2.35)	2.94 (2.52-3.36)
HL	Republic of Latvia	254.34 (205.16-309.89)	9.54 (7.64-11.72)	235.07 (176.22-301.59)	12.35 (9.24-15.98)	0.49 (0.2-0.78)
HL	Republic of Liberia	9.66 (4.75-14.03)	0.45 (0.22-0.64)	22.39 (13.58-36.74)	0.43 (0.27-0.68)	-0.14 (-0.34-0.06)
HL	Republic of Lithuania	461.39 (385.44-539.09)	12.22 (10.13-14.35)	335.4 (269.19-410.87)	11.89 (9.41-14.72)	-0.64 (-0.99--0.3)
HL	Republic of Madagascar	256.22 (165.69-353.04)	2.3 (1.49-3.1)	693.52 (398.45-1055.3)	2.45 (1.4-3.7)	0.22 (0.16-0.29)
HL	Republic of Malawi	144.1 (81.51-207.58)	1.49 (0.86-2.07)	302.46 (164.51-456.11)	1.57 (0.89-2.28)	0.19 (0.13-0.25)
HL	Republic of Maldives	2.94 (1.81-4.63)	1.57 (1-2.41)	14.21 (8.73-25.47)	2.55 (1.6-4.32)	2.03 (1.72-2.34)
HL	Republic of Mali	160.92 (87.45-224.26)	2.04 (1.24-2.98)	406.29 (248.96-620.84)	1.82 (1.17-2.96)	-0.25 (-0.38--0.13)
HL	Republic of Malta	58.78 (49.3-69.93)	15.48 (12.92-18.57)	96.83 (79.53-118.88)	21.01 (16.91-26.13)	1.62 (1.26-1.97)
HL	Republic of Mauritius	34.37 (31-38.09)	3.1 (2.8-3.38)	79.24 (69.31-89.79)	5.6 (4.89-6.34)	-0.24 (-1.38-0.91)
HL	Republic of Moldova	625.06 (547.25-720.4)	13.99 (12.26-16.14)	480.5 (398.07-570.71)	12.54 (10.31-15.17)	-0.4 (-0.88-0.08)
HL	Republic of Mozambique	351.67 (193.83-495.83)	2.77 (1.48-4.07)	915.71 (446.59-1469.59)	3.17 (1.59-4.93)	0.82 (0.65-0.99)
HL	Republic of Namibia	19.48 (10.86-27.63)	1.6 (0.93-2.3)	56.13 (30.79-89.9)	2.31 (1.31-3.66)	1.14 (1.06-1.23)
HL	Republic of Nauru	0.04 (0.02-0.05)	0.4 (0.21-0.57)	0.05 (0.02-0.08)	0.45 (0.21-0.71)	0.23 (0.11-0.35)
HL	Republic of Nicaragua	41.23 (28.05-61.78)	1.2 (0.8-1.83)	112.74 (75.23-171.86)	1.68 (1.12-2.52)	1.41 (1.26-1.56)
HL	Republic of Niue	0.01 (0-0.01)	0.37 (0.19-0.57)	0.01 (0.01-0.02)	0.77 (0.47-1.05)	0.98 (0.5-1.47)
HL	Republic of Palau	0.03 (0.01-0.06)	0.18 (0.09-0.34)	0.04 (0.02-0.07)	0.27 (0.11-0.47)	1.43 (1.2-1.66)
HL	Republic of Panama	37.22 (33.04-42.75)	1.6 (1.44-1.81)	127 (102.33-157.45)	2.9 (2.34-3.59)	1.98 (1.79-2.17)
HL	Republic of Paraguay	52.24 (33.12-82.3)	1.4 (0.87-2.25)	157.9 (103.33-226.9)	2.12 (1.39-3.04)	1.42 (1.28-1.56)
HL	Republic of Peru	201.32 (141.82-310.07)	1.01 (0.72-1.53)	845.87 (531.69-1226.46)	2.28 (1.43-3.28)	3.21 (3.02-3.4)
HL	Republic of Poland	3618.06 (3371.38-3925.33)	9.28 (8.61-10.12)	3389 (3007.57-3769.33)	9.15 (8-10.27)	-0.62 (-1.07--0.18)
HL	Republic of Rwanda	224.17 (141.16-309.89)	3.34 (2.1-4.48)	357.82 (191.37-590.37)	2.69 (1.46-4.38)	-1.07 (-1.31--0.82)

Cause	Location	1990		2021		EAPC No. (95% CI)
		Prevalence cases (95% UI)	ASPR per 100,000 No. (95% UI)	Prevalence cases (95% UI)	ASPR per 100,000 No. (95% UI)	
HL	Republic of San Marino	10.88 (6.43-15.94)	41.56 (24.66-60.9)	12.61 (6.99-21.1)	38.93 (21.44-63.99)	0.38 (0.04-0.72)
HL	Republic of Senegal	35.07 (17.59-51.24)	0.52 (0.26-0.74)	69.68 (42.99-117.59)	0.46 (0.29-0.75)	-0.57 (-0.71--0.43)
HL	Republic of Serbia	836.45 (526.94-1190.9)	8.24 (5.15-11.72)	1258.98 (824.55-1895.33)	11.96 (7.82-18.38)	1.39 (1.21-1.57)
HL	Republic of Seychelles	2.33 (1.65-3.23)	3.21 (2.23-4.37)	4.26 (3.06-6.26)	3.82 (2.72-5.68)	0.63 (0.4-0.86)
HL	Republic of Sierra Leone	14.99 (6.7-22.5)	0.41 (0.19-0.59)	30.53 (19.3-49.08)	0.37 (0.23-0.58)	-0.43 (-0.53--0.33)
HL	Republic of Singapore	61.28 (52.36-70.86)	2.08 (1.78-2.41)	173.55 (142.34-210.81)	2.82 (2.25-3.58)	2.43 (1.87-3)
HL	Republic of Slovenia	293.01 (246.34-339.02)	14.05 (11.81-16.28)	296.86 (224.51-392.71)	13.5 (10.2-17.89)	0.55 (0.07-1.03)
HL	Republic of South Africa	400.09 (291.52-582.76)	1.13 (0.81-1.6)	860.9 (531.69-1095.43)	1.43 (0.88-1.82)	0.86 (0.63-1.08)
HL	Republic of South Sudan	118.21 (72.35-169.25)	2.2 (1.32-3.15)	264.18 (149.63-399.86)	2.89 (1.66-4.38)	0.84 (0.5-1.18)
HL	Republic of Sudan	294.67 (183.5-525.34)	1.68 (1.07-3.08)	1112.53 (621.92-1872.02)	2.56 (1.46-4.32)	1.54 (1.38-1.7)
HL	Republic of Suriname	6.12 (3.81-8.91)	1.63 (1.04-2.38)	13.56 (8.11-18.63)	2.28 (1.36-3.13)	1.14 (0.98-1.3)
HL	Republic of Tajikistan	33.26 (20.79-50.56)	0.66 (0.43-0.98)	69.47 (43.07-109.48)	0.67 (0.41-1.05)	-0.37 (-0.69--0.04)
HL	Republic of the Congo	18.65 (13.08-28.88)	1 (0.69-1.54)	54.44 (34.21-82.92)	1.08 (0.69-1.64)	0.16 (0-0.31)
HL	Republic of the Gambia	11.03 (5.82-16.72)	1.22 (0.71-1.85)	34.34 (21.21-57.64)	1.43 (0.92-2.41)	0.2 (-0.1-0.49)
HL	Republic of the Marshall Islands	0.12 (0.07-0.16)	0.33 (0.19-0.44)	0.21 (0.11-0.31)	0.37 (0.2-0.54)	0.1 (0.02-0.19)
HL	Republic of the Niger	32.84 (16.7-48.07)	0.49 (0.26-0.71)	67.15 (38.4-116.25)	0.34 (0.19-0.58)	-1.49 (-1.66--1.33)
HL	Republic of the Philippines	298.41 (194-394.15)	0.49 (0.32-0.67)	698.63 (420.52-891.04)	0.6 (0.36-0.76)	0.49 (0.27-0.72)
HL	Republic of the Union of Myanmar	490.81 (293.9-914.28)	1.24 (0.76-2.32)	633.17 (342.5-1414.37)	1.09 (0.59-2.43)	-0.8 (-1.06--0.54)
HL	Republic of Trinidad and Tobago	21.31 (18.86-24.52)	1.82 (1.62-2.08)	34.95 (25.95-44.99)	2.45 (1.82-3.16)	0.95 (0.69-1.22)
HL	Republic of Tunisia	509.55 (338.71-700.73)	6.09 (4.11-8.64)	1430.63 (908.98-2223.71)	11.93 (7.57-18.26)	2.05 (1.96-2.13)
HL	Republic of Turkey	1499.16 (1050.56-2542.97)	2.67 (1.89-4.54)	4678.45 (3178.34-7818.03)	5.32 (3.63-8.98)	2.64 (2.43-2.84)
HL	Republic of Uganda	475.81 (285.62-698.07)	3.04 (1.86-4.49)	1782.55 (1014.11-2684.41)	4.2 (2.48-6.2)	0.65 (0.47-0.82)
HL	Republic of Uzbekistan	585.84 (424.18-798.68)	2.85 (2.09-3.83)	1126.45 (812.78-1502.88)	3.19 (2.3-4.26)	0.41 (0.23-0.59)
HL	Republic of Vanuatu	0.39 (0.17-0.64)	0.3 (0.13-0.5)	0.89 (0.5-1.33)	0.3 (0.17-0.44)	-0.36 (-0.53--0.19)
HL	Republic of Yemen	136.88 (85.33-220.69)	1.36 (0.84-2.27)	562.58 (314.77-894.47)	1.89 (1.09-2.89)	1.22 (1.04-1.41)
HL	Republic of Zambia	192.12 (121.12-260.68)	2.64 (1.66-3.52)	540.51 (309.49-812.31)	2.84 (1.67-4.15)	0.11 (0.01-0.22)
HL	Republic of Zimbabwe	119.37 (63.96-176.73)	1.42 (0.81-2.21)	330.21 (189.33-484.69)	2.29 (1.35-3.32)	2.04 (1.62-2.47)
HL	Romania	1725.97 (1450.49-2024.69)	7.33 (6.13-8.65)	1735.23 (1283.34-2246.86)	8.78 (6.48-11.43)	0.47 (0.23-0.72)
HL	Russian Federation	22937.67 (22047.83-23967.54)	15.05 (14.46-15.75)	23919.14 (21886.7-25855.85)	15.78 (14.47-16.98)	0.01 (-0.28-0.31)
HL	Saint Kitts and Nevis	0.03 (0.02-0.03)	0.07 (0.06-0.08)	0.3 (0.24-0.38)	0.47 (0.37-0.6)	5.84 (3.84-7.88)
HL	Saint Lucia	3.8 (3.34-4.48)	3.09 (2.74-3.57)	8.04 (6.37-10.17)	4.18 (3.33-5.28)	0.77 (0.53-1.01)
HL	Saint Vincent and the Grenadines	3.12 (2.74-3.63)	3.02 (2.67-3.48)	3.55 (3.01-4.21)	2.97 (2.51-3.54)	-0.3 (-0.52--0.07)
HL	Slovak Republic	541.65 (329.16-734.73)	9.95 (6.06-13.44)	767.69 (495.14-1125.39)	13.59 (8.74-20.1)	1.26 (1.14-1.39)
HL	Socialist Republic of Viet Nam	1205.34 (734.31-1871.12)	1.9 (1.21-3.05)	4524.5 (2802.24-6950.76)	4.39 (2.67-6.76)	2.93 (2.8-3.06)
HL	Solomon Islands	0.9 (0.31-1.5)	0.33 (0.12-0.54)	2.35 (1.3-3.58)	0.37 (0.2-0.57)	0.25 (0.07-0.42)
HL	State of Eritrea	83.47 (50.16-116.46)	2.74 (1.68-3.71)	191.64 (110.53-286.75)	2.98 (1.73-4.39)	0.34 (0.29-0.39)
HL	State of Israel	549.11 (470.79-637.95)	11.33 (9.74-13.08)	1198.45 (966.3-1455.62)	12.67 (10.18-15.45)	0.72 (0.17-1.27)
HL	State of Kuwait	176.99 (147.79-210.6)	10.02 (8.49-11.86)	244.32 (188.23-302.85)	4.89 (3.78-6.09)	1.38 (-0.59-3.39)
HL	State of Libya	293.87 (178.26-419.44)	7.45 (4.69-10.98)	1037.75 (594.1-1504.03)	13.88 (8.03-19.84)	2.35 (2.05-2.65)
HL	State of Qatar	8.53 (4.96-15.79)	2 (1.24-3.55)	162.39 (87.85-254.95)	5.07 (2.75-7.77)	3.96 (3.53-4.4)
HL	Sultanate of Oman	62.01 (30.6-95.58)	3.49 (1.75-5.27)	333.64 (154.08-493.2)	6.78 (3.26-9.82)	2.73 (2.31-3.16)
HL	Swiss Confederation	1228.98 (1042.32-1473.07)	16.84 (14.18-20.31)	874.94 (702.89-1090.38)	9.12 (7.21-11.56)	-2.06 (-2.7--1.41)
HL	Syrian Arab Republic	13.53 (9-18.12)	0.12 (0.08-0.16)	41.62 (21.6-60.05)	0.28 (0.15-0.41)	3.07 (2.71-3.44)
HL	Taiwan (Province of China)	500.81 (434.17-575.17)	2.41 (2.11-2.76)	877.18 (739.63-1032.38)	2.93 (2.42-3.59)	0.03 (-0.39-0.45)
HL	Togolese Republic	15.25 (8.02-21.62)	0.48 (0.26-0.66)	32.31 (20.99-50.5)	0.42 (0.27-0.63)	-0.84 (-1.03--0.65)
HL	Tokelau	0.01 (0-0.01)	0.36 (0.19-0.54)	0.01 (0.01-0.02)	0.85 (0.48-1.24)	1.26 (0.63-1.9)
HL	Turkmenistan	94.23 (78.67-114.7)	2.56 (2.17-3.05)	468.79 (350.07-637.47)	8.73 (6.54-11.86)	4.77 (4.32-5.22)
HL	Tuvalu	0.03 (0.02-0.05)	0.37 (0.21-0.52)	0.05 (0.03-0.07)	0.39 (0.22-0.57)	0.01 (-0.09-0.11)
HL	Ukraine	4097.13 (3163.96-5495.82)	7.92 (6.09-10.58)	3800.76 (2629.35-5365.65)	9.12 (6.39-12.84)	0.13 (-0.23-0.49)
HL	Union of the Comoros	11.32 (4.89-17.11)	2.65 (1.17-4)	21.97 (12.82-32.99)	2.89 (1.7-4.35)	-0.17 (-0.61-0.26)
HL	United Arab Emirates	44.07 (27.03-63.2)	2.58 (1.55-3.68)	296.38 (179.47-468.07)	3.34 (2.03-4.72)	1.05 (0.79-1.32)
HL	United Kingdom of Great Britain and Northern Ireland	11461.17 (11095.61-11804.77)	18.59 (17.95-19.18)	15296.15 (14681.71-15933.37)	20.85 (19.97-21.75)	1.01 (0.65-1.38)
HL	United Mexican States	2312.6 (2227.53-2411.04)	2.98 (2.88-3.09)	5120.91 (4582.69-5671.83)	3.79 (3.4-4.2)	0.92 (0.7-1.13)
HL	United Republic of Tanzania	648.25 (402.04-915.7)	2.65 (1.6-3.73)	1576.23 (897.27-2461.57)	2.72 (1.53-4.17)	0.12 (0.02-0.22)
HL	United States of America	68105.79 (66221.91-69960.01)	24.64 (23.94-25.32)	48542.49 (46304.03-50596.62)	12.47 (11.97-13.01)	-2.25 (-2.64--1.87)
HL	United States Virgin Islands	0.91 (0.59-1.59)	0.87 (0.57-1.52)	1.45 (0.77-2.25)	2.13 (1.1-3.48)	3.9 (3.53-4.27)

Table S6. age, period, and cohort of deaths of HL

PeriodRR

Period	Rate Ratio	CI Lo	CI Hi
1990-1995	1.34513808818114	1.3203772113636	1.37036330277692
1995-2000	1.26166254482669	1.23862158024601	1.28513211977334
2000-2005	1.13016542359085	1.10954458665342	1.15116949786829
2005-2010	1	1	1
2010-2015	0.885728349567688	0.869378396051758	0.9023857882721
2015-2020	0.813696277790321	0.798639781329906	0.829036629489308
2020-2025	0.751484619429873	0.737492934199312	0.765741754329865

Cohort RR

Cohort	Rate Ratio	CILo	CIHi
1900	3.52511744079347	2.85781451962797	4.34823634845412

1905	3.24982140807579	2.95478134597141	3.57432173408945
1910	3.06156204635797	2.87875511758675	3.25597759477266
1915	2.87688878885885	2.74185479714803	3.0185730885788
1920	2.61727558290823	2.51037331723662	2.72873019715972
1925	2.37839831272614	2.29164895836043	2.46843152540506
1930	2.14258013978304	2.07079583902726	2.21685284897479
1935	1.91067952685072	1.85016620163331	1.97317205940941
1940	1.68504665220983	1.63361444135752	1.73809813885096
1945	1.48560657849437	1.44113187489134	1.53145381385182
1950	1.2730684051675	1.2363415602097	1.31088626023445
1955	1.11351001632777	1.08265387317997	1.14524557402674
1960	1	1	1
1965	0.875235545837786	0.851006954106995	0.900153937639452
1970	0.763752746123629	0.741880612344211	0.786269714433165
1975	0.714525008091967	0.693321415343533	0.736377062485306
1980	0.686197733463721	0.665011359904827	0.708059076581994
1985	0.638816478846983	0.618125694999266	0.660199854087836
1990	0.582339441121731	0.562343796597328	0.603046084509046
1995	0.552613210383818	0.53203208642497	0.573990494337932
2000	0.528802324378402	0.506835045719908	0.551721710306775
2005	0.502465460305676	0.478612665441665	0.527507015651609
2010	0.455128287308896	0.428908510231262	0.482950916028783
2015	0.377838236431525	0.348990816746994	0.409070170500165
2020	0.33998851576061	0.29822596218143	0.387599355882974

LongAgeRR

Age	Rate Ratio	CILo	CIHi
＜5	0.338175279857364	0.32005769179247	0.357318454888941
5-9	0.605334412645699	0.579025206832045	0.632839031547381
10-14	0.764079948626239	0.733822242525153	0.795585271282731
15-19	1.10331450517298	1.06435234467344	1.14370293203853
20-24	1.26679064949727	1.22500435642271	1.3100023205958
25-29	1.42571817040103	1.38156066126178	1.47128704399791
30-34	1.21292784655897	1.17588435001061	1.25113831215197
35-39	1.17294776252536	1.13813072726075	1.20882990034418
40-44	1.13632575362055	1.10304509048678	1.17061054845136
45-49	1	1	1
50-54	1.09952107929908	1.06657702666876	1.13348269613392
55-59	1.33233076209377	1.29276445239493	1.3731080370696
60-64	1.42459951265464	1.38106033124771	1.46951130630356
65-69	1.68702002776793	1.63420430850866	1.74154269406335
70-74	2.11445796799657	2.04627694277309	2.18491075424289
75-79	2.22012055364962	2.14334865141923	2.29964232346136
80-84	2.42180184509315	2.33019064550122	2.51701472933989
85-89	2.41893816568614	2.31064375949651	2.53230807447707
90-94	1.80416650158678	1.68093279188742	1.93643480641067

Coefficients

	Parameter	SD	CILo	CIHi
Intercept	-12.05750245	0.003085353	-12.06354974	-12.05145515
LAT	0.014863444	0.000287684	0.014299582	0.015427305
NetDrift	-0.020482211	0.000259485	-0.020990801	-0.01997362
CAT	0.035345655	0.000149587	0.035052464	0.035638845
THETAa	-0.000123628	8.14E-06	-0.000139579	-0.000107676
THETAp	2.42E-05	2.96E-05	-3.38E-05	8.23E-05
THETAc	5.93E-05	6.24E-06	4.70E-05	7.15E-05

NetDrift

Net Drift (%/year)	CILo	CIHi
-2.027387508	-2.077202782	-1.977546891

Table S7. age, period, and cohort of DALYs of HL

PeriodRR

Period	Rate Ratio	CI Lo	CI Hi
1990-1995	1.32482930451554	1.29701657283746	1.35323844186769
1995-2000	1.24790650304175	1.22204163193948	1.27431881175961
2000-2005	1.12813963047827	1.10500150029462	1.15176226051849
2005-2010	1	1	1
2010-2015	0.886816865382216	0.868305485449863	0.905722888896513
2015-2020	0.812423247117301	0.795015796890748	0.830211846151934
2020-2025	0.744525531898635	0.727825716939758	0.761608520759133

Cohort RR

Cohort	Rate Ratio	CILo	CIHi
1900	3.52511744079347	2.85781451962797	4.34823634845412
1905	3.24982140807579	2.95478134597141	3.57432173408945
1910	3.06156204635797	2.87875511758675	3.25597759477266
1915	2.87688878885885	2.74185479714803	3.0185730885788
1920	2.61727558290823	2.51037331723662	2.72873019715972
1925	2.37839831272614	2.29164895836043	2.46843152540506
1930	2.14258013978304	2.07079583902726	2.21685284897479
1935	1.91067952685072	1.85016620163331	1.97317205940941
1940	1.68504665220983	1.63361444135752	1.73809813885096
1945	1.48560657849437	1.44113187489134	1.53145381385182
1950	1.2730684051675	1.2363415602097	1.31088626023445
1955	1.11351001632777	1.08265387317997	1.14524557402674
1960	1	1	1
1965	0.875235545837786	0.851006954106995	0.900153937639452
1970	0.763752746123629	0.741880612344211	0.786269714433165
1975	0.714525008091967	0.693321415343533	0.736377062485306
1980	0.686197733463721	0.665011359904827	0.708059076581994
1985	0.638816478846983	0.618125694999266	0.660199854087836
1990	0.582339441121731	0.562343796597328	0.603046084509046
1995	0.552613210383818	0.53203208642497	0.573990494337932
2000	0.528802324378402	0.506835045719908	0.551721710306775
2005	0.502465460305676	0.478612665441665	0.527507015651609
2010	0.455128287308896	0.428908510231262	0.482950916028783
2015	0.377838236431525	0.348990816746994	0.409070170500165
2020	0.33998851576061	0.29822596218143	0.387599355882974

LongAgeRR

Age	Rate Ratio	CILo	CIHi
＜5	0.661121853730696	0.629240247838021	0.694618799388727
5-9	1.13354971426759	1.08682808137523	1.18227986259817
10-14	1.34425554118538	1.29215499381975	1.39845681721651
15-19	1.82867335319034	1.76322869870228	1.89654707590093
20-24	1.97492322065937	1.9074221495761	2.04481306268062
25-29	2.06096871057654	1.99341103799983	2.13081594563534
30-34	1.61777822835339	1.56487907107518	1.67246558824256
35-39	1.43155224965554	1.38527942977403	1.47937073159899
40-44	1.25938473846985	1.21847672045439	1.30166616470055
45-49	1	1	1
50-54	0.98089704904691	0.946573175335083	1.01646554740825
55-59	1.04159998487526	1.00431329545908	1.08027100049116
60-64	0.963959917510875	0.927108279568251	1.00227637164486
65-69	0.965529426772466	0.9261490594453	1.00658426897493
70-74	0.992927921230258	0.949110400325432	1.03876836290129
75-79	0.83868755980877	0.795788337765243	0.883899385800614
80-84	0.716292978923053	0.671751506039628	0.763787839761381
85-89	0.576949100264941	0.527922959541541	0.630528106952568
90-94	0.385468025237829	0.328037395800007	0.452953231500894

Coefficients

	Parameter	SD	CILo	CIHi
Intercept	-8.452356409	0.005362358	-8.46286663	-8.441846188
LAT	-0.009913017	0.000423919	-0.010743899	-0.009082135
NetDrift	-0.020199745	0.000359455	-0.020904276	-0.019495214
CAT	0.010286729	0.000292272	0.009713875	0.010859582

THETAa	-0.00035725	1.46E-05	-0.000385933	-0.000328568
THETAp	-3.33E-05	3.36E-05	-9.91E-05	3.25E-05
THETAc	5.96E-05	1.11E-05	3.78E-05	8.13E-05

NetDrift

Net Drift (%/year)	CILo	CIHi
-1.999709732	-2.068729657	-1.930641162

Table S8. age, period, and cohort of incidence of HL

PeriodRR

Period	Rate Ratio	CI Lo	CI Hi
1990-1995	1.13744430008041	1.11551224116149	1.15980756467388
1995-2000	1.1235821288418	1.1023152383056	1.14525932000469
2000-2005	1.05779555225451	1.03808004254394	1.07788550450055
2005-2010	1	1	1
2010-2015	0.964586324016682	0.946981128499118	0.982518815295359
2015-2020	0.893552058848344	0.877046027472681	0.910368734207607
2020-2025	0.815422882597456	0.799965925114325	0.831178499719873

Cohort RR

Cohort	Rate Ratio	CILo	CIHi
1900	0.838808063860202	0.507223982274709	1.3871563502213
1905	0.761682935700749	0.590668133746896	0.982211264483606
1910	0.813362083840166	0.694160957804874	0.95303239398661
1915	0.872431432399415	0.786846218073994	0.967325745177464
1920	0.918345730750133	0.849040995302554	0.993307609235602
1925	0.976590077746948	0.919843705525634	1.03683720856555
1930	0.98291302134207	0.935427101930499	1.0328095107892
1935	0.974376156871102	0.93270667822494	1.01790725556479
1940	0.970313732868695	0.933179636393195	1.00892550959704
1945	0.986196185079847	0.951664453354911	1.02198092199135
1950	1.00335735999754	0.971935651111646	1.03579490134948
1955	0.987237646586719	0.959229135173344	1.01606397793782
1960	1	1	1
1965	0.952286358254089	0.927590476787102	0.97763973521795
1970	0.859436978698097	0.836987648403602	0.882488435477533
1975	0.827882018717986	0.805662343261238	0.850714499255587
1980	0.830265689617597	0.807260674353236	0.853926293273821
1985	0.824612926447669	0.801034916413326	0.848884941881514
1990	0.779851905429905	0.756424088216754	0.804005324362943
1995	0.761415400642869	0.736472800741845	0.787202747680782
2000	0.746763273147619	0.718877753072201	0.775730482323239
2005	0.783477351050535	0.747666690824596	0.821003218602886
2010	0.807332001169951	0.758579118393903	0.859218167635653
2015	0.745800999502221	0.682990365868878	0.814387960144809
2020	0.712366383132288	0.605234005039177	0.838462246985162

LongAgeRR

Age	Rate Ratio	CILo	CIHi
＜5	0.0991740278869959	0.0926492686820753	0.106158288643173
5-9	0.255311841662643	0.243501501107269	0.26769500884701
10-14	0.40073903423225	0.384856590684215	0.417276921961734
15-19	0.932724138637411	0.902770571763814	0.963671552892075
20-24	1.57775538993759	1.53247432811736	1.62437440210514
25-29	1.64077603842131	1.59554803005439	1.68728609703197
30-34	1.41754360097488	1.37884565780837	1.45732761987209
35-39	1.20605047303077	1.17290620852748	1.24013133609708
40-44	1.08548511482358	1.05513042296529	1.11671307059102
45-49	1	1	1
50-54	1.05337361263891	1.02199775225475	1.0857127281894
55-59	0.991691855735641	0.959738698975252	1.02470884812967
60-64	1.08286998962341	1.04590873496803	1.12113741402383
65-69	1.14762791108111	1.10474376776054	1.19217673883079

70-74	1.09138327433049	1.04444712867131	1.14042867158208
75-79	1.1336407035079	1.07744464826287	1.19276776465677
80-84	0.839968008756366	0.78418558246502	0.89971847418606
85-89	1.03649824363289	0.950954395543458	1.1297372556337
90-94	1.13391628801841	0.991962852172988	1.29618376879422

Coefficients

	Parameter	SD	CILo	CIHi
Intercept	-11.43511895	0.00394914938393214	-11.44285928	-11.42737862
LAT	0.0140416020774647	0.000327928030931619	0.0133988631368387	0.0146843410180907
NetDrift	-0.011063466	0.000287432636110813	-0.011626834	-0.010500098
CAT	0.0251050677854746	0.000208626131444106	0.0246961605678441	0.025513975003105
THETAa	-0.000494539	1.0654615012933e-05	-0.000515422	-0.000473656
THETAp	-0.00020796	3.03171975860357e-05	-0.000267382	-0.000148538
THETAc	5.8664940743773e-05	8.28702933721033e-06	4.24223632428408e-05	7.49075182447053e-05

NetDrift

Net Drift (%/year)	CILo	CIHi
-1.100249064	-1.155950324	-1.044516415

Table S9. age, period, and cohort of prevalence of HL

PeriodRR

Period	Rate Ratio	CI Lo	CI Hi
1990-1995	0.982379493731577	0.961483479602734	1.00372964297115
1995-2000	1.005714643	0.985221847595882	1.02663369190041
2000-2005	0.99457687277286	0.975090027709155	1.01445315585741
2005-2010	1	1	1
2010-2015	1.0179931789055	0.998790948947326	1.03756458084682
2015-2020	0.968954795792031	0.950086477560884	0.988197830895043
2020-2025	0.898956582353136	0.880423298198413	0.917880000006443

Cohort RR

Cohort	Rate Ratio	CILo	CIHi
1900	0.838808063860202	0.507223982274709	1.3871563502213
1905	0.761682935700749	0.590668133746896	0.982211264483606
1910	0.813362083840166	0.694160957804874	0.95303239398661
1915	0.872431432399415	0.786846218073994	0.967325745177464
1920	0.918345730750133	0.849040995302554	0.993307609235602
1925	0.976590077746948	0.919843705525634	1.03683720856555
1930	0.98291302134207	0.935427101930499	1.0328095107892
1935	0.974376156871102	0.93270667822494	1.01790725556479
1940	0.970313732868695	0.933179636393195	1.00892550959704
1945	0.986196185079847	0.951664453354911	1.02198092199135
1950	1.00335735999754	0.971935651111646	1.03579490134948
1955	0.987237646586719	0.959229135173344	1.01606397793782
1960	1	1	1
1965	0.952286358254089	0.927590476787102	0.97763973521795
1970	0.859436978698097	0.836987648403602	0.882488435477533
1975	0.827882018717986	0.805662343261238	0.850714499255587
1980	0.830265689617597	0.807260674353236	0.853926293273821
1985	0.824612926447669	0.801034916413326	0.848884941881514
1990	0.779851905429905	0.756424088216754	0.804005324362943
1995	0.761415400642869	0.736472800741845	0.787202747680782
2000	0.746763273147619	0.718877753072201	0.775730482323239
2005	0.783477351050535	0.747666690824596	0.821003218602886
2010	0.807332001169951	0.758579118393903	0.859218167635653
2015	0.745800999502221	0.682990365868878	0.814387960144809
2020	0.712366383132288	0.605234005039177	0.838462246985162

LongAgeRR

Age	Rate Ratio	CILo	CIHi
＜5	0.0991740278869959	0.0926492686820753	0.106158288643173
5-9	0.255311841662643	0.243501501107269	0.26769500884701
10-14	0.40073903423225	0.384856590684215	0.417276921961734
15-19	0.932724138637411	0.902770571763814	0.963671552892075
20-24	1.57775538993759	1.53247432811736	1.62437440210514

25-29	1.64077603842131	1.59554803005439	1.68728609703197
30-34	1.41754360097488	1.37884565780837	1.45732761987209
35-39	1.20605047303077	1.17290620852748	1.24013133609708
40-44	1.08548511482358	1.05513042296529	1.11671307059102
45-49	1	1	1
50-54	1.05337361263891	1.02199775225475	1.0857127281894
55-59	0.991691855735641	0.959738698975252	1.02470884812967
60-64	1.08286998962341	1.04590873496803	1.12113741402383
65-69	1.14762791108111	1.10474376776054	1.19217673883079
70-74	1.09138327433049	1.04444712867131	1.14042867158208
75-79	1.1336407035079	1.07744464826287	1.19276776465677
80-84	0.839968008756366	0.78418558246502	0.89971847418606
85-89	1.03649824363289	0.950954395543458	1.1297372556337
90-94	1.13391628801841	0.991962852172988	1.29618376879422

Coefficients

	Parameter	SD	CILo	CIHi
Intercept	-9.945498655	0.00534458027803589	-9.955974033	-9.935023278
LAT	0.0122760630082206	0.000394309245602171	0.0115032168868404	0.0130489091296009
NetDrift	-0.002267351	0.000369832730497793	-0.002992223	-0.001542479
CAT	0.0145434138882372	0.000293637037204244	0.0139678852953169	0.0151189424811576
THETAa	-0.000607744	1.35399147374838e-05	-0.000634282	-0.000581206
THETAp	-0.000313656	3.19341366019777e-05	-0.000376246	-0.000251065
THETAc	-6.86E-05	1.20849676895019e-05	-9.23E-05	-4.49E-05

NetDrift

Net Drift (%/year)	CILo	CIHi
-0.226478238	-0.298775079	-0.154128972

Table S10. The trends in slope index of inequality and concentration index of HL crude incidence, prevalence, mortality and DALYs rates from 1990 to 2021.

year	Incidence			deaths			DALYs			Prevalence		
	lower	SII	upper	lower	SII	upper	lower	SII	upper	lower	SII	upper
1990	0.110906444	1.112314472	2.1137225	-0.624241906	0.358343816	1.340929538	-0.939221859	0.043744899	1.026711658	0.755386527	1.750105107	2.744823686
1991	0.103341809	1.097596997	2.091852186	-0.651696972	0.325658979	1.30301493	-0.985168932	0.004377516	0.993923964	0.762470537	1.746974439	2.731478342
1992	0.064419032	1.066596097	2.068773162	-0.700308762	0.282816461	1.265941683	-1.037968107	-0.041675705	0.954616697	0.722333867	1.716274576	2.710215284
1993	0.166543048	1.15341032	2.140277591	-0.636956703	0.346474127	1.329904957	-0.969898246	0.019146656	1.008191559	0.804763866	1.800569265	2.796374664
1994	0.214684695	1.197635455	2.180586214	-0.619571218	0.355744483	1.331060183	-0.927244669	0.042489627	1.012223924	0.87292374	1.856017009	2.839110278
1995	0.247397643	1.215859202	2.184320761	-0.623830388	0.344174636	1.312179659	-0.926445902	0.033187392	0.992820685	0.896619434	1.872128476	2.847637518
1996	0.237619938	1.205004811	2.172389683	-0.665276957	0.302886305	1.271049566	-0.957922846	-0.00379971	0.950323425	0.895109537	1.867319434	2.83952933
1997	0.103443344	1.07005176	2.036660177	-0.821764525	0.146331292	1.114427108	-1.122844138	-0.163595387	0.795653363	0.742593889	1.731973572	2.721353255
1998	0.02267245	0.986970044	1.951267637	-0.912305793	0.048395428	1.009096649	-1.219124469	-0.264457822	0.690208825	0.674938741	1.659692986	2.644447232
1999	0.012694189	0.967997948	1.923301708	-0.938756749	0.020058853	0.978874456	-1.257736053	-0.298020851	0.661694351	0.68496436	1.65249514	2.62002592
2000	-0.008101619	0.958484216	1.925070052	-0.966030753	-0.009401195	0.947228363	-1.300745934	-0.338176114	0.624393707	0.676758095	1.649213006	2.621667917
2001	0.011486103	0.964521509	1.917556916	-0.978806703	-0.016163016	0.946480389	-1.307840896	-0.343171703	0.62149749	0.702293671	1.676962833	2.651631994
2002	-0.027143452	0.934204778	1.895553009	-1.017208588	-0.05065596	0.915896668	-1.353725158	-0.382996839	0.587731481	0.656039475	1.64466358	2.633287684
2003	-0.044691154	0.925045881	1.894782915	-1.041742269	-0.068642714	0.90445684	-1.378009184	-0.400440364	0.577128455	0.641420529	1.624931708	2.608442887
2004	0.029025912	0.993105002	1.957184093	-0.989610645	-0.023485873	0.942638898	-1.315746701	-0.357426065	0.60089457	0.717654636	1.691494779	2.665334923
2005	0.118172795	1.072272549	2.026372303	-0.919268195	0.029672513	0.978613221	-1.256397815	-0.302440392	0.65151703	0.806149388	1.769729034	2.73330868
2006	0.122769045	1.07200815	2.021247254	-0.953765258	-0.006190535	0.941384188	-1.295577054	-0.340793807	0.61398944	0.81727518	1.774993879	2.732712579
2007	0.092644848	1.03914589	1.985646931	-1.015589964	-0.067328418	0.880933129	-1.363615679	-0.40759702	0.54842164	0.783445856	1.742515626	2.701585396
2008	0.128021229	1.077599158	2.027177086	-0.990788942	-0.046281204	0.898226534	-1.346809819	-0.395598318	0.555613183	0.83608214	1.78168407	2.727286001
2009	0.122032172	1.088751068	2.055469965	-0.993928464	-0.049692582	0.894543301	-1.35691233	-0.403383081	0.550146167	0.843615756	1.79478047	2.745945185
2010	0.085402182	1.055620961	2.02583974	-1.03621476	-0.087459468	0.861295824	-1.411468017	-0.450725335	0.510017348	0.801917694	1.761424829	2.720931964
2011	0.087279052	1.054211216	2.021143381	-1.035501361	-0.092221662	0.851058036	-1.428848207	-0.466575399	0.49569741	0.779712373	1.74463212	2.709551868
2012	0.068362	1.030586	1.992810	-1.062344	-0.114496	0.833350	-1.445431	-0.487656	0.470117	0.753719	1.720737	2.687755

year	Incidence			deaths			DALYs			Prevalence		
	lower	SII	upper	lower	SII	upper	lower	SII	upper	lower	SII	upper
	885	766	647	052	769	514	232	896	441	135	438	741
2013	0.092759536	1.038477458	1.984195379	-1.071935174	-0.124561362	0.822812451	-1.456737948	-0.502696682	0.451344585	0.756368878	1.709492317	2.662615757
2014	0.092512737	1.040067297	1.987621858	-1.058184538	-0.115914345	0.826355848	-1.466946932	-0.508993135	0.448960662	0.749125569	1.704559679	2.659993789
2015	0.044577812	0.988011056	1.931444301	-1.09366741	-0.155348408	0.782970594	-1.511191121	-0.556928325	0.397334471	0.70442585	1.652908512	2.601391174
2016	0.006256982	0.947016024	1.887775066	-1.138401577	-0.189206937	0.759987703	-1.550228761	-0.592301193	0.365626374	0.672321277	1.612693766	2.553066256
2017	-0.02645038	0.911779319	1.850009018	-1.16582998	-0.221768283	0.722293415	-1.595246842	-0.632578023	0.330090797	0.632008179	1.56826477	2.50452136
2018	-0.075410302	0.852580231	1.780570765	-1.213701444	-0.276533065	0.660635313	-1.641010284	-0.681788725	0.277432835	0.579811683	1.511066054	2.442320426
2019	-0.14568699	0.790697973	1.727082936	-1.281248858	-0.338754738	0.603739383	-1.70537691	-0.747160273	0.211056363	0.502649956	1.437725134	2.372800312
2020	-0.212353956	0.723196559	1.658747074	-1.321207321	-0.376905325	0.567396672	-1.742512568	-0.792046171	0.158420226	0.404643261	1.353498638	2.302354014
2021	-0.23197604	0.71436565	1.66070734	-1.315063338	-0.374107891	0.566847556	-1.761174463	-0.800863432	0.159447598	0.396870032	1.348100129	2.299330225
	lower	CI	upper	lower	CI	upper	lower	CI	upper	lower	CI	upper
1990	-0.228592929	-0.09	0.048592929	-0.05701505	0.04	0.13701505	-0.040874343	0.07	0.180874343	-0.336311515	-0.17	-0.003688485
2021	-0.128592929	0.01	0.148592929	0.01298495	0.11	0.20701505	0.039125657	0.15	0.260874343	-0.216311515	-0.05	0.116311515

Table S11. Predicted trends of HL deaths,incidence, prevalence, and DALYs in the next decade (2022-2050).

Year	DALYs			Prevalence			Deaths			incidence			Type
	lower	Value	upper	lower	Value	upper	lower	Value	upper	lower	Value	upper	
1990	21.9196416671294	21.9196416671294	21.9196416671294	2.291732406	2.291732406	2.291732406	0.541007170342326	0.541007170342326	0.541007170342326	0.752438796	0.752438796	0.752438796	Actual
1991	21.0842570793795	21.0842570793795	21.0842570793795	2.270796599	2.270796599	2.270796599	0.52981573279068	0.52981573279068	0.52981573279068	0.758309857	0.758309857	0.758309857	Actual
1992	20.7703406992067	20.7703406992067	20.7703406992067	2.237748915	2.237748915	2.237748915	0.516749224721026	0.516749224721026	0.516749224721026	0.768249527	0.768249527	0.768249527	Actual
1993	20.0765162334891	20.0765162334891	20.0765162334891	2.325582475	2.325582475	2.325582475	0.50911060282522	0.50911060282522	0.50911060282522	0.76432521	0.76432521	0.76432521	Actual
1994	19.7562267464776	19.7562267464776	19.7562267464776	2.292104374	2.292104374	2.292104374	0.489055795423312	0.489055795423312	0.489055795423312	0.776211088	0.776211088	0.776211088	Actual
1995	20.0154760103702	20.0154760103702	20.0154760103702	2.411158483	2.411158483	2.411158483	0.495552522133682	0.495552522133682	0.495552522133682	0.799428358	0.799428358	0.799428358	Actual
1996	19.8735148610074	19.8735148610074	19.8735148610074	2.454533052	2.454533052	2.454533052	0.487421839624722	0.487421839624722	0.487421839624722	0.785085477	0.785085477	0.785085477	Actual
1997	19.4448091822003	19.4448091822003	19.4448091822003	2.478507811	2.478507811	2.478507811	0.484533100883689	0.484533100883689	0.484533100883689	0.802399452	0.802399452	0.802399452	Actual
1998	18.9043322108413	18.9043322108413	18.9043322108413	2.499889586	2.499889586	2.499889586	0.474667176028987	0.474667176028987	0.474667176028987	0.784917014	0.784917014	0.784917014	Actual
1999	18.7390869847924	18.7390869847924	18.7390869847924	2.498247672	2.498247672	2.498247672	0.468258965020653	0.468258965020653	0.468258965020653	0.752933661	0.752933661	0.752933661	Actual
2000	17.2162112788872	17.2162112788872	17.2162112788872	2.494991457	2.494991457	2.494991457	0.448786875929706	0.448786875929706	0.448786875929706	0.770869712	0.770869712	0.770869712	Actual
2001	16.6634714446037	16.6634714446037	16.6634714446037	2.386295963	2.386295963	2.386295963	0.427656279285481	0.427656279285481	0.427656279285481	0.752769719	0.752769719	0.752769719	Actual
2002	16.6958949958546	16.6958949958546	16.6958949958546	2.420077759	2.420077759	2.420077759	0.42547606522873	0.42547606522873	0.42547606522873	0.748812268	0.748812268	0.748812268	Actual
2003	16.7577988959291	16.7577988959291	16.7577988959291	2.495469163	2.495469163	2.495469163	0.42259620649737	0.42259620649737	0.42259620649737	0.763143451	0.763143451	0.763143451	Actual
2004	16.3132632936241	16.3132632936241	16.3132632936241	2.584607932	2.584607932	2.584607932	0.40969855860904	0.40969855860904	0.40969855860904	0.751181184	0.751181184	0.751181184	Actual
2005	15.6549928711255	15.6549928711255	15.6549928711255	2.624240732	2.624240732	2.624240732	0.402066665069292	0.402066665069292	0.402066665069292	0.730942722	0.730942722	0.730942722	Actual
2006	15.2987346728196	15.2987346728196	15.2987346728196	2.687105125	2.687105125	2.687105125	0.395665889634501	0.395665889634501	0.395665889634501	0.727405325	0.727405325	0.727405325	Actual
2007	15.1974347263808	15.1974347263808	15.1974347263808	2.701817765	2.701817765	2.701817765	0.388868684341274	0.388868684341274	0.388868684341274	0.73588299	0.73588299	0.73588299	Actual
2008	15.0717090142973	15.0717090142973	15.0717090142973	2.788073007	2.788073007	2.788073007	0.386927807508495	0.386927807508495	0.386927807508495	0.738435964	0.738435964	0.738435964	Actual

Year	DALYs			Prevalence			Deaths			incidence			Type
	lower	Value	upper	lower	Value	upper	lower	Value	upper	lower	Value	upper	
2009	14.9600144584169	14.9600144584169	14.9600144584169	2.744605562	2.744605562	2.744605562	0.380775976782602	0.380775976782602	0.380775976782602	0.73340817	0.73340817	0.73340817	Actual
2010	14.4624855205469	14.4624855205469	14.4624855205469	2.765298539	2.765298539	2.765298539	0.371311076881817	0.371311076881817	0.371311076881817	0.73409218	0.73409218	0.73409218	Actual
2011	14.0969448106889	14.0969448106889	14.0969448106889	2.78911824	2.78911824	2.78911824	0.367003361928043	0.367003361928043	0.367003361928043	0.719331495	0.719331495	0.719331495	Actual
2012	13.9617191795501	13.9617191795501	13.9617191795501	2.774418631	2.774418631	2.774418631	0.356467601045648	0.356467601045648	0.356467601045648	0.722177098	0.722177098	0.722177098	Actual
2013	13.7120270391801	13.7120270391801	13.7120270391801	2.78025648	2.78025648	2.78025648	0.357218648432991	0.357218648432991	0.357218648432991	0.722090949	0.722090949	0.722090949	Actual
2014	13.5553725698967	13.5553725698967	13.5553725698967	2.872004548	2.872004548	2.872004548	0.355703855828663	0.355703855828663	0.355703855828663	0.726678247	0.726678247	0.726678247	Actual
2015	13.0175444946242	13.0175444946242	13.0175444946242	2.873616498	2.873616498	2.873616498	0.339686804779357	0.339686804779357	0.339686804779357	0.724926345	0.724926345	0.724926345	Actual
2016	12.6702999208503	12.6702999208503	12.6702999208503	2.913775323	2.913775323	2.913775323	0.331988638333684	0.331988638333684	0.331988638333684	0.729903253	0.729903253	0.729903253	Actual
2017	12.4132796734686	12.4132796734686	12.4132796734686	2.887577659	2.887577659	2.887577659	0.318859780423515	0.318859780423515	0.318859780423515	0.723095458	0.723095458	0.723095458	Actual
2018	12.0375373316916	12.0375373316916	12.0375373316916	2.943188188	2.943188188	2.943188188	0.308482932615999	0.308482932615999	0.308482932615999	0.715794746	0.715794746	0.715794746	Actual
2019	11.7183462157587	11.7183462157587	11.7183462157587	2.923121763	2.923121763	2.923121763	0.302208773389558	0.302208773389558	0.302208773389558	0.71760853	0.71760853	0.71760853	Actual
2020	11.1441814651771	11.1441814651771	11.1441814651771	2.910857839	2.910857839	2.910857839	0.285901529518423	0.285901529518423	0.285901529518423	0.703554178	0.703554178	0.703554178	Actual
2021	11.0871482282525	11.0871482282525	11.0871482282525	2.915771998	2.915771998	2.915771998	0.282353814068784	0.282353814068784	0.282353814068784	0.68425029	0.68425029	0.68425029	Actual
2022	10.1025755683449	10.7377129560308	11.3728503437167	2.83975186865952	2.93590230743541	3.03205274621129	0.25900632102822	0.273167640888741	0.287328960749262	0.658984986985857	0.68425029	0.709515592014143	Forecast
2023	9.49005777617349	10.3882776838091	11.2864975914447	2.82005536232583	2.95603261687081	3.0920098714158	0.243954337100845	0.263981467708697	0.284008598316549	0.64851975602704	0.68425029	0.71998082297296	Forecast
2024	8.93875218632885	10.0388424115874	11.1389326368459	2.80962548117634	2.97616292630622	3.14270037143609	0.230267169027998	0.254795294528654	0.27932342002931	0.640489501876907	0.68425029	0.728011077123093	Forecast
2025	8.41913236399389	9.68940713936568	10.9596819147375	2.80399235818985	2.99629323574162	3.1885941132934	0.217286481627569	0.24560912134861	0.273931761069652	0.633719684471714	0.68425029	0.734780894528286	Forecast
2026	7.91976149322667	9.33997186714398	10.7601822410613	2.80142462800771	3.01642354517703	3.23142246234634	0.204757274309324	0.236422948168566	0.268088622027809	0.62775535560628	0.68425029	0.74074522339372	Forecast
2027	7.43477407852756	8.99053659492227	10.546299111317	2.8010343410668	3.03655385461243	3.27207336815807	0.192548767245906	0.227236774988523	0.26192478273114	0.622363190143292	0.68425029	0.746137388856708	Forecast
2028	6.96068574652446	8.64110132270056	10.3215168988767	2.8022940145971	3.05668416404784	3.31107431349858	0.180583271221101	0.218050601808479	0.255517932395857	0.617404582248762	0.68425029	0.751095996751238	Forecast
2029	6.49522623520763	8.29166605047886	10.0881058657501	2.80485996439328	3.07681447348325	3.34876898257321	0.168810167412732	0.208864428628436	0.248918689844139	0.61278922255408	0.68425029	0.75571135644592	Forecast
2030	6.03681861519946	7.94223077825715	9.84764294131484	2.80849346659099	3.09694478291865	3.38539609924631	0.15719429586683	0.199678255448392	0.242162215029954	0.608454381957571	0.68425029	0.760046197042429	Forecast
2031	5.58431473381863	7.59279550603544	9.60127627825225	2.81302070779768	3.11707509235406	3.42112947691043	0.145710056834925	0.190492082268348	0.235274107701772	0.604354387782129	0.68425029	0.764146191217871	Forecast
2032	5.13684782853312	7.24336023381373	9.34987263909434	2.8183104729418	3.13720540178946	3.45610033063712	0.13433812457475	0.181305909088305	0.22827369360186	0.600454760845764	0.68425029	0.768045818154236	Forecast
2033	4.69374451107494	6.89392496159203	9.09410541210911	2.82426082096511	3.15733571122487	3.49041060148462	0.123063484906949	0.172119735908261	0.221175986909573	0.596728714253813	0.68425029	0.771771864746187	Forecast
2034	4.25446927110457	6.54448968937032	8.83451010763607	2.83079068349545	3.17746602066027	3.5241413578251	0.111874197842864	0.162933562728218	0.213992927613572	0.593154945795148	0.68425029	0.775345633204852	Forecast
2035	3.81858791889737	6.19505441714861	8.57152091539985	2.83783433060832	3.19759633009568	3.55735832958303	0.100760582485588	0.153747389548174	0.20673419661076	0.589716183718878	0.68425029	0.778784395281122	Forecast
2036	3.38574261986574	5.8456191449269	8.30549566998806	2.84533759142154	3.21772663953108	3.59011568764063	0.0897146603880173	0.14456121636813	0.199407772348244	0.586398193625831	0.68425029	0.782102385374169	Forecast
2037	2.95563432196161	5.4961838727052	8.03673342344878	2.85325519386294	3.23785694896649	3.62245870407004	0.0787297637460039	0.135375043188087	0.19202032263017	0.583189079443428	0.68425029	0.785311499556572	Forecast
2038	2.52801006427567	5.14674860048349	7.76548713669131	2.86154884337943	3.2579872584019	3.65442567342436	0.0678002524249591	0.126188870008043	0.184577487591127	0.580078778571005	0.68425029	0.788421800428995	Forecast
2039	2.1026536	4.797313	7.49197305116862	2.870185	3.278117	3.686049	0.0569213	0.1170026	0.1770840	0.577058	0.684	0.791441	Forecast

Year	DALYs			Prevalence			Deaths			incidence			Type
	lower	Value	upper	lower	Value	upper	lower	Value	upper	lower	Value	upper	
	0535495	32826178		80420235	5678373	33147225	05004444	97	88651555	689081119	25029	889918881	
2040	1.67937836785284	4.44787805604008	7.21637774422731	2.8791378312715 2	3.2982478772727 1	3.7173579232739	0.0460887614687907	0.107816523647956	0.169544285827121	0.5741213890628 66	0.68425029	0.7943791899371 34	Forecast
2041	1.25802203598376	4.09844278381837	6.93886353165297	2.8883803523694 8	3.3183781867081 1	3.7483760210467 5	0.0352990027494281	0.0986303504679125	0.161961698186397	0.57126042171256	0.68425029	0.79724015728744	Forecast
2042	0.838442355829531	3.74900751159666	6.65957266736379	2.8978918323498	3.3385084961435 2	3.7791251599372 4	0.0245488570865511	0.0894441772878688	0.154339497489187	0.5684701282729 81	0.68425029	0.8000304507270 19	Forecast
2043	0.420513826519944	3.39957223937495	6.37863065222997	2.9076532722305 6	3.3586388055789 2	3.8096243389272 8	0.0138355262541383	0.0802580041078252	0.146680481961512	0.5657455164109 57	0.68425029	0.8027550625890 43	Forecast
2044	0.00412506165473392	3.05013696715325	6.09614887265176	2.9176478097525 8	3.3787691150143 3	3.8398904202760 8	0.0031565267289819	0.0710718309277817	0.138987135126581	0.5630821552566 41	0.68425029	0.8054184237433 59	Forecast
2045	-0.410823338	2.70070169493154	5.81222672772095	2.9278603973584 6	3.3988994244497 4	3.8699384515410 1	-0.007490358	0.061885657747738	0.131261673232972	0.5604760907865 85	0.68425029	0.8080244882134 15	Forecast
2046	-0.824420516	2.35126642270983	5.52695336113931	2.9382775400057	3.4190297338851 4	3.8997819277645 8	-0.018107115	0.0526994845676945	0.123506083870298	0.5579237769292 85	0.68425029	0.8105768020707 15	Forecast
2047	-1.236746783	2.00183115048813	5.24040908411086	2.9488870797617 9	3.4391600433205 5	3.9294330068793 1	-0.028695535	0.0435133113876508	0.115722157694675	0.55542201896356	0.68425029	0.81307856003644	Forecast
2048	-1.647874798	1.65239587826642	4.95266655404205	2.9596780173663 2	3.4592903527559 5	3.9589026881455 9	-0.039257238	0.0343271382076072	0.107911514709576	0.5529679266307 19	0.68425029	0.8155326523692 81	Forecast
2049	-2.057870546	1.30296060604471	4.66379175839691	2.9706403632898 8	3.4794206621913 6	3.9882009610928 4	-0.049793696	0.0251409650275637	0.100075626202319	0.5505588749975 25	0.68425029	0.8179417040024 75	Forecast
2050	-2.466794174	0.953525333823002	4.37384484168443	2.9817650125403 1	3.4995509716267 6	4.0173369307132 1	-0.060306249	0.01595479184752	0.0922158331829712	0.54819247155923	0.68425029	0.82030810744077	Forecast
2051	-2.874700682	0.604090061601298	4.08288080510599	2.9930436387464 4	3.5196812810621 7	4.0463189233779	-0.070796125	0.00676861866747647	0.0843333619840076	0.5458665284081 19	0.68425029	0.8226340505918 81	Forecast

The 2021 baseline ASPR of 2.92 in this table is derived from the BioWinford default country subset ('ALL'), which differs in country coverage from the complete GBD 2021 'Global' dataset. The global ASPR of 4.42 reported in the main analysis is based on all 204 countries/territories.
